# Prognostic indicators and outcomes of hospitalised COVID-19 patients with neurological disease: An individual patient data meta-analysis

**DOI:** 10.1371/journal.pone.0263595

**Published:** 2022-06-02

**Authors:** Bhagteshwar Singh, Suzannah Lant, Sofia Cividini, Jonathan W. S. Cattrall, Lynsey C. Goodwin, Laura Benjamin, Benedict D. Michael, Ayaz Khawaja, Aline de Moura Brasil Matos, Walid Alkeridy, Andrea Pilotto, Durjoy Lahiri, Rebecca Rawlinson, Sithembinkosi Mhlanga, Evelyn C. Lopez, Brendan F. Sargent, Anushri Somasundaran, Arina Tamborska, Glynn Webb, Komal Younas, Yaqub Al Sami, Heavenna Babu, Tristan Banks, Francesco Cavallieri, Matthew Cohen, Emma Davies, Shalley Dhar, Anna Fajardo Modol, Hamzah Farooq, Jeffrey Harte, Samuel Hey, Albert Joseph, Dileep Karthikappallil, Daniel Kassahun, Gareth Lipunga, Rachel Mason, Thomas Minton, Gabrielle Mond, Joseph Poxon, Sophie Rabas, Germander Soothill, Marialuisa Zedde, Konstantin Yenkoyan, Bruce Brew, Erika Contini, Lucette Cysique, Xin Zhang, Pietro Maggi, Vincent van Pesch, Jérome Lechien, Sven Saussez, Alex Heyse, Maria Lúcia Brito Ferreira, Cristiane N. Soares, Isabel Elicer, Laura Eugenín-von Bernhardi, Waleng Ñancupil Reyes, Rong Yin, Mohammed A. Azab, Foad Abd-Allah, Ahmed Elkady, Simon Escalard, Jean-Christophe Corvol, Cécile Delorme, Pierre Tattevin, Kévin Bigaut, Norbert Lorenz, Daniel Hornuss, Jonas Hosp, Siegbert Rieg, Dirk Wagner, Benjamin Knier, Paul Lingor, Andrea Sylvia Winkler, Athena Sharifi-Razavi, Shima T. Moein, SeyedAhmad SeyedAlinaghi, Saeidreza JamaliMoghadamSiahkali, Mauro Morassi, Alessandro Padovani, Marcello Giunta, Ilenia Libri, Simone Beretta, Sabrina Ravaglia, Matteo Foschi, Paolo Calabresi, Guido Primiano, Serenella Servidei, Nicola Biagio Mercuri, Claudio Liguori, Mariangela Pierantozzi, Loredana Sarmati, Federica Boso, Silvia Garazzino, Sara Mariotto, Kimani N. Patrick, Oana Costache, Alexander Pincherle, Frederikus A. Klok, Roger Meza, Verónica Cabreira, Sofia R. Valdoleiros, Vanessa Oliveira, Igor Kaimovsky, Alla Guekht, Jasmine Koh, Eva Fernández Díaz, José María Barrios-López, Cristina Guijarro-Castro, Álvaro Beltrán-Corbellini, Javier Martínez-Poles, Alba María Diezma-Martín, Maria Isabel Morales-Casado, Sergio García García, Gautier Breville, Matteo Coen, Marjolaine Uginet, Raphaël Bernard-Valnet, Renaud Du Pasquier, Yildiz Kaya, Loay H. Abdelnour, Claire Rice, Hamish Morrison, Sylviane Defres, Saif Huda, Noelle Enright, Jane Hassell, Lucio D’Anna, Matthew Benger, Laszlo Sztriha, Eamon Raith, Krishna Chinthapalli, Ross Nortley, Ross Paterson, Arvind Chandratheva, David J. Werring, Samir Dervisevic, Kirsty Harkness, Ashwin Pinto, Dinesh Jillella, Scott Beach, Kulothungan Gunasekaran, Ivan Rocha Ferreira Da Silva, Krishna Nalleballe, Jonathan Santoro, Tyler Scullen, Lora Kahn, Carla Y. Kim, Kiran T. Thakur, Rajan Jain, Thirugnanam Umapathi, Timothy R. Nicholson, James J. Sejvar, Eva Maria Hodel, The Brain Infections Global COVID-Neuro Network Study Group, Catrin Tudur Smith, Tom Solomon

**Affiliations:** 1 National Institute for Health Research Health Protection Research Unit in Emerging and Zoonotic Infections, Institute of Infection, Veterinary and Ecological Sciences, University of Liverpool, Liverpool, United Kingdom; 2 Tropical and Infectious Diseases Unit, Liverpool University Hospitals NHS Foundation Trust, Liverpool, United Kingdom; 3 Department of Infectious Diseases, Christian Medical College, Vellore, India; 4 Department of Health Data Science, University of Liverpool, Liverpool, United Kingdom; 5 Queen Square Institute of Neurology, University College London, London, United Kingdom; 6 The Walton Centre NHS Foundation Trust, Liverpool, United Kingdom; 7 Department of Neurology, Wayne State University, Detroit, Michigan, United States of America; 8 Instituto de Medicina Tropical, Universidade de São Paulo, São Paulo, Brazil; 9 Department of Medicine, King Saud University, Riyadh, Saudi Arabia; 10 Department of Clinical and Experimental Sciences, Neurology Unit, University of Brescia, Brescia, Italy; 11 Bangur Institute of Neurosciences, Institute of Post-Graduate Medical Education and Research, Kolkata, India; 12 Homerton University Hospital NHS Foundation Trust, London, United Kingdom of Great Britain and Northern Ireland; 13 Department of Neurovirology, National Institute of Mental Health and Neurosciences, Bangalore, India; 14 Department of Infection, Manchester University NHS Foundation Trust, Manchester, United Kingdom of Great Britain and Northern Ireland; 15 Neurology Unit, Neuromotor & Rehabilitation Department, Azienda USL-IRCCS di Reggio Emilia, Reggio Emilia, Italy; 16 Liverpool University Hospitals NHS Foundation Trust, Liverpool, United Kingdom of Great Britain and Northern Ireland; 17 Department of Virology, UK Health Security Agency, Manchester University NHS Foundation Trust, Manchester, United Kingdom of Great Britain and Northern Ireland; 18 Barts Health NHS Trust, London, United Kingdom of Great Britain and Northern Ireland; 19 Department of Infectious Diseases & Tropical Medicine, North Manchester General Hospital, Manchester University Foundation NHS Trust, Manchester, United Kingdom of Great Britain and Northern Ireland; 20 Warrington Hospital, Warrington and Halton Teaching Hospitals NHS Foundation Trust, Warrington, United Kingdom of Great Britain and Northern Ireland; 21 Malawi-Liverpool-Wellcome Trust Clinical Research Programme, Blantyre, Malawi; 22 Kingston Hospital NHS Foundation Trust, Kingston upon Thames, United Kingdom of Great Britain and Northern Ireland; 23 Institute of Clinical Neurosciences, University of Bristol, Bristol, United Kingdom of Great Britain and Northern Ireland; 24 North Manchester General Hospital, Manchester University Foundation NHS Trust, Manchester, United Kingdom of Great Britain and Northern Ireland; 25 Epsom and St Helier University Hospitals NHS Foundation Trust, United Kingdom of Great Britain and Northern Ireland; 26 King’s College Hospital NHS Foundation Trust, London, United Kingdom of Great Britain and Northern Ireland; 27 Regional Infectious Diseases Unit, NHS Lothian, Edinburgh, United Kingdom of Great Britain and Northern Ireland; 28 Yerevan State Medical University named after Mkhitar Heratsi, Neuroscience Laboratory, Cobrain Center, Yerevan, Armenia; 29 St Vincent’s Hospital, Sydney, Australia; 30 Saint-Luc University Hospital, Brussels, Belgium; 31 Université de Mons, Mons, Belgium; 32 AZ Glorieux, Ronse, Belgium; 33 Hospital da Restauração, Recife, Brazil; 34 Hospital Federal dos Servidores do Estado, Rio de Janeiro, Brazil; 35 Hospital Dr. Sótero del Río, Santiago, Chile; 36 Universidad de Chile - Hospital Barros Luco Trudeau, Santiago, Chile; 37 The 940th Hospital of Joint Logistic Support Force of the People’s Liberation Army, Lanzhou, China; 38 Cairo University Hospital, Cairo, Egypt; 39 Kasr Alainy Teaching Hospital, Cairo, Egypt; 40 Mataria Teaching Hospital, Cairo, Egypt; 41 Fondation Rothschild, Paris, France; 42 Pitié Salpetriere Hospital, Paris, France; 43 Rennes University Hospital, Rennes, France; 44 Hôpitaux Universitaires de Strasbourg, Strasbourg, France; 45 Children’s Hospital, Dresden Municipal Hospital Teaching Hospital TUD, Dresden, Germany; 46 Medical Center University of Freiburg, Freiburg, Germany; 47 Department of Neurology, Technical University of Munich, Munich, Germany; 48 Mazandaran University of Medical Science, Sari, Islamic Republic of Iran; 49 Institute for Research in Fundamental Sciences (IPM), Tehran, Islamic Republic of Iran; 50 Iranian Research Center for HIV/AIDS, Tehran University of Medical Sciences, Tehran, Iran; 51 Fondazione Poliambulanza Istituto Ospedaliero, Brescia, Italy; 52 University of Brescia, Brescia, Italy; 53 San Gerardo Hospital ASST Monza, University of Milano Bicocca, Monza, Italy; 54 Fondazione Mondino IRCCS, Pavia, Italy; 55 Santa Maria delle Croci Hospital, AUSL Romagna, Ravenna, Italy; 56 Fondazione Policlinico Universitario A. Gemelli IRCCS, Rome, Italy; 57 University Hospital of Rome Tor Vergata, Rome, Italy; 58 Healthcare Trust of the Autonomous Region of Trento, Rovereto, Italy; 59 Città della Salute e della Scienza di Torino, Regina Margherita Children’s Hospital, Turin, Italy; 60 University of Verona, Verona, Italy; 61 Halcyon Healthcare Limited, Nairobi, Kenya; 62 Hôpitaux Robert Schuman, Luxembourg, Luxembourg; 63 Leiden University Medical Center, Leiden, Netherlands; 64 Hospital Regional Docente de Trujillo, Trujillo, Peru; 65 Centro Hospitalar São João, Porto, Portugal; 66 Centro Hospitalar Universitário do Porto, Porto, Portugal; 67 Buyanov Moscow City Hospital, Moscow, Russian Federation; 68 Moscow Research and Clinical Center for Neuropsychiatry and Buyanov Moscow City Hospital, Moscow, Russian Federation; 69 National Neuroscience Institute, Singapore, Singapore; 70 Complejo Hospitalario Universitario de Albacete, Albacete, Spain; 71 Hospital Universitario Virgen de las Nieves, Granada, Spain; 72 University Hospital Sanchinarro, Madrid, Spain; 73 University Hospital Ramón y Cajal, Madrid, Spain; 74 Hospital Virgen de la Salud, Toledo, Spain; 75 Hospital del Río Hortega, Valladolid, Spain; 76 Hopitaux Universitaires de Genève, Geneva, Switzerland; 77 Centre Hospitalier Universitaire Vaudois, Lausanne, Switzerland; 78 Acibadem Mehmet Ali Aydinlar University Medical School, Istanbul, Turkey; 79 Ulster Hospital, Belfast, United Kingdom of Great Britain and Northern Ireland; 80 University of Bristol and North Bristol NHS Trust, Bristol, United Kingdom of Great Britain and Northern Ireland; 81 Gloucestershire Royal Hospital, Gloucester, United Kingdom of Great Britain and Northern Ireland; 82 Great Ormond Street Hospital for Children, London, United Kingdom of Great Britain and Northern Ireland; 83 Imperial College London, London, United Kingdom of Great Britain and Northern Ireland; 84 The National Hospital for Neurology & Neurosurgery, London, United Kingdom of Great Britain and Northern Ireland; 85 University College London, London, United Kingdom of Great Britain and Northern Ireland; 86 University College London Queen Square Institute of Neurology, London, United Kingdom of Great Britain and Northern Ireland; 87 Eastern Pathology Alliance Department of Microbiology, Norfolk and Norwich University Hospitals NHS Foundation Trust, Norwich, United Kingdom of Great Britain and Northern Ireland; 88 Sheffield Teaching Hospitals Trust, Sheffield, United Kingdom of Great Britain and Northern Ireland; 89 Wessex Neurological Centre, Southampton, United Kingdom of Great Britain and Northern Ireland; 90 Emory University School of Medicine, Atlanta, Georgia, United States of America; 91 Massachusetts General Hospital / Harvard Medical School, Boston, Massachusetts, United States of America; 92 Yale New Haven Health Bridgeport Hospital, Bridgeport, Connecticut, United States of America; 93 Rush University Medical Center, Chicago, Illinois, United States of America; 94 University of Arkansas for Medical Sciences, Little Rock, Arkansas, United States of America; 95 Children’s Hospital Los Angeles and Keck School of Medicine at the University of Southern California, Los Angeles, California, United States of America; 96 Ochsner Medical Center, New Orleans, Los Angeles, United States of America; 97 Columbia University Irving Medical Center, New York, New York, United States of America; 98 New York University Grossman School of Medicine, New York, New York, United States of America; 99 Department of Neurology, National Neuroscience Institute, Singapore, Singapore; 100 Institute of Psychiatry, Psychology, and Neuroscience, King’s College London, London, United Kingdom; 101 Division of High-Consequence Pathogens and Pathology, National Center for Emerging and Zoonotic Infectious Diseases, Centers for Disease Control and Prevention, Atlanta, Georgia, United States of America; Fundacao Oswaldo Cruz, BRAZIL

## Abstract

**Background:**

Neurological COVID-19 disease has been reported widely, but published studies often lack information on neurological outcomes and prognostic risk factors. We aimed to describe the spectrum of neurological disease in hospitalised COVID-19 patients; characterise clinical outcomes; and investigate factors associated with a poor outcome.

**Methods:**

We conducted an individual patient data (IPD) meta-analysis of hospitalised patients with neurological COVID-19 disease, using standard case definitions. We invited authors of studies from the first pandemic wave, plus clinicians in the Global COVID-Neuro Network with unpublished data, to contribute. We analysed features associated with poor outcome (moderate to severe disability or death, 3 to 6 on the modified Rankin Scale) using multivariable models.

**Results:**

We included 83 studies (31 unpublished) providing IPD for 1979 patients with COVID-19 and acute new-onset neurological disease. Encephalopathy (978 [49%] patients) and cerebrovascular events (506 [26%]) were the most common diagnoses. Respiratory and systemic symptoms preceded neurological features in 93% of patients; one third developed neurological disease after hospital admission. A poor outcome was more common in patients with cerebrovascular events (76% [95% CI 67–82]), than encephalopathy (54% [42–65]). Intensive care use was high (38% [35–41]) overall, and also greater in the cerebrovascular patients. In the cerebrovascular, but not encephalopathic patients, risk factors for poor outcome included breathlessness on admission and elevated D-dimer. Overall, 30-day mortality was 30% [27–32]. The hazard of death was comparatively lower for patients in the WHO European region.

**Interpretation:**

Neurological COVID-19 disease poses a considerable burden in terms of disease outcomes and use of hospital resources from prolonged intensive care and inpatient admission; preliminary data suggest these may differ according to WHO regions and country income levels. The different risk factors for encephalopathy and stroke suggest different disease mechanisms which may be amenable to intervention, especially in those who develop neurological symptoms after hospital admission.

## Introduction

Since the first reported patients in December 2019, the COVID-19 pandemic has spread globally to cause more than 225 million cases, with over 4.5 million deaths [1]. SARS-CoV-2 virus principally causes respiratory disease, although neurological manifestations were also reported from early in the pandemic, including acute cerebrovascular events, other central and peripheral nervous system disease [2]. There have now been many such reports, but their use of standardised case definitions, detailed clinical and diagnostic evaluation has varied, making comparisons difficult; clinical outcomes and prognostic factors are often not well characterised. Several meta-analyses have also been published [3–8], but given they are based on these original reports, drawing firm conclusions is challenging. In July 2020 we published standardised case definitions for neurological COVID-19 disease [2], including an assessment of the strength of evidence for their association with SARS-CoV-2 infection, which are being used increasingly [2, 9–11]. Using this framework and related data tools, we have now conducted an individual patient data (IPD) meta-analysis of patients with neurological COVID-19 disease from the global first wave. We aimed to firstly describe the spectrum of neurological disease in hospitalised COVID-19 patients using a uniform approach with standardised case definitions; secondly, characterise clinical outcomes; thirdly, investigate factors associated with a poor outcome; and finally, define how frequently acute neurological disease was observed as a proportion of all hospitalised COVID-19 patients. The protocol was registered prospectively on the PROSPERO registry (CRD42020196542).

## Methods

### Search strategy and selection criteria

We searched the following sources for articles published between 1st January 2020 and 3rd July 2020, without language restrictions: PubMed and Scopus; the preprint servers medRxiv and SSRN (Social Science Research Network); and the Brain Infections Global COVID-Neuro Resource and the Journal of Neurology, Neurosurgery and Psychiatry “Neurology and Neuropsychiatry of COVID-19” Blog. We used prespecified search terms modified as needed for each database (S1 Table in S1 Appendix). We applied the following inclusion criteria to studies and then to individual patients: 1) hospitalised patients of any age; 2) diagnosed with COVID-19; and 3) acute onset of neurological symptoms, not explained fully by a pre-existing condition (e.g. progression of chronic neurological disease), with neurological illnesses classified according to our pre-defined syndromes [2], or a defined other neurological or neuropsychiatric diagnosis. Onset of neurological symptoms could have been before or after hospitalisation. We excluded studies that did not report original data, reported patients that were not hospitalised, or gave insufficient information. We selected abstracts and obtained full texts of potentially eligible studies. To compare the results of our IPD meta-analysis with other systematic reviews, meta-analyses and primary studies, including evidence from after the global second wave, we used the same search strategy to obtain articles published up to 30^th^ September 2021.

### Data extraction and processing

We invited authors of published studies, and members of the COVID-Neuro Network of the Brain Infections Global Programme, to participate by providing IPD. Contributors ensured local ethical, regulatory and data sharing agreements were in place. We designed and piloted a standard data collection tool early in the pandemic (S2 Appendix, Section 1 to 3). Details included demographics, comorbidities and pre-admission medications; COVID-19 clinical features, including “typical” COVID-19 symptoms of cough, fever and breathlessness (patient-reported or clinician-assessed), the latter of which was taken as a proxy of COVID-19 severity (oxygen usage and ventilation were not chosen as proxies because access to these varied early in the pandemic, although these data were also collected); investigation results, including PCR (with cycle threshold if positive) and antibody testing for SARS-CoV-2 in blood and cerebrospinal fluid (CSF), with evidence of intrathecal production; COVID-19 disease severity as defined by the World Health Organization (WHO) [12]: neurological features and diagnosis; evidence for association between COVID-19 and neurological disease using pre-defined criteria (S2 Appendix, Section 3.3) [2]; dates of onset of typical and neurological COVID-19 symptoms (including symptoms that were part of the neurological diagnosis), hospital admission and discharge; treatment for COVID-19 (including maximum oxygen or respiratory support) and for neurological disease; admission to critical care, need for invasive ventilation, death, and modified Rankin Scale (mRS) score at discharge. We did not collect data for patients with no neurological disease.

Submitted datasets were cleaned and processed by at least two investigators from a core team of clinical reviewers. This was to harmonise data recording across studies in accordance with pre-defined variable types, descriptions and definitions; complete missing fields where details were available elsewhere in the dataset; and clarify outlying, unexpected or residual missing data with contributors where necessary. If a contributor was unable to harmonise their data with our format, we allowed original study data to be shared with a corresponding data code dictionary; these data were extracted by one reviewer and then checked fully by a second reviewer using an approach standardised through piloting and frequent team discussions.

### Quality assessment

We designed and piloted a bespoke tool to classify study design (S1 Fig in S1 Appendix) and assessed the quality of studies using an appropriate established assessment tool: for case reports and case series we used the Joanna Briggs Institute (JBI) critical appraisal tools [13, 14]; for case-control, cohort and cross-sectional studies, we used the Newcastle-Ottawa Scale (NOS) [15, 16]. Two independent reviewers appraised and assessed the quality of IPD studies, with disagreements resolved by consensus or involvement of a third reviewer.

### Spectrum of neurological disease

Neurological syndromic diagnoses were made by contributors and checked by reviewers using standardised case definitions with levels of diagnostic certainty (S2 Appendix, Section 3.2) [2]. Pre-defined syndromic diagnoses included encephalopathy, encephalitis, meningitis, myelitis, acute disseminated encephalomyelitis (ADEM), and cerebrovascular events (including stroke, vasculitis, and cerebral venous sinus thrombosis). The definitions for encephalopathy (including delirium, coma, subsyndromal delirium and other encephalopathy not classified as delirium or coma, each defined as per the Ten Societies’ recommendations), and for encephalitis were combined for the purpose of the primary subgroup analysis [17]. A secondary analysis was performed for the encephalopathy subgroup excluding patients with encephalitis, who potentially have a different pathophysiological mechanism and so maybe different outcomes. We also included patients with Guillain-Barré syndrome (GBS) and variants, radiculopathy, cranial neuropathy, peripheral neuropathy, myopathy and myositis. Patients with a diagnosis outside our pre-defined criteria were categorised as ‘other neurological presentation’.

### Clinical outcomes

#### Primary outcome

We used the mRS to characterise outcome at hospital discharge, with a mRS score of 3 to 6 (moderate to severe disability or death) defined as a poor outcome.

#### Secondary outcomes

1. Mortality and days from hospital admission to death from any cause.
2. Admission to critical care or receipt of invasive ventilation, referred to hereafter as “need for intensive care”.
3. Length of stay in intensive care.
4. Length of stay in hospital.

### Statistical analysis

We used an ordinal logistic regression model with random effects to account for clustering within studies, and cumulative link function to estimate log cumulative odds of being at or above each mRS category, for all studies providing mRS for patients systematically. We fitted models for patients with any neurological syndrome, and then for the largest subgroups: cerebrovascular events, and encephalopathy. To identify factors associated with a ‘poor outcome’, an mRS of 3 to 6, we first fitted univariate models using a list of covariates. We then adjusted for a predefined subset of these covariates, which we considered important potential confounders, in multivariable logistic regression models (S2 Table in S1 Appendix).

Mortality was analysed using Kaplan–Meier survival curves and marginal Cox regression model using the robust sandwich covariance estimates to account for the clustering of individuals within each study. For outcomes with competing risks (need for intensive care, length of stay in critical care and length of stay in hospital), the cumulative incidence curve for the event of interest in the presence of competing events (death) was estimated, and the subdistribution hazards for clustered data were modelled using the approach described by Zhou et al. [18]. For mortality and need for intensive care (i.e. admission to critical care or receipt of invasive ventilation), a pre-defined set of risk factors were explored in univariate regression models as well as in multivariable models to adjust for confounding factors (S2 Table in S1 Appendix). We used a 5% significance level throughout. In a post-hoc analysis, we compared mortality and need for intensive care estimates between the two largest subgroups, encephalopathy, and cerebrovascular events, using the log-rank test (mortality) and Gray’s test (intensive care). In a post-hoc sensitivity analysis we compared the whole encephalopathy subgroup with a smaller subgroup of patients with encephalopathy that excluded those with a diagnosis of encephalitis.

Finally, we used data from cohort and cross-sectional studies providing verified totals of all patients hospitalised with COVID-19 in their respective centres, to estimate a pooled proportion of COVID-19 patients with neurological disease. Through inspection of study protocols, reports and other information provided by contributors, we ensured that the approaches used to screen and include participants, and to define denominators were similar across studies selected for meta-analysis.

### Funding

The funders had no role in the study design, data collection, data analysis, data interpretation, or writing of this report. The corresponding author had full access to all the data in the study and had final responsibility for the decision to submit it for publication.

## Results and discussion

### Study selection and IPD obtained

**Fig 1:**
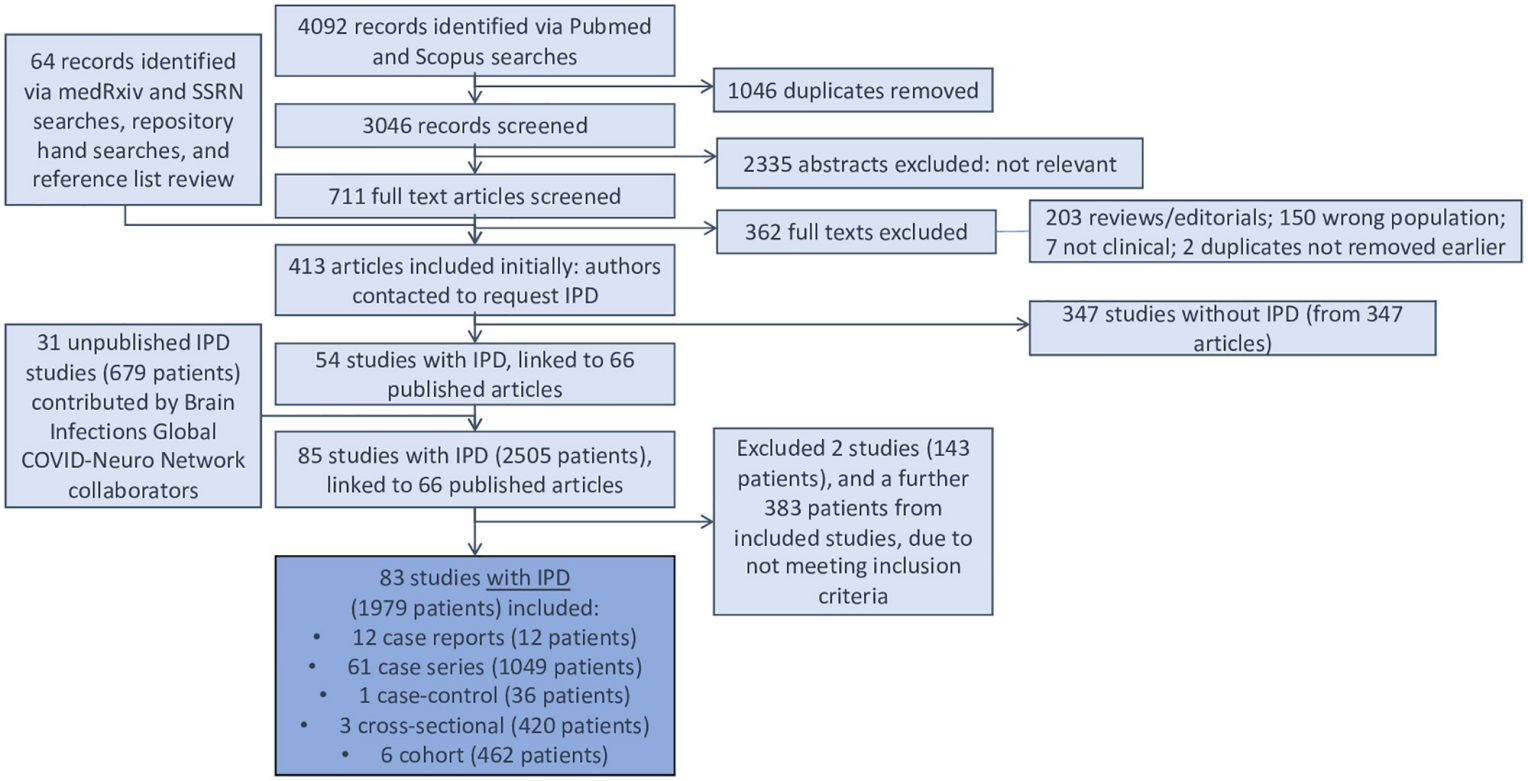
PRISMA flow diagram. IPD = individual patient data.

We identified 4092 records by database searches. After screening these and adding a further 64 records from preprint servers and reference lists, 413 published studies were included (Fig 1). We contacted all study authors, received responses from 128 and received 85 IPD study datasets (2505 patients), comprising 54 published studies, and 31 unpublished studies contributed by Global COVID-Neuro Network collaborators, five of which have now been published. Two studies (143 patients) were excluded as they did not meet inclusion criteria. When inclusion criteria were applied at individual patient-level to 83 studies, 383 patients were excluded, leaving 1979 patients for analyses (Fig 1).

### Characteristics of included studies

**Fig 2:**
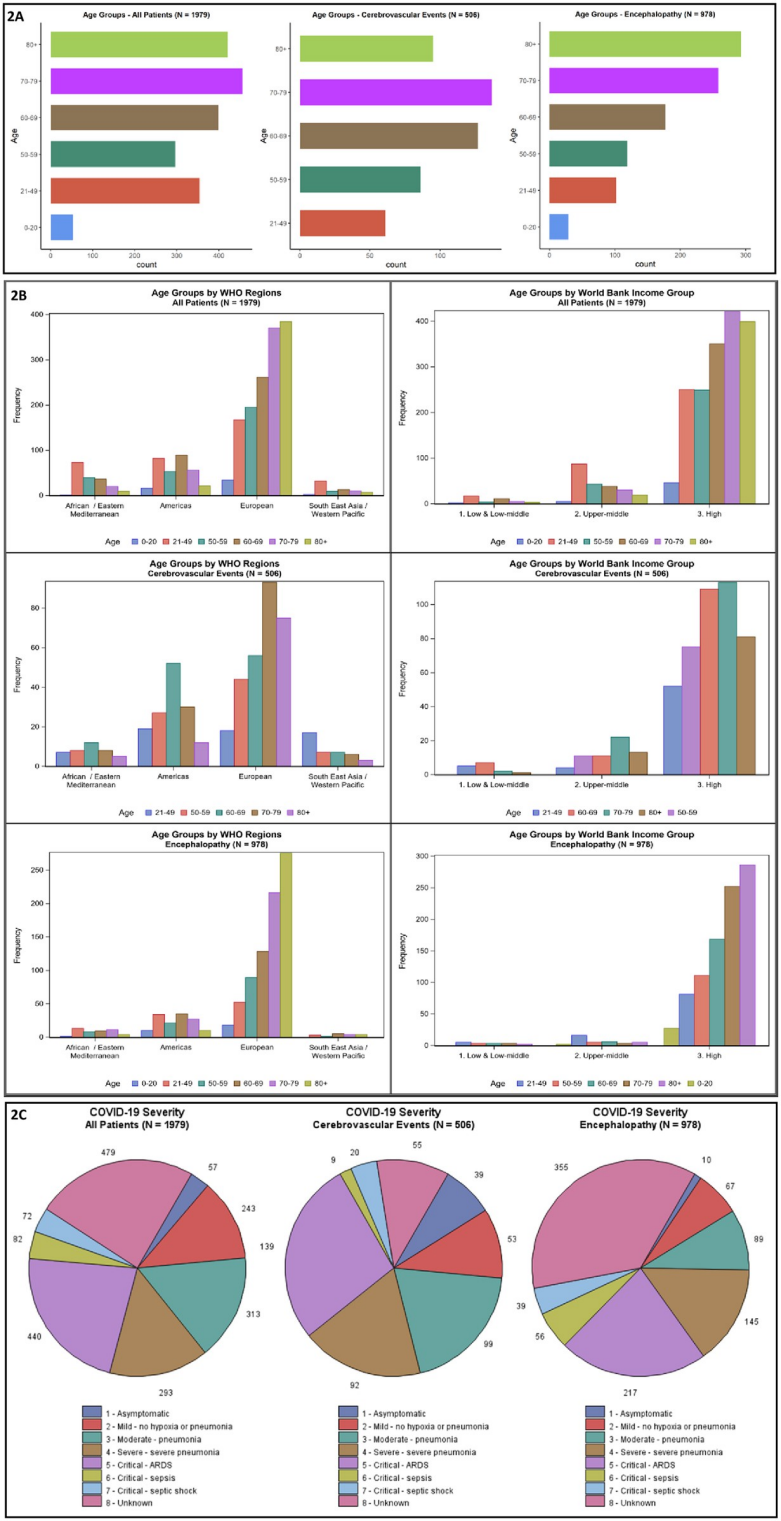
Characteristics of patients in the individual patient data (IPD) database.

**Fig 3:**
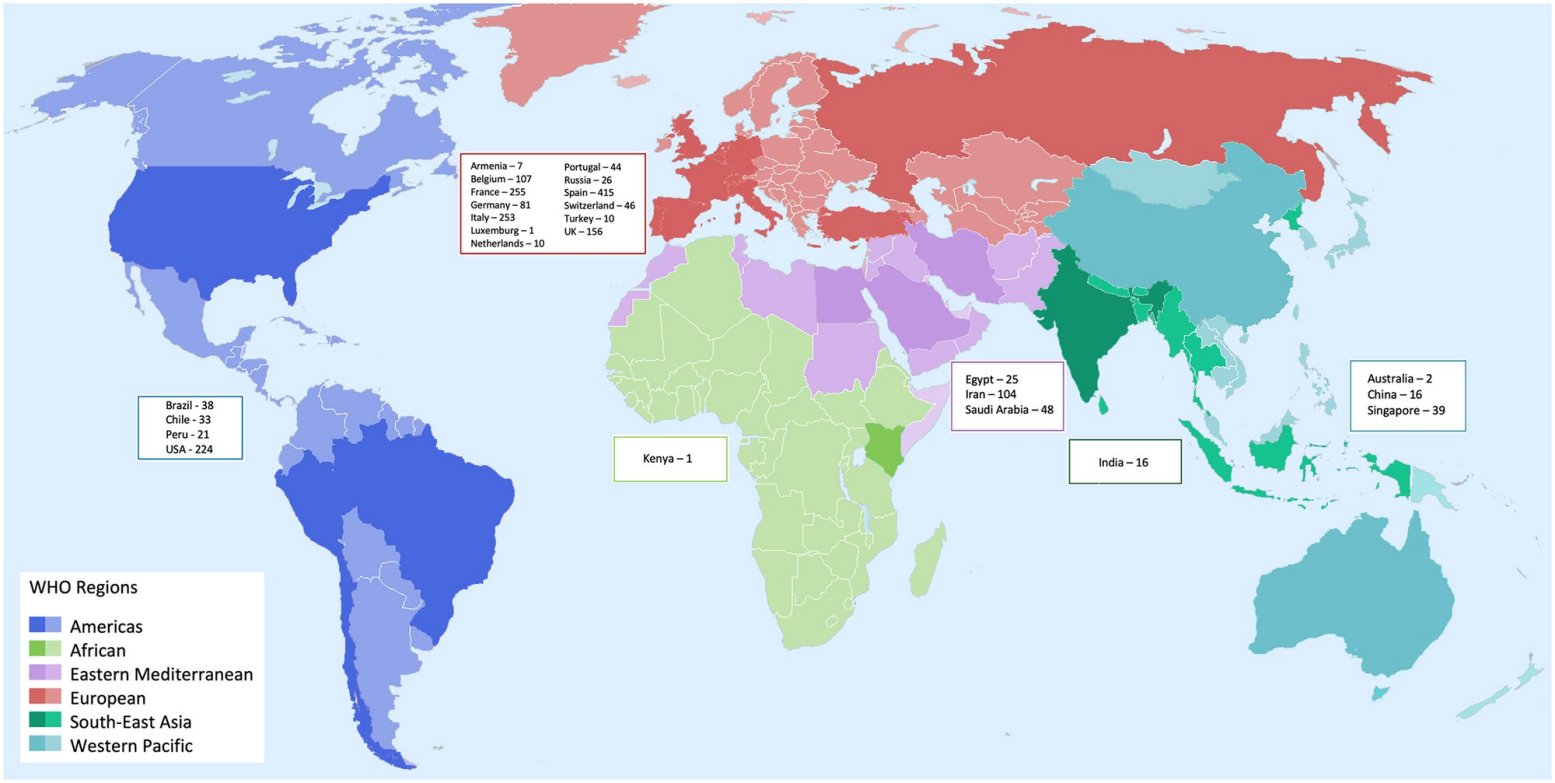
Locations of 1979 patients from 83 studies providing individual patient data (IPD). WHO regions are depicted in different colours. Countries from which we received IPD are depicted in a darker shade. Country names and numbers of patients for which we had IPD are displayed in boxes, grouped according to region.

The make-up of the 83 studies is summarised in S3 Table in S1 Appendix. Case series accounted for the majority of studies (61 [73%] studies, 1049 patients); 26 [31%] studies collected data prospectively; patients were hospitalised across 101 sites; 75 studies included adult patients only (1844 [93%] patients); 1179 [60%] patients were male; and most were aged 60 years and above (Fig 2A). Nineteen (23%) studies reported from low- or middle-income countries (LMICs); 64 (77%) were from high-income countries (HICs). Most studies (53 [64%]) reported from the WHO European region; 16 (19%) from the Americas region; eight (10%) Eastern Mediterranean; three (4%) Western Pacific region; two (2%) Southeast Asian region; and one (1%) African region (S4 Table in S1 Appendix). Fig 2B shows the distribution of age classes by WHO region and World Bank income group. The locations of the included studies are displayed in Fig 3. For 11 of the 83 studies, all patients were on ICU; 17 studies had no patients on ICU; and 55 studies included some patients on ICU. Quality assessments were performed as described above for all studies. Most case reports and case series were of high methodological quality in most domains assessed: 11 of the 12 case reports had an answer of ‘Yes’ for the mandatory domains 1 to 6; and the majority of case series had positive responses for domains 1, 3 and 6–9 of their respective JBI assessment scales. The cohort and cross-sectional studies had lower quality in several domains (for complete assessments see S5 Table in S1 Appendix).

### Spectrum of neurological disease in patients with COVID-19

First, we looked at the spectrum of neurological disease observed in patients with COVID-19 (Table 1). From 83 studies, a total of 1979 patients had a syndromic or specific neurological diagnosis. The most commonly reported syndromes were encephalopathy (978 [49%]), and cerebrovascular events (506 [26%]); other important syndromes included smell or taste disturbance (13%), peripheral neuropathy (6%), GBS (3%) and neuropsychiatric disorders (2.5%). Less than 1% were reported to have each of meningitis, ADEM, myelitis, radiculitis, and myositis. For 1027 patients with both dates available, the median time from the onset of typical COVID-19 symptoms to the onset of neurological symptoms was 5 days (IQR 0–12). For patients with encephalopathy, this was 5 days (IQR 1–10); for cerebrovascular events, 7 days (IQR 0–15); peripheral neuropathy, 13 days (IQR 1–24); and GBS, 12 days (IQR 7–22). Of 807 patients for whom the dates of neurological symptom onset and admission were available, 532 (66%) had neurological symptom onset before the admission to hospital, and 275 (34%) after. This varied by neurological diagnosis: while a similar proportion of patients with encephalopathy (66%) and cerebrovascular events (68%) had neurological features at or before admission, the corresponding proportion was 77% for GBS and 38% for other peripheral neuropathy. The majority of patients (93% [1849/1979] of all patients; 95% [932/978] of the encephalopathy subgroup; 89% [450/506] of the cerebrovascular subgroup) had confirmation of COVID-19 by PCR of a respiratory sample for SARS-CoV-2. Two with myelitis had virus detected in the CSF by PCR; no patient had antibody detected in the CSF. The remaining 7% were either cases confirmed by antibody testing, or clinically probable or suspected cases, based on our prescribed definitions (S3.1 Table in S2 Appendix).

**Table 1:** Frequency of neurological disease subgroups in the studies contributing IPD.

Neurological disease	Studies (N = 83) *n* (%)	Patients (N = 1979) *n* (%)
**Encephalopathy**	**61 (73.5)**	**978 (49.4)**
*Encephalitis*	*37 (44*.*6)*	*92 (4*.*6)*
*Delirium*	*32 (38*.*6)*	*161 (8*.*1)*
*Coma*	*13 (15*.*7)*	*37 (1*.*9)*
*Encephalopathy–other*	*40 (48*.*2)*	*688 (34*.*8)*
*Insufficient information to define subtype*	*0 (0)*	*0 (0)*
**Cerebrovascular event**	**55 (66.3)**	**506 (25.6)**
*Ischaemic*	*45 (54*.*2)*	*308 (15*.*6)*
*Haemorrhagic*	*29 (34*.*9)*	*90 (4*.*5)*
*Vasculitis*	*2 (2*.*4)*	*2 (0*.*1)*
*Cerebrovascular event—other*	*27 (32*.*5)*	*106 (5*.*4)*
*Insufficient information to define subtype*	*0 (0)*	*0 (0)*
**Meningitis**	**9 (10.8)**	**15 (0.8)**
**Acute Disseminated Encephalomyelitis (ADEM)**	**12 (14.5)**	**14 (0.7)**
**Myelitis**	**12 (14.5)**	**13 (0.7)**
**Guillain-Barré syndrome**	**30 (36.1)**	**51 (2.6)**
**Radiculitis**	**2 (2.4)**	**4 (0.2)**
**Peripheral neuropathy**	**24 (28.9)**	**115 (5.8)**
**Myositis**	**2 (2.4)**	**2 (0.1)**
**Other neurological presentation**	**31 (37.3)**	**382 (19.3)**
*Smell or taste disturbance*	*13 (15*.*7)*	*247 (12*.*5)*
*Neuropsychiatric disorder*	*2 (2*.*4)*	*49 (2*.*5)*
*Myopathy*	*5 (6)*	*38 (1*.*9)*
*Autonomic dysfunction*	*4 (4*.*8)*	*27 (1*.*4)*

IPD = individual patient data

1. Individual counts of studies and patients exceed the total numbers in the database, as patients with more than one diagnosis may have been counted more than once within different neurological disease categories.

2. Encephalitis and encephalopathy (including delirium and coma) are pooled together.

3. If patients have more than one neurological disease diagnosis (e.g., encephalitis and myelitis), they are described here. For some diagnoses, these patients may not be captured within the disease categories above.

4. For inclusion, neurological disease had to be acute, i.e. that reached a clinical zenith or plateau less than 28 days from the onset of first neurological symptoms, We included all clinician-defined ‘other’ neurological presentations, including smell or taste disturbance, but we excluded common systemic core complaints of COVID-19 without further qualification, e.g. fatigue, asthenia, myalgia without clinical suspicion of myopathy/myositis, or headache without clinical suspicion of meningitis/cerebrovascular event.

Overall, 887 (45%) of the 1979 patients had severe or critical COVID-19, as per WHO definitions; this proportion was similar in the encephalopathy (47% [457/978]) and cerebrovascular event (51% [260/506]) subgroups (Fig 2C and S6 Table in S1 Appendix). Typical COVID-19 symptoms were present before admission in 747 (93%) of 807 patients.

The 978 (49%) encephalopathy cases were reported across 61 studies, of which 161 (16%) of 978 patients had delirium, 37 (4%) had coma, and 92 (9%) had possible or confirmed encephalitis; 688 (70%) of 978 had features of encephalopathy but did not meet criteria for the aforementioned subtypes and so were described as ‘encephalopathy other’, being not otherwise defined. Of the 506 (26%) patients with a cerebrovascular event, 308 (61%) had an ischaemic stroke, 90 (18%) of 506 haemorrhagic stroke, 2 (0.4%) vasculitis, and 106 (21%) another cerebrovascular event (Table 1). Of these 506 patients, 90% (454 of 506) had neuroimaging that informed diagnosis.

According to our definitions for strength of evidence for an association between infection with SARS-CoV-2 and the development of neurological disease (S2 Appendix, Section 3.3), only two patients met criteria for confirmed association—both had myelitis with a positive CSF PCR test for SARS-CoV-2. Most patients were defined as having a probable association between neurological disease and COVID-19: this applied to 792 (96%) of 826 patients with encephalopathy or encephalitis. The majority of patients with cerebrovascular events for whom this assessment was available were classified as having a possible rather than probable association (362 of 454 [80%]) due to the presence of other pre-defined vascular risk factors. More complete details of the strength of association between neurological disease and infection in patients are provided in S8 Table in S1 Appendix. Four (5%) of the 83 studies included all consecutive patients with neurological COVID-19 disease in a given hospital or region (S10 Table in S1 Appendix). For two of them encephalopathy was the most common presentation, accounting for 50% and 76% of patients, for two cerebrovascular disease predominated (both 64%).

### Outcomes

**Fig 4:**
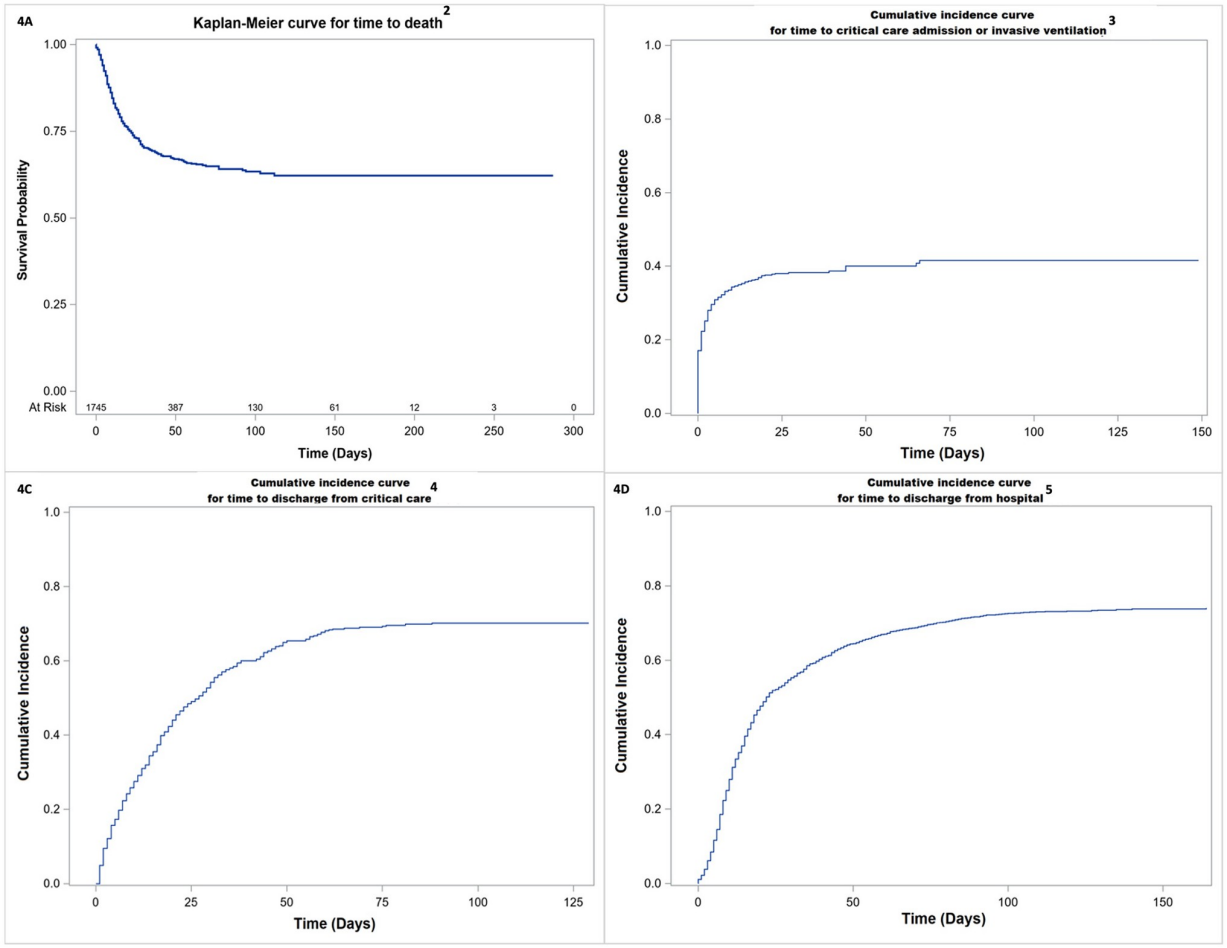
Time-to-event analyses for secondary outcomes for patients with COVID-19 and neurological disease in the IPD database^1^. 1. These figures show results of analyses for the whole IPD database (i.e., patients with any neurological disease diagnosis), and other than for A, the analyses use death as a competing risk. 2. A total of 1745 patients were included in this analysis. Of the 1979, 115 had no dates; 14 patients had no hospital admission date; 9 dead patients had no date of death; 88 alive patients had no discharge date; it was unknown if 8 patients were dead or alive. For time to death, individuals that were alive at discharge or last follow-up were censored. 3. This analysis uses date of hospital admission as day 0. A total of 1428 patients were included in this analysis: 404 patients had no dates; 17 had no hospital admission date; 123 (23 dead; 100 alive) patients had neither the date of admission to critical care or the date of commencement of invasive ventilation; 7 patients only had a hospital admission date, but it was unknown if they were dead or alive. For time to critical care admission, individuals who were alive at discharge or last follow-up and had not been admitted to intensive care were censored. Individuals who died without receiving critical care or invasive ventilation were treated as competing events in a competing risks analysis. 4. This analysis uses date of critical care admission as day 0. A total of 486 patients who were admitted critical care were included in this analysis: 1482 patients had no date of admission to critical care; 5 dead patients had no death date; 5 alive patients had no hospital discharge date; there were no dates for 1 patient. 5. For discharge from critical care, individuals that were alive and not yet discharged at last follow-up were censored. Individuals that died after admission to intensive care were treated as competing events in a competing risks analysis. 10. For length of hospital stay, individuals that were alive and not yet discharged at last follow-up were censored. Individuals that died were treated as competing events in a competing risks analysis.

A poor outcome (moderate to severe disability or death, mRS 3–6) was recorded for 50% (95% CI 41–59) of the 1052 patients in 73 studies reporting mRS systematically, after adjusting for clustering within studies (Table 2). The predicted probability of having no symptoms at discharge (mRS 0) was estimated as 7%. Table 2 shows the probability of each mRS score for 413 patients with encephalopathy and 326 patients with cerebrovascular events, for whom an mRS score was available. There was a higher probability of a poor outcome for cerebrovascular patients (76% [95% CI 67–82]), than encephalopathy patients (54% [95% CI 42–65]). The crude probability of death at 30 days (Fig 4A) was estimated from a Kaplan-Meier analysis as 30% (95% CI 27–32) for all 1745 patients for whom the outcome was available and did not differ significantly for the encephalopathy and cerebrovascular subgroups. For the 1428 patients with adequate data, the crude cumulative incidence of need for intensive care by 30 days was 38% (95% CI 35–41; Fig 4B); this was significantly higher for cerebrovascular patients (47% [95% CI 41–53]; 368 patients) than encephalopathy patients (38% [95% CI 34–42]; 617 patients; Gray’s test p = 0.03). The cumulative incidence of discharge from hospital by 30 days was 55% (95% CI 53–58; Fig 4D) and did not differ significantly between subgroups. Outcomes for the encephalopathy subgroup excluding patients with a diagnosis of encephalitis were all similar to the outcomes of the whole encephalopathy subgroup (S9 Table in S1 Appendix).

**Table 2:** Outcomes—Modified Rankin scale score at discharge, mortality, and need for intensive care—For patients with individual patient data with any neurological disease, and for those with cerebrovascular events and encephalopathy.

	All neurological disease		Encephalopathy subgroup		Cerebrovascular event subgroup	
**Primary outcome**						
**Modified Rankin scale (mRS) score at discharge** ^1^ ^,^ ^2^	**Probability of being at each mRS score** ^3^	**Cumulative probability of being at each mRS score or worse (95% CI)**	**Probability of being at each mRS score** ^3^	**Cumulative probability of being at each mRS score or worse (95% CI)**	**Probability of being at each mRS score** ^3^	**Cumulative probability of being at each mRS score or worse (95% CI)**
**6—Dead**	17%	17% (12–23)	17%	17% (11–25)	33%	33% (25–43)
**5—Severe disability**	6%	23% (17–30)	7%	24% (16–34)	11%	44% (35–54)
**4—Moderately severe disability**	13%	36% (29–45)	13%	37% (27–48)	18%	62% (52–77)
**3—Moderate disability**	13%	50% (41–59)	17%	54% (42–65)	14%	76% (67–82)
**2—Slight disability**	15%	65% (56–73)	15%	69% (58–78)	8%	84% (77–89)
**1—No significant disability despite symptoms**	28%	93% (90–95)	22%	91% (85–94)	11%	95% (91–97)
**0—No symptoms at all**	7%		9%		5%	
**Secondary outcomes**						
**In-hospital mortality at 30 days** ^3^	30% (95% CI 27–32)		38% (95% CI 34–42)		35% (95% CI 30–40)	
**Cumulative incidence of admission to critical care, or invasive mechanical ventilation at 30 days** ^3^	38% (95% CI 35–41)		38% (95% CI 34–42)		47% (95% CI 41–53)	
**Cumulative incidence of discharge from critical care at 30 days**	54% (95% CI 50–59)		53% (95% CI 46–59)		45% (95% CI 37–53)	
**Cumulative incidence of discharge from hospital at 30 days**	55% (95% CI 53–58)		49% (95% CI 46–52)		50% (95% CI 46–55)	

^1^. Full mRS score descriptions:

6—Dead

5—Severe disability; bedridden, incontinent, and requiring constant nursing care and attention

4—Moderately severe disability; unable to walk without assistance and unable to attend to own bodily needs without assistance

3—Moderate disability; requiring some help, but able to walk without assistance

2—Slight disability, unable to carry out all previous activities, but able to look after own affairs without assistance

1—No significant disability despite symptoms

0—No symptoms at all

^2^. The denominators for this analysis were 1052 for all neurological disease, 326 for cerebrovascular disease and 413 for encephalopathy. Patients reported to have both diagnoses were included in the analyses for both cerebrovascular disease and encephalopathy, as well as the ‘all neurological disease’ analysis.

^3^. The confidence intervals presented here do not account for clustering within studies.

### Factors associated with clinical outcomes

On multivariable analysis, after adjusting for potential confounders (S3 Table in S1 Appendix), we identified several factors associated with a poorer outcome (i.e. higher mRS score at hospital discharge) (Table 3). For patients with any neurological diagnosis, these were: age (with the odds ratio [OR] up to 15.3 [95% CI 7.7–30.5] with increasing age); pre-existing dementia (OR 2.6 [1.2–5.7]); breathlessness on admission (OR 1.7 [1.1–2.4]); severely elevated initial blood D-dimer concentration (OR 2.5 [1.4–4.6] for >3000ng/mL vs. <500ng/mL); and corticosteroid use during admission (OR 2.8 [1.8–4.3]). For the encephalopathy subgroup, significant factors on multivariable analysis associated with a poor outcome were: age (OR 5.4 [95% CI 1.4–20.7] for 70–79 years; OR 12.2 [2.8–53.0] for ≥80 years); corticosteroid treatment in hospital (OR 3.6 [1.5–8.9]); anticoagulation in hospital (OR 3.1 [1.3–7.4]); and low initial lymphocyte count (OR 0.4 [0.2–0.9] for normal or high lymphocyte count). For patients with cerebrovascular events, the following were significant: age (OR 3.7 [1.2–11.2] for 60–69 years; OR 4.53 [1.59–12.9] for 70–79 years; OR 6.7 [2.2–20.7] for ≥80 years); elevated D-dimer (OR 2.8 [1.3–6.2] for 500-3000ng/mL; OR 3.5 [1.3–9.7] for ≥3000ng/mL); breathlessness on admission (OR 2.8 [1.4–5.5]); and corticosteroid use during admission (OR 4.8 [1.9–11.9]). For patients with cerebrovascular events, being in the WHO African/Eastern Mediterranean region was associated with a poor outcome relative to the WHO European region (OR 4.4 [1.4–14.4]), whereas being in the Southeast Asia/Western Pacific region was associated with a better outcome relative to the European region (OR 0.2 [0.1–0.9]).

**Table 3:** Univariate and multivariable analyses for variables associated with poor outcome (mRS score 3–6) at discharge for patients with individual patient data with any neurological disease, and for those with cerebrovascular events and encephalopathy^1^^,^^2^^,^^3^.

Variable	Category	All neurological disease: univariate		All neurological disease: multivariable		Encephalopathy: univariate		Encephalopathy: multivariable		Cerebrovascular events: univariate		Cerebrovascular events: multivariable	
		OR (95% CI)	p value	Adjusted OR (95% CI)	p value	OR (95% CI)	p value	Adjusted OR (95% CI)	p value	OR (95% CI)	p value	Adjusted OR (95% CI)	p value
**Age** ^3^	0–20 years	**0.38 (0.15–0.96)**	**<0.0001**	**0.4 (0.08–2.13)**	**<0.0001**	**0.09 (0.02–0.36)**	**<0.0001**	**0.18 (0.02–1.51)**	**0.0009**	No events			
	21–49 years	Reference category				Reference category				Reference category			
	50–59 years	**1.46 (0.99–2.15)**	..	**1.8 (1.01–3.19)**	..	**0.97 (0.43–2.22)**	..	**0.76 (0.21–2.82)**	..	**1.67 (0.75–3.73)**	**0.009**	**2.76 (0.92–8.24)**	**0.027**
	60–69 years	**2.45 (1.66–3.63)**	..	**2.93 (1.61–5.34)**	..	**1.52 (0.71–3.28)**	..	**2.29 (0.59–8.95)**	..	**3.05 (1.37–6.79)**	..	**3.68 (1.21–11.15)**	..
	70–79 years	**4.44 (2.99–6.6)**	..	**5.91 (3.25–10.75)**	..	**3.27 (1.54–6.92)**	..	**5.37 (1.39–20.72)**	..	**3.1 (1.44–6.69)**	..	**4.53 (1.59–12.9)**	..
	≥ 80 years	**8.28 (5.39–12.73)**	..	**15.34 (7.73–30.45)**	..	**4.41 (2.03–9.58)**	..	**12.19 (2.8–53.02)**	..	**4.13 (1.8–9.47)**	..	**6.66 (2.15–20.66)**	..
**Sex at birth**	Male	**1.33 (1.05–1.68)**	**0.02**	1.39 (0.97–1.99)	0.08	1.42 (0.98–2.07)	0.07	1.18 (0.59–2.36)	0.63	1.26 (0.82–1.94)	0.29	1.39 (0.73–2.66)	0.32
**Chronic cardiac disease**	Yes	**1.79 (1.31–2.44)**	**0.0002**	1.05 (0.67–1.66)	0.82	**2.04 (1.27–3.25)**	**0.003**	2.53 (0.87–7.35)	0.09	0.85 (0.5–1.43)	0.53	0.71 (0.35–1.44)	0.35
**Diabetes mellitus**	Yes	**1.52 (1.17–1.97)**	**0.002**	1.15 (0.74–1.8)	0.53	1.29 (0.86–1.93)	0.22	0.73 (0.3–1.77)	0.49	1.4 (0.89–2.19)	0.15	1.8 (0.87–3.71)	0.11
**Obesity**	Yes	1.18 (0.82–1.68)	0.38	1.12 (0.69–1.84)	0.65	1.13 (0.65–1.94)	0.67	1.21 (0.5–2.89)	0.67	1.78 (0.87–3.64)	0.12	1.5 (0.59–3.81)	0.39
**Chronic neurological disease** ^4^	Yes	1.27 (0.81–1.99)	0.3	1.25 (0.67–2.35)	0.48	1.51 (0.83–2.75)	0.17	1.44 (0.53–3.87)	0.47	1.1 (0.38–3.21)	0.86	0.53 (0.13–2.13)	0.37
**Dementia**	Yes	**3.39 (2.11–5.47)**	**<0.0001**	**2.59 (1.18–5.72)**	**0.02**	**2.42 (1.31–4.48)**	**0.005**	3.28 (0.96–11.17)	0.06	1.35 (0.59–3.09)	0.47	1.73 (0.59–5.01)	0.32
**Anti-platelet use prior to admission**	Yes	**1.85 (1.29–2.64)**	**<0.0001**	1.19 (0.73–1.94)	0.49	**2.24 (1.19–4.22)**	**0.013**	1.19 (0.47–3.03)	0.72	1.15 (0.66–2.03)	0.62	1.27 (0.6–2.66)	0.53
**Breathlessness on admission to hospital**	Yes	**1.91 (1.49–2.45)**	**< .0001**	**1.66 (1.14–2.4)**	**0.008**	**2.14 (1.41–3.27)**	**0.0005**	1.3 (0.63–2.68)	0.48	**2.85 (1.82–4.46)**	**<0.0001**	**2.81 (1.43–5.52)**	**0.003**
**Cough on admission to hospital**	Present	0.95 (0.74–1.22)	0.69	1.12 (0.77–1.64)	0.56	0.92 (0.61–1.41)	0.71	1.45 (0.68–3.1)	0.34	1.4 (0.88–2.23)	0.16	0.87 (0.44–1.72)	0.68
**Initial blood lymphocyte count**	≥1 x10^9/L	**0.57 (0.44–0.73)**	**<0.0001**	0.78 (0.54–1.12)	0.18	0.7 (0.47–1.02)	0.07	**0.44 (0.21–0.91)**	**0.03**	**0.37 (0.24–0.6)**	**<0.0001**	0.7 (0.36–1.35)	0.29
**Initial serum CRP concentration**	≥10 mg/L	**1.78 (1.37–2.33)**	**<0.0001**	1.18 (0.78–1.79)	0.43	**2.88 (1.78–4.68)**	**<0.0001**	2.11 (0.85–5.22)	0.11	1.49 (0.97–2.31)	0.07	1.22 (0.63–2.36)	0.56
**Initial blood D-dimer concentration** ^3^	<500 ng/mL	Reference category				Reference category				Reference category			
	500–3000 ng/mL	**1.38 (1.04–1.82)**	**<0.0001**	**1.34 (0.88–2.04)**	**0.01**	1.05 (0.66–1.65)	0.25	1.15 (0.48–2.74)	0.58	**1.54 (0.92–2.55)**	**0.01**	**2.84 (1.3–6.17)**	**0.01**
	≥3000 ng/mL	**2.77 (1.87–4.11)**	..	**2.51 (1.37–4.59)**	..	1.59 (0.9–2.81)	..	1.84 (0.57–5.92)	..	**2.67 (1.42–5.02)**	..	**3.52 (1.28–9.72)**	..
**Anticoagulation use during admission**	Yes	**1.51 (1.13–2.02)**	**0.006**	1.29 (0.85–1.97)	0.23	**2.57 (1.53–4.33)**	**0.0005**	**3.11 (1.3–7.44)**	**0.01**	1.2 (0.76–1.91)	0.44	1.31 (0.69–2.51)	0.41
**Corticosteroid use during admission**	Yes	**1.52 (1.12–2.07)**	**0.007**	**2.78 (1.81–4.27)**	**<0.0001**	**1.87 (1.1–3.19)**	**0.02**	**3.64 (1.49–8.85)**	**0.006**	1.71 (0.93–3.13)	0.09	**4.81 (1.94–11.92)**	**0.0009**
**World Bank income group**^3^^,^ ^5^	Low- or Lower-middle income	1.06 (0.21–5.31)	0.94	2.23 (0.42–11.93)	0.47	0.8 (0.1–6.46)	0.42	2.15 (0.15–30.28)	0.85	1.83 (0.35–9.69)	0.77	3.99 (0.54–29.49)	0.28
	Upper-middle income	0.87 (0.37–2.03)	..	1.55 (0.61–3.96)	..	0.49 (0.17–1.41)	..	1 (0.24–4.27)	..	1.07 (0.44–2.59)	..	1.87 (0.6–5.88)	..
	High-income	Reference category				Reference category				Reference category			
**World Health Organization region**^3^^,^ ^5^	African / Eastern Mediterranean	1.28 (0.45–3.66)	0.64	2.03 (0.6–6.85)	0.56	0.88 (0.23–3.46)	0.88	1.01 (0.14–7.35)	0.37	**2.55 (1.14–5.71)**	**<0.0001**	**4.42 (1.36–14.42)**	**0.007**
	Americas	1.17 (0.48–2.83)	..	1.67 (0.56–4.97)	..	1.12 (0.38–3.33)	..	2.96 (0.6–14.66)	..	**1.3 (0.64–2.66)**	..	**1 (0.28–3.63)**	..
	South East Asia / Western Pacific	0.41 (0.08–2.05)	..	0.81 (0.15–4.32)	..	2.01 (0.33–12.32)	..	4.85 (0.4–58.46)	..	**0.12 (0.04–0.34)**	..	**0.22 (0.05–0.89)**	..
	European	Reference category				Reference category				Reference category			

OR = odds ratio, CRP = C-reactive protein

^1^. Statistically significant variables are in bold (alpha of 0.05).

^2^. Categories containing fewer than 10 patients were not included in the analysis model for that variable.

^3^. Variables with multiple categories have pre-defined reference categories against which other categories are compared.

^4^. Excluding dementia but including cerebrovascular disease.

^5^. For the purposes of analysis data from the WHO Southeast Asia and Western Pacific Regions were pooled, as were data from the Eastern Mediterranean and African regions; similarly, data from World Bank low and low-middle income countries were pooled. This was pre-defined in the statistical analysis plan, prior to performing the analyses.

Hazard of death among patients with any neurological disease was found be associated with age, dementia, breathlessness at presentation, corticosteroid use in hospital, WHO Region (higher for all regions compared with Europe) and World Bank income group (higher for low- and lower-middle income countries), following adjustment for confounders in multivariable models (Table 4). For patients with encephalopathy, age, dementia, corticosteroid use in hospital, WHO region and World Bank income group were statistically significant after adjustment for confounders. Adjusted multivariable models for the cerebrovascular patients found a significant association with increased hazard of death and low lymphocyte count, corticosteroid treatment, and WHO region, whereas anticoagulant use in hospital was protective.

**Table 4:** Univariate and multivariable cox regression analyses for variables associated with death for patients with individual patient data with any neurological disease, and for those with cerebrovascular events and encephalopathy^1^^,^^2^^,^^3^.

Variable	Category	All neurological disease: univariate		All neurological disease: multivariable		Encephalopathy: univariate		Encephalopathy: multivariable		Cerebrovascular events: univariate		Cerebrovascular events: multivariable	
		HR (95% CI)	p value	Adjusted HR (95% CI)	p value	HR (95% CI)	p value	Adjusted HR (95% CI)	p value	HR (95% CI)	p value	Adjusted HR (95% CI)	p value
**Age** ^3^	0–20 years	**0.18 (0.02–1.72)**	**<0.0001**	**0.25 (0.03–2.55)**	**0.0004**	**No events**		**No events**		**No events**		**No events**	
	21–49 years	Reference category				Reference category				Reference category			
	50–59 years	**1.05 (0.69–1.61)**	..	**1.22 (0.59–2.53)**	..	**0.48 (0.24–0.97)**	**< .0001**	**0.49 (0.15–1.54)**	**< .0001**	**1.13 (0.52–2.47)**	**0.01**	1.66 (0.6–4.6)	0.35
	60–69 years	**2.37 (1.4–4)**	..	**1.94 (1.1–3.42)**	..	**1.14 (0.66–1.99)**	..	**1.03 (0.39–2.71)**	..	**2.29 (1.13–4.68)**	..	1.69 (0.63–4.58)	..
	70–79 years	**3.94 (2.31–6.7)**	..	**2.41 (1.22–4.77)**	..	**2.18 (1.45–3.28)**	..	**1.28 (0.52–3.14)**	..	**2.21 (1.07–4.59)**	..	1.63 (0.57–4.61)	..
	≥ 80 years	**5.55 (3.22–9.59)**	..	**3.85 (1.87–7.92)**	..	**3.02 (1.88–4.87)**	..	**2.17 (0.78–6.1)**	..	**2.69 (1.25–5.76)**	..	3.04 (0.93–9.89)	..
**Sex at birth**	Male	1.03 (0.82–1.29)	0.8	1.24 (0.8–1.92)	0.33	1.03 (0.81–1.3)	0.83	0.58 (1.72–1)	1	1.12 (0.69–1.81)	0.65	1.2 (0.57–2.52)	0.64
**Chronic cardiac disease**	Yes	**1.79 (1.4–2.29)**	**< .0001**	0.9 (0.57–1.42)	0.64	**1.74 (1.35–2.25)**	**<0.0001**	0.95 (0.42–2.18)	0.91	1.31 (0.87–1.97)	0.19	0.88 (0.47–1.66)	0.69
**Diabetes mellitus**	Yes	**1.39 (1.15–1.69)**	**0.001**	1.45 (0.95–2.23)	0.09	**1.26 (1.07–1.48)**	**0.005**	1.37 (0.81–2.32)	0.24	1.16 (0.85–1.58)	0.34	1.58 (0.74–3.38)	0.24
**Obesity**	Yes	1.23 (0.85–1.79)	0.27	1.23 (0.89–1.71)	0.22	1.09 (0.61–1.95)	0.77	1.42 (0.88–2.31)	0.15	1.18 (0.8–1.73)	0.41	0.79 (0.4–1.58)	0.51
**Chronic neurological disease** ^4^	Yes	1.03 (0.62–1.72)	0.91	**0.57 (0.37–0.89)**	**0.01**	0.92 (0.53–1.6)	0.78	0.54 (0.17–1.69)	0.29	1.27 (0.7–2.3)	0.43	0.84 (0.42–1.71)	0.64
**Dementia**	Yes	**2.57 (1.71–3.86)**	**<0.0001**	**1.95 (1.2–3.16)**	**0.007**	**2.17 (1.4–3.37)**	**0.001**	**1.91 (1.01–3.62)**	**0.046**	1.19 (0.72–1.97)	0.49	1.52 (0.85–2.73)	0.16
**Anti-platelet use prior to admission**	Yes	**1.83 (1.29–2.59)**	**0.001**	1.2 (0.82–1.73)	0.35	**1.67 (1.13–2.47)**	**0.01**	1.4 (0.7–2.8)	0.34	1.11 (0.63–1.96)	0.71	1.03 (0.56–1.89)	0.92
**Breathlessness on admission to hospital**	Yes	**1.88 (1.22–2.88)**	**0.004**	**2.19 (1.33–3.6)**	**0.002**	1.71 (0.93–3.14)	0.09	2.2 (0.98–4.92)	0.06	**2.07 (1.28–3.34)**	**0.003**	1.87 (0.93–3.77)	0.08
**Cough on admission to hospital**	Present	0.82 (0.64–1.04)	0.1	0.82 (0.57–1.19)	0.3	0.91 (0.67–1.24)	0.54	1.58 (0.83–2.99)	0.16	1.03 (0.73–1.45)	0.89	0.76 (0.42–1.38)	0.37
**Initial blood lymphocyte count**	≥1 x10^9/L	0.86 (0.53–1.41)	0.55	0.868 (0.641–1.176)	0.36	0.9 (0.55–1.48)	0.68	0.68 (0.32–1.45)	0.32	**0.61 (0.37–1)**	**0.049**	**0.62 (0.41–0.93)**	**0.02**
**Initial serum CRP concentration**	≥10 mg/L	1.42 (0.71–2.84)	0.32	1.11 (0.68–1.82)	0.67	1.28 (0.56–2.95)	0.56	0.99 (0.35–2.86)	0.99	1.34 (0.82–2.19)	0.24	1.13 (0.67–1.91)	0.65
**Initial blood D-dimer concentration** ^3^	<500 ng/mL	Reference category				Reference category				Reference category			
	500–3000 ng/mL	**1.11 (0.65–1.9)**	**0.001**	0.98 (0.59–1.6)	0.26	**0.96 (0.53–1.74)**	**0.009**	0.63 (0.29–1.39)	0.52	1.11 (0.65–1.9)	0.29	1.36 (0.74–2.51)	0.21
	≥3000 ng/mL	**1.92 (1.1–3.35)**	..	1.47 (0.85–2.52)	..	**1.46 (0.75–2.82)**	..	0.91 (0.57–1.47)	..	1.4 (0.87–2.25)	..	0.91 (0.44–1.88)	..
**Anticoagulation use during admission**	Yes	1.16 (0.83–1.61)	0.38	0.89 (0.56–1.42)	0.63	1.2 (0.83–1.74)	0.34	1.03 (0.62–1.73)	0.9	0.88 (0.57–1.36)	0.56	**0.56 (0.32–0.96)**	**0.04**
**Corticosteroid use during admission**	Yes	1.32 (0.94–1.87)	0.11	**2.09 (1.37–3.17)**	**0.001**	1.25 (0.87–1.81)	0.23	**3.11 (1.61–6)**	**0.0007**	1.44 (0.88–2.35)	0.15	**2.02 (1.08–3.77)**	**0.03**
**World Bank income group**^3^^,^ ^5^	Low- or Lower-middle income	1.34 (0.67–2.67)	0.19	**3.05 (1.3–7.13)**	**0.03**	2.39 (0.69–8.25)	0.1	**7.2 (1.5–34.63)**	**0.045**	1.39 (0.27–7.17)	0.86	1.79 (0.4–7.99)	0.58
	Upper-middle income	0.44 (0.13–1.49)	..	1.79 (0.75–4.29)	..	0.29 (0.06–1.5)	..	1.13 (0.19–6.54)	..	1.2 (0.52–2.77)	..	1.67 (0.58–4.86)	..
	High-income	Reference category				Reference category				Reference category			
**World Health Organization region**^3^^,^ ^5^	African / Eastern Mediterranean	0.77 (0.22–2.76)	0.26	**4.39 (2.48–7.78)**	**<0.0001**	1.1 (0.58–2.09)	0.48	**8 (2.52–25.4)**	**<0.0001**	**2.1 (1.19–3.72)**	**<0.0001**	**3.03 (1.39–6.61)**	**0.009**
	Americas	1.09 (0.53–2.24)	..	**3.16 (1.7–5.9)**	..	0.73 (0.33–1.6)	..	**2.64 (1.51–4.61)**	..	**1.3 (0.75–2.26)**	..	**1.52 (0.51–4.55)**	..
	South East Asia / Western Pacific	0.54 (0.24–1.22)	..	**2.19 (1.06–4.52)**	..	1.37 (0.42–4.46)	..	**8.49 (2.37–30.48)**	..	**0.4 (0.22–0.71)**	..	**0.67 (0.32–1.41)**	..
	European	Reference category				Reference category				Reference category			

CI = confidence interval; HR = hazard ratio. CRP = C-reactive protein

^1^. Statistically significant variables are in bold (alpha of 0.05).

^2^. Categories containing fewer than 10 patients were not included in the analysis model for that variable.

^3^. Variables with multiple categories have pre-defined reference categories against which other categories are compared.

^4^. Excluding dementia but including cerebrovascular disease

^5^. For the purposes of analysis data from the WHO Southeast Asia and Western Pacific Regions were pooled, as were data from the Eastern Mediterranean and African regions; similarly, data from World Bank low and low-middle income countries were pooled. This was pre-defined in the statistical analysis plan, prior to performing the analyses.

Multivariable regression analysis found a statistically significant association with requiring intensive care for male sex, breathlessness at presentation, pre-existing dementia or diabetes, increased CRP, elevated D-dimer, anticoagulant use, corticosteroid use, WHO region and World Bank income group (Table 5). After fitting models for the encephalopathy subgroup, a statistically significant increased hazard of requiring intensive care was found for age (≥80 years), obesity, dementia, breathlessness, elevated CRP and D-dimer, corticosteroid use, WHO region, and World Bank income group. In the cerebrovascular event subgroup, pre-existing cardiac disease or dementia, corticosteroid treatment in hospital, income group and WHO region were significant after adjustment for confounders.

**Table 5:** Univariate and multivariable regression models (accounting for clustering and competing risk of death) for variables associated with intensive care for patients with individual patient data with any neurological disease, and for those with cerebrovascular events and encephalopathy^1^^,^^2^^,^^3^.

Variable	Category	All neurological disease: univariate		All neurological disease: multivariable		Encephalopathy: univariate		Encephalopathy: multivariable		Cerebrovascular events: univariate		Cerebrovascular events: multivariable	
		HR (95% CI)	p value	Adjusted HR (95% CI)	p value	HR (95% CI)	p value	Adjusted HR (95% CI)	p value	HR (95% CI)	p value	Adjusted HR (95% CI)	p value
**Age** ^3^	0–20 years	**2.13 (0.76–5.97)**	**<0.0001**	2.16 (0.85–5.49)	0.053	**1.56 (0.62–3.93)**	**<0.0001**	**1.33 (0.44–4.03)**	**0.0008**	**No events**		**No events**	
	21–49 years	Reference category				Reference category				Reference category			
	50–59 years	**1.52 (1.21–1.92)**	..	1.31 (0.87–1.97)	..	**1.38 (0.9–2.11)**	..	**1.29 (0.64–2.62)**	..	1.02 (0.62–1.67)	0.053	0.82 (0.49–1.4)	0.36
	60–69 years	**1.5 (1.06–2.11)**	..	1.41 (0.93–2.13)	..	**0.94 (0.62–1.43)**	..	**0.97 (0.49–1.89)**	..	1.07 (0.66–1.76)	..	0.89 (0.48–1.68)	..
	70–79 years	**0.83 (0.51–1.35)**	..	1.19 (0.76–1.86)	..	**0.4 (0.23–0.7)**	..	**0.69 (0.31–1.55)**	..	0.78 (0.46–1.31)	..	0.88 (0.48–1.6)	..
	≥ 80 years	**0.24 (0.1–0.54)**	..	0.55 (0.29–1.07)	..	**0.11 (0.05–0.25)**	..	**0.26 (0.1–0.66)**	..	0.27 (0.11–0.67)	..	0.37 (0.13–1.03)	..
**Sex at birth**	Male	**1.28 (1.04–1.58)**	**0.02**	**1.31 (1.03–1.66)**	**0.03**	1.24 (0.92–1.67)	0.16	1.36 (0.94–1.98)	0.11	1.22 (0.86–1.74)	0.27	1.1 (0.73–1.66)	0.66
**Chronic cardiac disease**	Yes	**0.62 (0.41–0.95)**	**0.03**	0.73 (0.52–1.04)	0.08	**0.45 (0.26–0.78)**	**0.004**	1.03 (0.71–1.49)	0.87	0.72 (0.39–1.34)	0.3	**0.58 (0.35–0.98)**	**0.04**
**Diabetes mellitus**	Yes	**1.24 (0.99–1.56)**	**0.07**	**1.43 (1.07–1.9)**	**0.01**	1.16 (0.86–1.56)	0.34	0.9 (0.63–1.28)	0.55	1.05 (0.78–1.43)	0.74	1.41 (0.99–2.02)	0.06
**Obesity**	Yes	**2.42 (1.79–3.27)**	**<0.0001**	1.31 (0.97–1.77)	0.08	**3.05 (1.9–4.89)**	**<0.0001**	**1.67 (1.07–2.61)**	**0.03**	**1.72 (1.03–2.87)**	**0.04**	0.9 (0.63–1.27)	0.55
**Chronic neurological disease** ^4^	Yes	0.89 (0.52–1.54)	0.69	0.91 (0.64–1.29)	0.59	1 (0.51–1.97)	1	0.94 (0.62–1.44)	0.79	1.12 (0.6–2.1)	0.72	1.47 (0.76–2.84)	0.25
**Dementia**	Yes	0.78 (0.36–1.68)	0.52	**2.33 (1.58–3.43)**	**<0.0001**	0.53 (0.22–1.29)	0.16	**1.68 (1.08–2.6)**	**0.02**	1.38 (0.84–2.25)	0.2	**2.05 (1.31–3.2)**	**0.002**
**Anti-platelet use prior to admission**	Yes	1.09 (0.76–1.55)	0.65	1.09 (0.81–1.46)	0.58	0.89 (0.57–1.4)	0.62	0.96 (0.66–1.41)	0.85	0.93 (0.66–1.32)	0.69	1.08 (0.7–1.66)	0.73
**Breathlessness on admission to hospital**	Yes	**1.93 (1.33–2.79)**	**0.001**	**1.88 (1.25–2.83)**	**0.002**	**1.95 (1.11–3.44)**	**0.02**	**2.26 (1.34–3.81)**	**0.002**	**1.68 (1.11–2.55)**	**0.01**	1.46 (0.85–2.49)	0.17
**Cough on admission to hospital**	Present	**1.55 (1.09–2.21)**	**0.02**	0.92 (0.63–1.34)	0.66	**1.51 (1.02–2.24)**	**0.04**	0.93 (0.62–1.39)	0.71	1.55 (0.9–2.65)	0.11	1.05 (0.65–1.69)	0.85
**Initial blood lymphocyte count**	≥1 x10^9/L	1.06 (0.72–1.56)	0.76	1.07 (0.81–1.39)	0.65	1.17 (0.67–2.04)	0.57	0.93 (0.64–1.36)	0.71	1.2 (0.85–1.69)	0.29	1.07 (0.81–1.42)	0.64
**Initial serum CRP concentration**	≥10 mg/L	1.02 (0.59–1.75)	0.95	**1.56 (1.05–2.31)**	**0.03**	1.1 (0.48–2.51)	0.82	2.18 (1.09–4.38)	**0.03**	**1.33 (0.87–2.05)**	0.19	1.42 (0.97–2.06)	0.07
**Initial blood D-dimer concentration** ^3^	<500 ng/mL	Reference category				Reference category				Reference category			
	500–3000 ng/mL	1.29 (0.76–2.19)	0.13	**1.91 (1.29–2.84)**	**0.0004**	1.4 (0.7–2.81)	0.6	**2.12 (1.31–3.43)**	**0.003**	**1.18 (0.72–1.94)**	**0.04**	1.39 (0.8–2.42)	0.5
	≥3000 ng/mL	1.88 (0.98–3.59)	..	**2.34 (1.53–3.59)**	..	1.65 (0.62–4.39)	..	**2.33 (1.42–3.82)**	..	**2 (1.14–3.49)**	..	1.51 (0.73–3.1)	..
**Anticoagulation use during admission**	Yes	**1.5 (1.05–2.16)**	**0.03**	**1.59 (1.02–2.47)**	**0.04**	1.38 (0.92–2.05)	0.12	1.53 (0.78–3.02)	0.22	1.33 (0.89–1.97)	0.16	1.18 (0.75–1.87)	0.48
**Corticosteroid use during admission**	Yes	**2.48 (1.67–3.67)**	**<0.0001**	**1.87 (1.32–2.63)**	**0.0004**	**2.79 (1.55–5.02)**	**0.0006**	**1.76 (1.08–2.84)**	**0.02**	**1.83 (1.21–2.77)**	**0.004**	**1.73 (1.16–2.57)**	**0.007**
**World Bank income group**^3^^,^ ^5^	Low- or Lower-middle income	**2.82 (1.85–4.31)**	**<0.0001**	**1.9 (1.19–3.05)**	**0.03**	**4.1 (2.4–6.99)**	**<0.0001**	**2.15 (1.21–3.79)**	**0.02**	**3.55 (1.98–6.35)**	**<0.0001**	**2.56 (1.6–4.1)**	**<0.0001**
	Upper-middle income	**0.96 (0.4–2.27)**	..	**1.59 (0.73–3.45)**	..	**1.36 (0.51–3.64)**	..	**0.86 (0.3–2.49)**	..	**2.35 (0.94–5.87)**	..	**3.43 (1.86–6.3)**	..
	High-income	Reference category				Reference category				Reference category			
**World Health Organization region**^3^^,^ ^5^	African / Eastern Mediterranean	1.45 (0.55–3.8)	0.69	**2.2 (1.26–3.85)**	**0.03**	**2.1 (1.03–4.28)**	**0.007**	**1.03 (0.41–2.57)**	**0.0003**	**3.29 (1.55–6.99)**	**0.0008**	**2.58 (1.24–5.36)**	**0.002**
	Americas	1.56 (0.65–3.73)	..	**0.94 (0.4–2.19)**	..	**2.38 (1–5.66)**	..	**0.88 (0.38–2.01)**	..	**1.41 (0.47–4.24)**	..	**0.76 (0.27–2.13)**	..
	South East Asia / Western Pacific	1.39 (0.76–2.56)	..	**1.42 (0.9–2.24)**	..	**3.76 (1.75–8.11)**	..	**2.8 (1.7–4.61)**	..	**1.08 (0.41–2.87)**	..	**0.72 (0.35–1.48)**	..
	European	Reference category				Reference category				Reference category			

CI = confidence interval; HR = hazard ratio. CRP = C-reactive protein

^1^. Variables found to be significant at a p-value cut-off of 0.05 are in bold type.

^2^. Categories containing fewer than 10 patients were not included in the analysis model for that variable.

^3^. Variables with multiple categories have pre-defined reference categories against which other categories are compared.

^4^. Excluding dementia but including cerebrovascular disease.

^5^. For the purposes of analysis data from the WHO Southeast Asia and Western Pacific Regions were pooled, as were data from the Eastern Mediterranean and African regions; similarly, data from World Bank low and low-middle income countries were pooled. This was pre-defined in the statistical analysis plan, prior to performing the analyses.

### Proportion of patients with neurological COVID-19 disease

**Fig 5:**
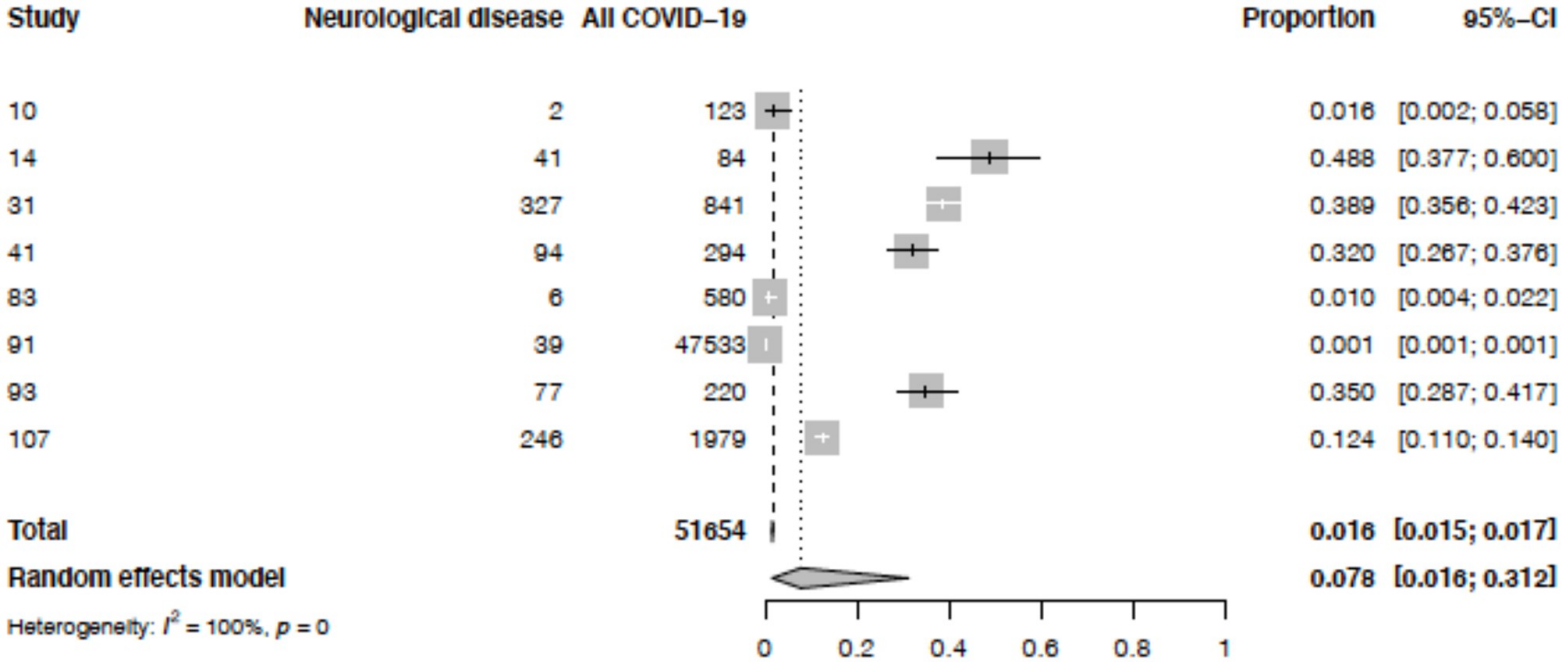
Pooled proportions of all patients hospitalised with COVID-19 reported to have acute new-onset neurological disease. Neurological disease = number of patients with neurological COVID-19 disease. All COVID-19 = number of patients with all COVID-19 disease hospitalised in the same centre over the same time period.

Eight of the 83 studies included the total number of neurological and other hospitalised COVID-19 patients, admitted over a specified time period, in a comparable way which could be analysed. Five were case series. Fig 5 illustrates that, overall, 7.8% (95% CI 1.6–31.2) of hospitalised COVID-19 patients had neurological disease. The I^2^ statistic showed a high degree of statistical heterogeneity among studies (100%). The studies contributing data are summarised in S11 Table in S1 Appendix. When one study, which included patients admitted to community isolation facilities as well as to hospitals, was excluded in a sensitivity analysis (S2 Fig in S1 Appendix), the pooled percentage of hospitalised COVID-19 patients who had neurological disease was 14.7% (95% CI 4.7–37.8; I^2^ 98%).

## Discussion

Since the onset of the COVID-19 pandemic, there has been a plethora of studies reporting associated neurological disease, initially without the use of standardised case definitions, and often still without detailed clinical and diagnostic evaluation, investigation of prognostic markers or clinical outcomes. To some extent, this reflected the difficulties of studying a new highly infectious disease that was swamping health services, plus the desire to publish important information quickly [2]. Several meta-analyses have now also been published based on these original reports [3–8], but they may not accurately capture the true clinical picture, given the limitations of the original data.

In July 2020, we published standardised case definitions for neurological COVID-19 disease [2], which included assessment of the strength of evidence for an association, and have been modified and are being adopted by the Global Covid-19 Neuro Research Coalition and the WHO [19]. We therefore decided to perform an IPD meta-analysis of published and unpublished data from patients admitted to hospital during the first wave of the pandemic, using these case definitions and a standardised data collection tool. To date, no other published meta-analysis has included IPD for multiple pre-defined neurological diagnoses, though one large analysis combined data from two cohorts of patients with neurological COVID-19 and a third with or without neurological disease [20]. We received data on 1979 patients supplied from 83 studies (including 31 that were originally unpublished). Most previous systematic reviews described symptoms and diagnoses, with some estimating the proportion of COVID-19 patients that develop neurological disease. Here, we concentrated on detailed descriptions of the neurological diseases, their outcomes, and risk factors for a poor prognosis. This latter is especially important for neurologists and other hospital specialists who care for such patients. We also compared WHO regions and World Bank income groups, to initiate thinking about differences in outcomes across the global community.

The neurological syndromes seen most commonly were encephalopathy (49%), including encephalitis, coma, and delirium, and cerebrovascular events (26%), principally ischaemic stroke (Table 1). There were also many patients with smell or taste disturbance (19%), and some with peripheral neuropathy (6%), GBS (3%) and neuropsychiatric disorders (2.5%). The cerebrovascular case definitions worked relatively well in terms of classifying patients. The encephalopathy definitions worked less well with 35% of patients being classed as “encephalopathy other” because they did not fit into the main categories of delirium, coma and encephalitis; although there has been considerable debate on encephalopathy case definitions among the neurology, geriatric, and psychiatric community [10, 21, 22] these results suggest clinicians may be unfamiliar with the definitions, or they may need further revision. Encephalopathy may be precipitated by many different factors, in the context of different diseases, and the spectrum of clinical features of this syndrome can make succinct classification a challenge. Assessment of patients with suspected delirium in our study was performed by clinicians, guided by the variables included in our data collection form; existing tools such as CAM-ICU, 4AT or AMT-4 could also be used to quantify neurocognitive features in more detail. In anticipation of other factors that can impact on conscious level, cognition, and behaviour, we also collected data on brain imaging and use of hypnotic and anxiolytic agents during hospital admission. For most encephalitis patients the aetiological link to SARS-CoV-2 was classed as “probable” or “possible”, because no virus was detected in their CSF. This is in contrast to herpes simplex virus encephalitis where virus is frequently detected, and there is marked inflammatory change on brain imaging or autopsy. Over a year into the pandemic, we now know that virus detection in the CSF is extremely rare, and the case definitions should probably be refined to reflect this, perhaps following the approach for enterovirus 71, which also causes severe brain disease with inflammatory changes despite virus rarely being detected in the CSF [23, 24]. Of note, two myelitis patients had virus detected in CSF; we did not have the PCR cycle threshold values from this testing, but given the implications of true confirmed viral myelitis on management, this finding should be rigorously confirmed by by performing PCR for SARS-CoV-2 on CSF of myelitis patients who have concurrent or recent COVID-19, or who present during a pandemic wave.

In previous systematic reviews encephalopathy and cerebrovascular disease were the most commonly reported neurological presentations, though which of these was most important varied [25–29]. This likely reflects differences in study populations and case definitions. Even for the four studies in our analysis that recruited consecutive neurological patients, and where we could apply strict case definitions to the individual patient data, two studies had a predominance of patients with cerebrovascular events and the other two had a majority of patients with encephalopathy (S10 Table in S1 Appendix). These differences may stem from varying approaches to screening for neurological symptoms and inclusion of hospitalised COVID-19 patients. In our database overall, we found encephalopathy was reported for about half the patients, and stroke for about a quarter. This is similar to one of the larger prospective series of 606 unselected neurological patients in New York, which found encephalopathy in 50% and stroke in 14% [30]. Another recent study combining COVID-19 and neurological disease patient registries reported encephalopathy in 49%, and stroke in 6% [20].

Approximately half of the 1052 patients with neurological COVID-19 disease and a mRS score available had a poor outcome on discharge from hospital, as determined by a mRS score of 3–6 (moderate to severe disability or death; Table 2); the proportion was higher in those with cerebrovascular events (76%) than encephalopathy (54%), and this was largely accounted for by those that died (33% versus 17%). Our findings highlight the degree of disability experienced by patients with COVID-19 and neurological disease; a recent report of hospitalised UK patients in the UK ISARIC-4C study found that functional outcomes are worse in those with neurological complications compared to those with other severe but non-neurological complications of COVID-19 [31]. In another study, the adjusted odds ratio of in-hospital death was 5.99 (95%CI: 4.33–8.28) for those with any neurological signs and/or syndromes compared to those without, though the odds ratio was greater for encephalopathy than stroke [20].

The mRS was devised for stroke and although it is not particularly reliable for brain injuries that result in cognitive disability [32], it is still widely used in this group. Future studies of neurological disability should use a more generic outcome score such as the Glasgow Outcome Scale which is equally simple to administer and may better capture the impact of neuropsychiatric manifestations [33]. Clinicians were not blinded to the patients neurological condition at the time of mRS assessment, but the outcome measure was clearly defined. Variable time to discharge (at which point mRS was calculated) may have affected our results, as we did not account for this in our multivariable models.

Overall, 30% of the 1745 neurological patients with outcome information available had died by 30 days, which is higher than the mortality of around 25% reported by meta-analyses of all hospitalised COVID-19 patients from North America, Europe, and China [34, 35]. Our higher mortality rate is in keeping with the report of the ISARIC-4C study, which found patients with neurological COVID-19 complications (specifically meningitis, encephalitis, seizure, or stroke) had an increased hazard of mortality [31]. A previous systematic review of patients with neurological disease reported a lower mortality of 10%, but this study may have included non-hospitalised patients with neurological symptoms such as headache [3]. Nearly 40% of our patients needed intensive care (higher for those with a cerebrovascular event than for the encephalopathic patients). No previous systematic reviews of neurological COVID-19 patients have meta-analysed for these outcomes, though in the ISARIC-4C study, 22% of those with neurological complications were admitted to critical care compared with 14% of patients overall [31]. Approximately half of our neurological COVID-19 patients who required intensive care support still needed this at 30 days. Whilst we did not have a control group, this is considerably longer than what has been reported in published studies for all COVID-19 patients in intensive care: 12 days in one large UK cohort study [36], and 8 days in a meta-analysis [37]. 45% of our patients were still in hospital at 30 days. While previous systematic reviews of neurological COVID-19 do not report this, our estimate appears longer than studies reporting on all hospitalised COVID-19 patients: median length of stay was 12 days for one study of 1321 patients in France [38], and 8 days for 2005 patients in Germany [39] Collectively our results on the need for and duration of intensive care, length of hospital stay, and patient outcomes underscore the significant burden of neurological COVID-19 disease on health care resources, compared with COVID-19 disease as a whole. Post-acute COVID-19 neurological symptoms and outcomes are also an emerging and important issue, though longer-term data were not available for us to investigate this [40].

We found age, and markers of disease severity including breathlessness and elevated D-dimer were associated with a poor outcome among all patients with neurological disease (Table 3). These same factors were also important for the subgroup with cerebrovascular events, but not those with encephalopathy. This is in keeping with an earlier report from the ISARIC-4C study indicating that patients presenting with encephalopathy, with or without typical COVID-19 symptoms, had a higher mortality [41]. Low initial lymphocyte count was associated with poor outcome in our encephalopathy patients, as has been shown in a meta-analysis of over 10,000 patients with COVID-19 [42]. Other biomarkers shown to be important in COVID-19 generally, such as neutrophil and platelet counts, were not available consistently for our patients [43].

Corticosteroid use in hospital was associated with a worse outcome in all neurological patients, as well as the cerebrovascular and encephalopathic subgroups (Table 3). This is likely to be because clinicians were more inclined to use corticosteroids in these patients with severe disease. Anticoagulation use in hospital was also associated with a worse outcome in the encephalopathic patients, but intriguingly it was associated with a lower hazard of death in those with cerebrovascular events (Table 4), suggesting it may be beneficial in these patients. Further work is needed to understand the role of anticoagulation in COVID-19 patients with stroke. While these variables might have been susceptible to immortal time bias, this is unlikely to have influenced the results significantly, as these drugs are usually started at admission.

Although international comparison was not the primary aim of our study, we could begin to explore differences in outcomes between different WHO regions, and World Bank income groups. We found that compared with neurological COVID-19 patients in the WHO European region, those in other regions had a higher hazard ratio for death (Table 4); the hazard ratio was also higher for patients from low- and lower-middle-income countries compared to high-income countries (HICs), though with wide confidence intervals, reflecting fewer patients in the lower-income category. Differences in mRS scores were also seen across WHO regions (Table 3), but only in patients with cerebrovascular disease and again with wide confidence intervals. Although these are only preliminary data, these differences may reflect broader public health approaches and capacities in different countries [44]. Further larger-scale studies including LMICs are needed to investigate these potential findings.

Although we applied standard case definitions (S2 Appendix, Section 3) and eligibility criteria to our IPD database, the original studies or case series had been conducted using different protocols, and many were small and did not capture all patients with neurological disease, potentially leading to selection bias. However, 47% of our patients were from cross-sectional and cohort studies, and the case series scored highly across several quality assessment domains. Our novel approach of capturing unpublished data through the Global Covid-Neuro Network meant we included patients, especially from LMICs, that would not otherwise have ever been included in a publication, thus improving accessibility and equity (S4 Table in S1 Appendix). Despite this, just 6 (7%) of 83 studies providing 42 (2%) of the total 1979 patients were from low- or lower-middle income countries. Only 2% of our patients were children. This may reflect some degree of residual selection bias, despite not relying upon published literature. Finally, despite using multivariable analyses with pre-defined exposures and confounders, associations do not equate to causation; determining these would require further research.

## Conclusions

We have shown that encephalopathy and stroke are the most commonly reported neurological manifestations of COVID-19, with the latter group having a worse outcome, as judged by the mRS. Nearly 40% of patients needed intensive care, and the burden in terms of prolonged intensive care and hospital stay was higher than for other hospitalised COVID-19 patients. Markers of disease severity such as breathlessness and elevated D-dimer were associated with poor outcome in the cerebrovascular event, but not the encephalopathic, patients, suggesting different disease mechanisms. For one third of the patients, the neurological symptoms started after hospital admission, providing a potential window for intervention if risk factors and neurological disease mechanisms were better understood. Prospective case-control studies across multiple WHO regions are needed to better understand the factors leading to neurological COVID-19 and point to potential interventions.

## Supporting information

S1 Checklist PRISMA-IPD checklist. (PDF)

S1 Appendix Author list, supplementary figures and tables. (DOCX)

S2 Appendix Data extraction tool and case definitions. (PDF)
