# Supplementary material for: Prognostic indicators and outcomes of hospitalised COVID-19 patients with neurological disease: An individual patient data meta-analysis
Source: PLoS One. 2022 Jun 2;17(6):e0263595. doi: 10.1371/journal.pone.0263595 (PMC9162376; doi:10.1371/journal.pone.0263595)
Supplement: S1 Appendix — (DOCX) [file pone.0263595.s002.docx]

# Supplementary appendix S1 – author list, supplementary figures and tables

**Prognostic indicators and outcomes of hospitalised COVID-19 patients with neurological disease: a systematic review and individual patient data meta-analysis**

**Contents**

**Section 1:** Author list and contributions

**Section 2:** Supplementary Figures

**Section 3:** Supplementary Tables

**Section 1: Authors & contributions of the Brain Infections Global COVID-Neuro Network Study Group**

**Writing Committee**

Bhagteshwar Singh*, Suzannah Lant*, Sofia Cividini, Jonathan W S Cattrall, Lynsey C Goodwin, Laura Benjamin, Benedict D Michael, Ayaz Khawaja, Aline de Moura Brasil Matos, Walid Alkeridy, Andrea Pilotto, Durjoy Lahiri, Thirugnanam Umapathi, Timothy R Nicholson, James J Sejvar, Eva Maria Hodel, Catrin Tudur Smith^, Tom Solomon^

* equal contribution

^ joint senior authors

**University of Liverpool Statistical Analysis Team**

Sofia Cividini, Catrin Tudur Smith

**COVID-Neuro Global Scientific Steering Committee**

Rebecca Rawlinson, Sithembinkosi Mhlanga, Eva Maria Hodel, Bhagteshwar Singh, Rafael Freitas de Oliveira França, Vasanthapuram Ravi, Priscilla Rupali, Jennifer Cornick, Tom Solomon, Carlos Brito, Maria Lúcia Brito Ferreira, Netravathi M, Anita Desai, Ajith Sivadasan, Tamara Phiri, Victor Mwapasa, Neil French, Laura Benjamin, Girvan Burnside, Dan Wootton, Michael Griffiths, Ava Easton

**Brain Infections Global Group Investigators** (those not listed in COVID-Neuro Global Scientific Steering Committee above)

Camila Pimentel, Christopher Parry, Trudie Lang, Richard Lilford, Santosh K Chaturvedi, BN Gangadhar, G Gururaj, Pradeep BS, Priya Thomas, Asha Abraham, Nicola Desmond, Stephen Ray, Macpherson Mallewa, Linda Nyondo Mipando

**Data Management Team**

Jonathan W S Cattrall, Sofia Cividini, Lynsey C Goodwin, Eva Maria Hodel, Suzannah Lant, Evelyn Lόpez, Brendan F Sargent, Bhagteshwar Singh, Tom Solomon, Anushri Somasundaran, Arina Tamborska, Catrin Tudur Smith, Glynn Webb, Komal Younas

**Systematic Review Team**

Yaqub Al Sami, Heavenna Babu, Tristan Banks, Jonathan W S Cattrall, Francesco Cavallieri, Matthew Cohen, Emma Davies, Shalley Dhar, Anna Fajardo Modol, Hamzah Farooq, Lynsey C Goodwin, Jeffrey Harte, Samuel Hey, Albert Joseph, Dileep Karthikappallil, Daniel Kassahun, Suzannah Lant, Gareth Lipunga, Evelyn Lόpez, Rachel Mason, Thomas Minton, Gabrielle Mond, Aline de Moura Brasil Matos, Joseph Poxon, Sophie Rabas, Brendan F Sargent, Bhagteshwar Singh, Anushri Somasundaran, Germander Soothill, Arina Tamborska, Glynn Webb, Komal Younas, Marialuisa Zedde

**Liverpool Brain Infections Group Investigators**

Sylviane Defres, Lance Turtle, Fiona McGill, Benedict D Michael, Rachel Kneen

**Data Contributors** (in alphabetical order of country name)

**Yerevan State Medical University named after Mkhitar Heratsi, Neuroscience Laboratory, Cobrain Center, Yerevan, Armenia** Konstantin Yenkoyan

**St Vincent's Hospital, Sydney, Australia** Bruce Brew, Erika Contini, Lucette Cysique, Xin Zhang

**Saint-Luc University Hospital, Brussels, Belgium** Pietro Maggi, Vincent van Pesch

**Université de Mons, Mons, Belgium** Jérome Lechien, Sven Saussez

**AZ Glorieux, Ronse, Belgium** Alex Heyse

**Hospital da Restauração, Recife, Brazil** Maria Lúcia Brito Ferreira

**Hospital Federal dos Servidores do Estado, Rio de Janeiro, Brazil** Cristiane N Soares

**Instituto de Medicina Tropical, University of Sao Paulo, Sao Paulo, Brazil** Aline de Moura Brasil Matos on behalf of the NeuroCovBR study group

**Instituto de Infectologia Emilio Ribas, Sao Paulo, Brazil,** Aline de Moura Brasil Matos on behalf of the NeuroCovBR study group

**Hospital Geral de Fortaleza, Ceara, Brazil**, Aline de Moura Brasil Matos on behalf of the NeuroCovBR study group

**Hospital Dr. Sótero del Río, Santiago, Chile** Isabel Elicer, Laura Eugenín-von Bernhardi.

**Universidad de Chile - Hospital Barros Luco Trudeau, Santiago, Chile** Waleng Ñancupil Reyes

**The 940^th^ Hospital of Joint Logistic Support Force of the People's Liberation Army, Lanzhou, China** Rong Yin

**Cairo University Hospital, Cairo, Egypt** Mohammed Atef Azab

**Kasr Alainy Teaching Hospital, Cairo, Egypt** Foad Abd-Allah

**Mataria Teaching Hospital, Cairo, Egypt** Ahmed Elkady

**University Hospital Nantes, Nantes, France** Gaëlle Balloy

**Fondation Rothschild, Paris, France** Simon Escalard

**Pitié Salpetriere Hospital, Paris, France** Jean-Christophe Corvol, Cécile Delorme

**Rennes University Hospital, Rennes, France** Paul Petitgas, Pierre Tattevin

**Hôpitaux Universitaires de Strasbourg, Strasbourg, France** Kévin Bigaut

**Children's Hospital, Dresden Municipal Hospital Teaching Hospital TUD, Dresden, Germany** Norbert Lorenz

**Medical Center University of Freiburg, Freiburg, Germany** Daniel Hornuss, Jonas Hosp, Siegbert Rieg, Dirk Wagner

**Department of Neurology, Technical University of Munich, Munich, Germany** Benjamin Knier, Paul Lingor, Andrea Sylvia Winkler

**Bangur Institute of Neurosciences, Institute of Post-Graduate Medical Education and Research, Kolkata, India** Durjoy Lahiri

**Dayanand Medical College and Hospital, Ludhiana, India** Gagandeep Singh

**Mazandaran University of Medical Science, Sari, Islamic Republic of Iran** Narges Karimi, Athena Sharifi-Razavi

**Institute for Research in Fundamental Sciences (IPM), Tehran, Islamic Republic of Iran** Shima T. Moein

**Iranian Research Center for HIV/AIDS, Tehran University of Medical Sciences, Tehran, Islamic Republic of Iran** SeyedAhmad SeyedAlinaghi, Saeidreza JamaliMoghadamSiahkali

**Fondazione Poliambulanza Istituto Ospedaliero, Brescia, Italy** Mauro Morassi

**University of Brescia, Brescia, Italy** Alessandro Padovani, Andrea Pilotto, Marcello Giunta, Ilenia Libri

**San Gerardo Hospital ASST Monza, University of Milano Bicocca, Monza, Italy** Simone Beretta

**Fondazione Mondino IRCCS, Pavia, Italy** Sabrina Ravaglia

**Santa Maria delle Croci Hospital - AUSL Romagna, Ravenna, Italy**Matteo Foschi

**Fondazione Policlinico Universitario A. Gemelli IRCCS, Rome, Italy** Paolo Calabresi, Guido Primiano, Serenella Servidei

**University Hospital of Rome Tor Vergata, Rome, Italy** Nicola Biagio Mercuri, Claudio Liguori, Mariangela Pierantozzi, Loredana Sarmati

**Healthcare Trust of the Autonomous Region of Trento, Rovereto, Italy** Federica Boso

**Città della Salute e della Scienza di Torino, Regina Margherita Children's Hospital, Turin, Italy** Silvia Garazzino

**University of Verona, Verona, Italy** Sara Mariotto

**Halcyon Healthcare Limited, Nairobi, Kenya** Kimani N Patrick

**Hôpitaux Robert Schuman, Luxembourg, Luxembourg** Oana Costache, Alexander Pincherle

**Leiden University Medical Center, Leiden, Netherlands** Frederikus A. Klok

**Hospital Regional Docente de Trujillo, Trujillo, Peru** Roger Meza

**Centro Hospitalar São João, Porto, Portugal** Verónica Cabreira

**Centro Hospitalar Universitário do Porto, Porto, Portugal** Sofia R. Valdoleiros, Vanessa Oliveira

**Buyanov Moscow City Hospital, Moscow, Russian Federation** Igor Kaimovsky

**Moscow Research and Clinical Center for Neuropsychiatry and Buyanov Moscow City Hospital, Moscow, Russian Federation** Alla Guekht

**King Saud University, Riyadh, Saudi Arabia** Walid Alkeridy

**National Neuroscience Institute, Singapore, Singapore** Thirugnanam Umapathi, Jasmine Koh

**Complejo Hospitalario Universitario de Albacete, Albacete, Spain** Eva Fernández Díaz

**Hospital Universitario Virgen de las Nieves, Granada, Spain** José María Barrios-López

**University Hospital Sanchinarro, Madrid, Spain** Cristina Guijarro-Castro

**University Hospital Ramón y Cajal, Madrid, Spain** Álvaro Beltrán-Corbellini, Javier Martínez-Poles

**Hospital Mútua de Terrassa, Terrassa, Spain** Javier Sotoca

**Hospital Virgen de la Salud, Toledo, Spain** Alba Maria Diezma-Martin, M. Isabel Morales-Casado

**Hospital del Río Hortega, Valladolid, Spain** Sergio García García

**Hopitaux Universitaires de Genève, Geneva, Switzerland** Gautier Breville, Matteo Coen, Marjolaine Uginet

**Centre hospitalier universitaire Vaudois, Lausanne, Switzerland** Raphaël Bernard-Valnet, Renaud Du Pasquier

**Acibadem Mehmet Ali Aydinlar University Medical School, Istanbul, Turkey** Yildiz Kaya

**Ulster Hospital, Belfast, United Kingdom of Great Britain and Northern Ireland** Loay H. Abdelnour

**University of Bristol and North Bristol NHS Trust, Bristol, United Kingdom of Great Britain and Northern Ireland** Claire Rice

**Gloucestershire Royal Hospital, Gloucester, United Kingdom of Great Britain and Northern Ireland** Hamish Morrison

**Liverpool University Hospitals NHS Foundation Trust, Liverpool,** **United Kingdom of Great Britain and Northern Ireland** Sylviane Defres

**The Walton Centre NHS Foundation Trust, Liverpool, United Kingdom of Great Britain and Northern Ireland** Saif Huda

**Great Ormond Street Hospital for Children, London, United Kingdom of Great Britain and Northern Ireland** Noelle Enright, Jane Hassell

**Imperial College London, London, United Kingdom of Great Britain and Northern Ireland** Lucio D'Anna

**King's College Hospital, London, United Kingdom of Great Britain and Northern Ireland** Matthew Benger, Laszlo Sztriha, Owain Williams

**The National Hospital for Neurology & Neurosurgery, London, United Kingdom of Great Britain and Northern Ireland** Eamon Raith

**University College London, London, United Kingdom of Great Britain and Northern Ireland** Krishna Chinthapalli, Guru Kumar, Ross Nortley, Ross Paterson

**University College London Queen Square Institute of Neurology, London, United Kingdom of Great Britain and Northern Ireland** Laura Benjamin, Arvind Chandratheva, David Werring

**Norfolk and Norwich University Hospitals NHS Foundation Trust, Norwich, United Kingdom of Great Britain and Northern Ireland** Samir Dervisevic, Ekkehart Staufenberg

**Sheffield Teaching Hospitals Trust, Sheffield, United Kingdom of Great Britain and Northern Ireland** Kirsty Harkness

**Wessex Neurological Centre, Southampton, United Kingdom of Great Britain and Northern Ireland** Ashwin Pinto

**Emory University School of Medicine, Atlanta, United States of America** Dinesh Jillella

**Massachusetts General Hospital / Harvard Medical School, Boston, United States of America** Scott Beach

**Yale New Haven Health Bridgeport Hospital, Bridgeport, United States of America** Kulothungan Gunasekaran

**Rush University Medical Center, Chicago, United States of America** Pranusha Pinna, Ivan Rocha Ferreira Da Silva

**Wayne State University, Detroit, United States of America** Ayaz Khawaja

**University of Arkansas for Medical Sciences, Little Rock, United States of America** Krishna Nalleballe

**Children's Hospital Los Angeles and Keck School of Medicine at the University of Southern California, Los Angeles, United States of America** Jonathan Santoro

**Ochsner Medical Center, New Orleans, United States of America** Tyler Scullen, Lora Kahn

**Columbia University Irving Medical Center, New York, United States of America** Carla Y Kim, Kiran Thakur

**New York University Grossman School of Medicine, New York, United States of America** Rajan Jain

**Supplementary Acknowledgements List (*not* part of the Brain Infections Global COVID-Neuro Network)**

**Pitié Salpetriere Hospital, Paris, France** Marion Houot, Charlotte Rosso, Stéphanie Carvalho, Thomas Nedelec, Redwan Maatoug, Victor Pitron, Salimata Gassama, Sara Sambin, Stéphanie Bombois, Bastien Herlin, Gaëlle Ouvrard, Gaëlle Bruneteau, Adèle Hesters, Ana Zenovia Gales, Bruno Millet, Foudil Lamari, Stéphane Lehericy, Vincent Navarro, Benjamin Rohaut, Sophie Demeret, Thierry Maisonobe, Marion Yger, Bertrand Degos, Louise-Laure Mariani, Christophe Bouche, Nathalie Dzierzynski, Bruno Oquendo, Flora Ketz, An-Hung Nguyen, Aurélie Kas, Jean-Yves Delattre

**Rush University Medical Center,** **Chicago, United States of America** Parneet Grewal, Julianne Hall, Tachira Tavarez, Rajeev Garg, Sajona John, James Conners, Rima Dafer

**Instituto de Infectologia Emilio Ribas, Sao Paulo, Brazil,** Augusto Cesar Penalva de Oliveira

**Hospital Geral de Fortaleza, Ceara, Brazil**, Fernanda Martins Maia Carvalho

**Section 2: Supplementary Figures**

# **Figure S1: Bespoke tool made to align decision making regarding study design label, and which quality assessment tool to use**

JBI: Joanna Briggs Institute; NOS: Newcastle-Ottawa Scale (this included use of the NOS-cross sectional tool; Herzog R, et al. Are healthcare workers' intentions to vaccinate related to their knowledge, beliefs and attitudes? A systematic review. *BMC Public Health* 2013; **13**: 154).

**Figure S2: Forest plot showing sensitivity analysis of meta-analysis to calculate pooled proportion of patients hospitalised with COVID-19 who have acute new-onset neurological disease, excluding outlying study from Singapore**


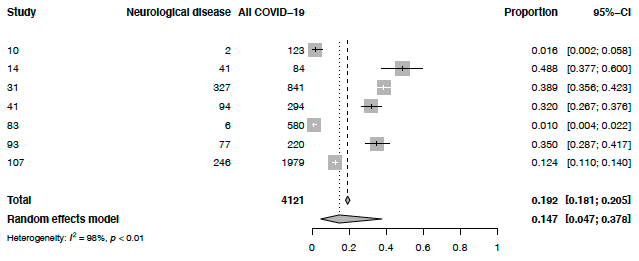


Neurological disease: number of patients with neurological COVID-19 disease

All COVID-19: number of patients with all COVID-19 disease hospitalised in the same centre over the same time period.

Excluded outlying study (compared to figure 5 in the main paper): study 91

# **Section 3: Supplementary Tables**

# **Table S1: Search strategy**

| # | DESCRIPTION | PUBMED SEARCH TERMS | SCOPUS SEARCH TERMS |
| --- | --- | --- | --- |
| 1 | COVID-19 or SARS-CoV-2 | coronavir*[Title/Abstract] OR coronovirus*[Title/Abstract] OR "corona virus"[Title/Abstract] OR "virus corona"[Title/Abstract] OR "corono virus"[Title/Abstract] OR "virus corono"[Title/Abstract] OR hcov*[Title/Abstract] OR "covid-19"[Title/Abstract] OR covid19*[Title/Abstract] OR "covid 19"[Title/Abstract] OR “2019-nCoV“[Title/Abstract] OR cv19*[Title/Abstract] OR "cv-19"[Title/Abstract] OR "cv 19"[Title/Abstract] OR "n-cov"[Title/Abstract] OR ncov*[Title/Abstract] OR "sars-cov-2"[Title/Abstract] OR (wuhan*[Title/Abstract] AND (virus[Title/Abstract] OR viruses[Title/Abstract] OR viral[Title/Abstract] OR coronav*[Title/Abstract])) OR (novel[Title/Abstract] AND coronav*[Title/Abstract]) OR "2019-nCoV"[Title/Abstract] OR 2019nCoV[Title/Abstract] OR nCoV2019[Title/Abstract] OR "nCoV-2019"[Title/Abstract] OR "COVID-19"[Title/Abstract] OR COVID19[Title/Abstract] OR "HCoV-19"[Title/Abstract] OR HCoV19[Title/Abstract] OR CoV[Title/Abstract] OR "2019 novel*"[Title/Abstract] OR Ncov[Title/Abstract] OR "n-cov"[Title/Abstract] OR "SARS-CoV-2"[Title/Abstract] OR "SARSCoV-2"[Title/Abstract] OR SARSCoV2[Title/Abstract] OR SARSCoV2[Title/Abstract] OR SARSCov19[Title/Abstract] OR "SARS-Cov-19"[Title/Abstract] OR Coronavirus[MeSH Terms] | ( title-abs-key ( coronavir* or coronovirus or "corona virus" or "corono virus" or hcov* or "covid-19" or covid19* or "covid 19" or "2019-ncov" or cv19* or "cv-19" or "cv 19" or "n-cov" or ncov* or "sars-cov-2" ) or ( wuhan* and ( virus or viruses or viral or coronav* ) ) or ( novel and coronav* ) or ( "2019-ncov" or 2019ncov or ncov2019 or "ncov-2019" or "covid-19" or covid19 or "hcov-19" or hcov19 or cov or "2019 novel*" or ncov or "n-cov" or "sars-cov-2" or "sarscov-2" or sarscov2 or sarscov2 or sarscov19 or "sars-cov-19" ) or ( coronavirus ) ) |
| 2 | Neurological diseases & syndromes | (brain[Title/Abstract] OR neuro*[Title/Abstract] OR CNS[Title/Abstract] OR nerv*[Title/Abstract] OR PNS[Title/Abstract] OR cereb*[Title/Abstract] OR myelit*[Title/Abstract] OR myelo*[Title/Abstract] OR radicul*[Title/Abstract] OR cranial[Title/Abstract] OR mening*[Title/Abstract] OR encephalit*[Title/Abstract] OR encephalop*[Title/Abstract] OR delirium[Title/Abstract] OR coma[Title/Abstract] OR (“acute disseminated encephalomyelitis”[Title/Abstract] OR ADEM[Title/Abstract]) OR confusion[Title/Abstract] OR seizure[Title/Abstract] OR convuls*[Title/Abstract] OR epilep*[Title/Abstract] OR ataxi*[Title/Abstract] OR rhombenceph*[Title/Abstract] OR paraly*[Title/Abstract] OR (“Guillain-Barré”[Title/Abstract] OR “Guillain Barré”[Title/Abstract] OR “Guillain-Barre”[Title/Abstract] OR “Guillain Barre”[Title/Abstract] OR GBS[Title/Abstract]) OR “Miller Fisher”[Title/Abstract] OR neurit*[Title/Abstract] OR neuromusc*[Title/Abstract] or myopath*[Title/Abstract] OR myosit*[Title/Abstract] OR stroke[Title/Abstract] OR (“transient ischaemic attack"[Title/Abstract] OR TIA[Title/Abstract]) OR neuroimag*[Title/Abstract] OR cognit*[Title/Abstract] OR CSF[Title/Abstract] OR "spinal fluid"[Title/Abstract] OR bickerstaff*[Title/Abstract] OR "movement disorder"[Title/Abstract] OR haemorrhag*[Title/Abstract] OR hemorrhag*[Title/Abstract] OR ischaem*[Title/Abstract] OR ischem*[Title/Abstract] OR vasculit*[Title/Abstract]) OR vasculop*[Title/Abstract]) | ( title-abs-key ( brain or neuro* or cns or nerv* or pns or cerebr* or cerebe* or myelit* or myelop* or myelorad* or radicul* or cranial or mening* or encephalit* or encephalop* or delirium or coma or ( "acute disseminated encephalomyelitis" or adem ) or confusion or seizure or convuls* or epilep* or ataxi* or rhombenceph* or paraly* or ( "guillain-barré" or "guillain barré" or "guillain-barre" or "guillain barre" or gbs ) or "miller fisher" or neurit* or neuromusc* or myopath* or myosit* or stroke or ( "transient ischaemic attack" or "transient ischemic attack" or tia ) or neuroimag* or cognit* or csf or "spinal fluid" or bickerstaff* or "movement disorder" or haemorrhag* or hemorrhag* or ischaem* or ischem* or vasculit* or vasculop*) ) |
| 3 | COVID-19 and neurological diseases/syndromes | 1 AND 2 | 1 AND 2 |
| 4 | Date limit | From 01/01/2020 to 03/07/2020 | (LIMIT-TO ( PUBYEAR ,  2020 ) ) |

**Table S2: Covariates and confounders for regression models**

| **Variable** | **Variable name** | **sex** | **age** | **sob; cough** | **comorb_diabetes** | **comorb_ccd** | **comorb_obesity** | **comorb_dementia** | **comorb_chronicneuro** | **CRP** | **tx_steroids** | **tx_antic** | **anti_plat** | **d_dimer** | **lymphocyte** | **whoregion** | **wbgroup** |
| --- | --- | --- | --- | --- | --- | --- | --- | --- | --- | --- | --- | --- | --- | --- | --- | --- | --- |
| **Sex at birth** | sex | NA | **Yes** | Yes | **Yes** | **Yes** | **Yes** | **Yes** | Yes | Yes | **Yes** | Yes | Yes | Yes | Yes | No | No |
| **Age** | age | **Yes** | NA | Yes | **Yes** | **Yes** | **Yes** | **Yes** | Yes | Yes | **Yes** | Yes | Yes | Yes | Yes | Yes | Yes |
| **Presentation with breathlessness** | sob | **Yes** | **Yes** | NA | **Yes** | **Yes** | Unknown | **Yes** | Unknown | Yes | **Yes** | Yes | Unknown | Yes | Yes | No | No |
| **Presentation with cough** | cough | **Yes** | **Yes** | NA | **Yes** | **Yes** | Unknown | **Yes** | Unknown | Yes | **Yes** | Yes | Unknown | Yes | Yes | No | No |
| **Diabetes mellitus** | comorb_diabetes | **Yes** | **Yes** | Yes | NA | **Yes** | **Yes** | No | No | Unknown | **Yes** | Yes | Unknown | Unknown | Unknown | Yes | Yes |
| **Cardiac disease** | comorb_ccd | **Yes** | **Yes** | Yes | **Yes** | NA | **Yes** | Unknown | Unknown | Unknown | **Yes** | Yes | Yes | Unknown | Unknown | Yes | Yes |
| **Obesity** | comorb_obesity | **Yes** | **Yes** | Unknown | **Yes** | **Yes** | NA | No | No | Unknown | **Yes** | Yes | Yes | Unknown | Unknown | Yes | Yes |
| **Dementia** | comorb_dementia | **Yes** | **Yes** | Yes | No | Unknown | No | NA | Yes | Unknown | **Yes** | Yes | Yes | Unknown | Unknown | Yes | Yes |
| **Other pre-existing neurological disease** | comorb_chronicneuro | **Yes** | **Yes** | Unknown | No | Unknown | No | **Yes** | NA | Unknown | Unknown | Unknown | Unknown | Unknown | Unknown | Unknown | Unknown |
| **Initial serum C-reactive protein concentration** | CRP | **Yes** | **Yes** | Yes | Unknown | Unknown | Unknown | Unknown | Unknown | NA | **Yes** | Unknown | Unknown | Unknown | Unknown | Unknown | Unknown |
| **Corticosteroids during admission** | tx_steroids | **Yes** | **Yes** | Yes | **Yes** | **Yes** | **Yes** | **Yes** | Unknown | Yes | NA | Yes | Unknown | Yes | Yes | No | No |
| **Anticoagulants during admission** | tx_antic | **Yes** | **Yes** | Yes | **Yes** | **Yes** | **Yes** | **Yes** | Unknown | Unknown | **Yes** | NA | Unknown | Yes | Unknown | Unknown | Unknown |
| **Antiplatelets pre-admission** | anti_plat | **Yes** | **Yes** | Unknown | Unknown | **Yes** | **Yes** | **Yes** | Unknown | Unknown | Unknown | Unknown | NA | Unknown | Unknown | Yes | Yes |
| **Initial blood D-dimer concentration** | d_dimer | **Yes** | **Yes** | Yes | Unknown | Unknown | Unknown | Unknown | Unknown | Unknown | **Yes** | Yes | Unknown | NA | Unknown | Unknown | Unknown |
| **Initial blood lymphocyte count** | lymphocyte | **Yes** | **Yes** | Yes | Unknown | Unknown | Unknown | Unknown | Unknown | Unknown | **Yes** | Unknown | Unknown | Unknown | NA | Unknown | Unknown |
| **WHO regions** | whoregion | No | **Yes** | No | **Yes** | **Yes** | **Yes** | **Yes** | Unknown | Unknown | No | Unknown | Yes | Unknown | Unknown | NA | Yes |
| **World bank income groups** | wbgroup | No | **Yes** | No | **Yes** | **Yes** | **Yes** | **Yes** | Unknown | Unknown | No | Unknown | Yes | Unknown | Unknown | Yes | NA |

No = variable is not a confounder; Yes = some evidence for confounding; **Yes (bold)** = strong evidence for confounding (included as confounders within the adjusted multivariable models); Unknown = unclear if variable is a confounder.

**Table S3: Summary of characteristics of studies providing individual patient data**

| **Study Details** | | | | | **Setting** | | | | | **Recruitment Period** | | | **Patient Characteristics** | | | | | | **Outcomes Reported** | | |
| --- | --- | --- | --- | --- | --- | --- | --- | --- | --- | --- | --- | --- | --- | --- | --- | --- | --- | --- | --- | --- | --- |
| **Study ID** | **Corresponding author** | **Study design** | **Chronology of participant recruitment** | **Publication status** | **Country** | **Number of sites** | **Hospitals** | **WHO Region** | **World Bank Income Group** | **Start date** | **End date** | **Duration, months** | **Patients with COVID-19 & neurological disease** | **Neurological disease diagnoses reported** | **Age in years, mean (range)** | **Sex at birth, n (%) male** | **Adults** | **Children** | **Discharge, death or follow-up to 30 days reported** | **Died, n (%)** | **mRS available, n (%)** |
| 1 | Ravaglia | Case series | Prospective | Some published, ^25,43^ some unpublished | Italy | 1 | IRCCS C. Mondino Foundation (1) | European | High | 08/03/2020 | 30/05/2020 | 2.8 | 10 | 3, 5, 6, 10, 11 | 56.7 (24-78) | 7 (70) | 10 | 0 | Yes | 0 (0) | 6 (60) |
| 2 | Fajardo Modol | Case series | Retrospective | Unpublished | Spain | 1 | Consorci Sanitari de Terrassa (1) | European | High | 02/03/2020 | 30/06/2020 | 4 | 6 | 2, 6, 10 | 63.8 (54-73) | 4 (66.7) | 6 | 0 | Yes | 2 (33.3) | 6 (100) |
| 3 | Karimi | Case series | Retrospective | Some published, ^44-47^ some unpublished | Iran | 1 | Bou Ali-Sina Hospital (1) | Eastern Mediterranean | Upper middle | 26/02/2020 | 30/06/2020 | 4.2 | 10 | 3, 6, 10 | 64 (30-85) | 5 (50) | 10 | 0 | Yes | 5 (50) | 10 (100) |
| 4 | Alkeridy | Case series | Retrospective | Some published, ^48,49^ some unpublished | Saudi Arabia | 1 | KKUH (1) | Eastern Mediterranean | High | 20/03/2020 | 01/07/2020 | 3.4 | 48 | 2, 3, 8, 10, 11 | 58.4 (17-85) | 33 (68.8) | 47 | 1 | Yes | 20 (41.7) | 48 (100) |
| 5 | Tattevin | Case series | Retrospective | Published*^50^ | France | 1 | Rennes university hospital (1) | European | High | 18/03/2020 | 05/04/2020 | 0.6 | 5 | 2, 3, 10 | 58.4 (48-72) | 5 (100) | 5 | 0 | Yes | 0 (0) | 4 (80) |
| 6 | Lorenz | Case report | Prospective | Published^51^ | Germany | 1 | Dresden Municipal Hospital (1) | European | High | 27/03/2020 | 23/07/2020 | 3.9 | 1 | 3 | 0 (0-0) | 0 (0) | 0 | 1 | Yes | 0 (0) | 1 (100) |
| 7 | Beretta | Case series | Retrospective | Some published, ^52^ some unpublished | Italy | 1 | San Gerardo Hospital (1) | European | High | 01/03/2020 | 30/04/2020 | 2 | 5 | 2, 6, 10 | 68.6 (55-79) | 1 (20) | 5 | 0 | Yes | 1 (20) | 5 (100) |
| 10^53^ | Foschi | Case series | Retrospective | Some published, some unpublished | Italy | 1 | S.Maria delle Croci (1) | European | High | 01/03/2020 | 31/05/2020 | 3 | 2 | 6, 10 | 61 (52-70) | 1 (50) | 2 | 0 | Yes | 0 (0) | 1 (50) |
| 11 | Balloy | Case report | Prospective | Published^54^ | France | 1 | University hospital center Nantes (1) | European | High | 26/03/2020 | 26/03/2020 | 0 | 1 | 2 | 59 (59-59) | 1 (100) | 1 | 0 | Yes | 0 (0) | 1 (100) |
| 12 | Klok | Single group cohort | Retrospective | Published^55,56^ | Netherlands | 3 | Amphia Hospital, Erasmus Medical Centre, University of Leiden Medical Centre (3) | European | High | 11/03/2020 | 13/04/2020 | 1.1 | 10 | 10 | 70.9 (58-87) | 6 (60) | 10 | 0 | Yes | 5 (50) | 5 (50) |
| 13 | Brito Ferreira | Case series | Prospective | Unpublished | Brazil | 1 | Hospital da Restauração (1) | Americas | Upper middle | 12/03/2020 | 12/07/2020 | 4.1 | 11 | 2, 3, 4, 6, 10 | 43.2 (16-74) | 9 (81.8) | 10 | 1 | Yes | 1 (9.1) | 11 (100) |
| 14 | Beltrán-Corbellini | Case-control | Retrospective | Published^57^ | Spain | 1 | University Hospital Ramón y Cajal (1) | European | High | 23/03/2020 | 25/03/2020 | 0.1 | 36 | 2, 8, st | 56.3 (28-90) | 21 (58.3) | 36 | 0 | Yes | 3 (8.3) | 36 (100) |
| 15 | Gunasekaran | Case series | Retrospective | Some published, ^58^ some unpublished | USA | 1 | Yale New Haven Health Bridgeport Hospital (1) | Americas | High | 23/03/2020 | 30/05/2020 | 2.3 | 2 | 2, 10 | 58.5 (40-77) | 0 (0) | 2 | 0 | Yes | 1 (50) | 2 (100) |
| 18 | Bernard-Valnet | Case series | Retrospective | Some published, ^59,60^ some unpublished | Switzerland | 1 | Centre Hospitalier Universitaire Vaudois (1) | European | High | 19/02/2020 | 12/04/2020 | 1.8 | 32 | 1, 2, 3, 6, 8, 10, 11 | 70 (45-94) | 22 (68.8) | 32 | 0 | Yes | 8 (25) | 28 (87.5) |
| 20 | Cabreira | Case series | Retrospective | Unpublished | Portugal | 1 | Centro Hospitalar Universitário de São João (1) | European | High | 13/03/2020 | 01/07/2020 | 3.7 | 4 | 2, 3 | 75.5 (66-82) | 2 (50) | 4 | 0 | Yes | 1 (25) | 4 (100) |
| 21 | Morrison | Case report | Retrospective | Unpublished | UK | 1 | Gloucestershire Royal Hospital (1) | European | High | 25/03/2020 | 01/06/2020 | 2.3 | 1 | 4 | 55 (55-55) | 1 (100) | 1 | 0 | Yes | 0 (0) | 1 (100) |
| 23 | Yenkoyan | Case series | Retrospective | Unpublished | Armenia | 1 | Heratsi University Hospital (1) | European | Upper middle | 17/06/2020 | 18/07/2020 | 1 | 7 | 3, 6, 10, st | 66.4 (45-79) | 4 (57.1) | 7 | 0 | Yes | 1 (14.3) | 3 (42.9) |
| 24 | Wagner | Cross-sectional | Prospective | Some published,^61^ some unpublished | Germany | 2 | University Hospital Freiburg, University Medical Centre (2) | European | High | 27/03/2020 | 02/05/2020 | 1.2 | 63 | 2, 8, 10, 11, st | 61.1 (25-89) | 37 (58.7) | 63 | 0 | Yes | 2 (3.2) | 61 (96.8) |
| 25 | Harkness | Case series | Retrospective | Published*^9^ | UK | 1 | Royal Hallamshire Hospital (1) | European | High | 18/03/2020 | 24/04/2020 | 1.2 | 7 | 10 | 83.6 (69-98) | 2 (28.6) | 7 | 0 | Yes - partly reported | 3 (42.9) | 6 (85.7) |
| 26 | Kaya | Case series | Retrospective | Published^62,63^ | Turkey | 1 | Acibadem Fulya Hospital (1) | European | Upper middle | 18/03/2020 | 25/05/2020 | 2.3 | 10 | 2, 3, 8, 10, 11 | 71.2 (39-92) | 6 (60) | 10 | 0 | Yes | 1 (10) | 9 (90) |
| 27 | Meza | Case series | Retrospective | Unpublished | Peru | 1 | Trujillo Regional Hospital (1) | Americas | Upper middle | 15/03/2020 | 03/07/2020 | 3.7 | 21 | 2, 5, 6, 10, st | 50 (20-80) | 12 (57.1) | 21 | 0 | Yes - partly reported | 7 (33.3) | 15 (71.4) |
| 28 | Ñancupil Reyes | Case report | Prospective | Unpublished | Chile | 1 | Barros Luco Trudeu Hospital (1) | Americas | High | 01/05/2020 | 01/07/2020 | 2 | 1 | 3 | 79 (79-79) | 1 (100) | 1 | 0 | Yes | 0 (0) | 0 (0) |
| 29 | Maggi | Case series | Prospective | Unpublished | Belgium | 2 | Cliniques Universitaires Saint-Luc, Université Catholique de Louvain (2) | European | High | 15/03/2020 | 30/04/2020 | 1.5 | 12 | 1, 2, 3, 10, 11 | 66.3 (37-85) | 9 (75) | 12 | 0 | Yes - partly reported | 1 (8.3) | 9 (75) |
| 31 | Fernández Díaz | Cross-sectional | Retrospective | Published^64^ | Spain | 1 | Complejo Hospitalario Universitario de Albacete (1) | European | High | 01/03/2020 | 19/04/2020 | 1.6 | 339 | 2, 3, 4, 6, 9, 10, 11, st | 71.1 (29-100) | 190 (56) | 339 | 0 | Yes | 124 (36.6) | 132 (38.9) |
| 33 | Sharifi-Razavi | Case series | Retrospective | Some published,^46,47^ some unpublished | Iran | 1 | Bou Ali-Sina Hospital (1) | Eastern Mediterranean | Upper middle | 20/02/2020 | 25/06/2020 | 4.2 | 14 | 2, 3, 5, 8, 10 | 63.8 (26-88) | 6 (42.9) | 14 | 0 | Yes | 6 (42.9) | 14 (100) |
| 36 | Paterson | Case series | Retrospective | Published^65^ | UK | 1 | National Hospital for Neurology & Neurosurgery (1) | European | High | 25/03/2020 | 26/04/2020 | 1.1 | 10 | 2, 3, 5, 6, 11 | 52.7 (16-65) | 5 (50) | 9 | 1 | Yes - partly reported | 0 (0) | 1 (10) |
| 37 | Mariotto | Cross-sectional | Prospective | Published^66^ | Italy | 1 | University of Verona (1) | European | High | 15/03/2020 | 30/06/2020 | 3.6 | 18 | 2, 3, 10, 11, st | 68.7 (54-85) | 14 (77.8) | 18 | 0 | Yes | 1 (5.6) | 18 (100) |
| 39 | Lahiri | Case series | Prospective | Some published,^67^ some unpublished | India | 4 | Burdwan Medical College and Hospital, M.R. Bangur Hospital, R.G. Kar Medical College and Hospital, IPGMER and SSKM Hospital (4) | South East Asia | Lower middle | 01/04/2020 | 08/07/2020 | 3.3 | 15 | 1, 2, 3, 4, 6, 10, 11 | 54.9 (18-88) | 8 (53.3) | 15 | 0 | Yes - partly reported | 5 (33.3) | 7 (46.7) |
| 40 | Morassi | Case series | Retrospective | Some published,^68^ some unpublished | Italy | 1 | Fondazione Poliambulanza Hospital (1) | European | High | 20/02/2020 | 15/05/2020 | 2.8 | 31 | 2, 3, 6, 8, 10, 11 | 70.9 (40-86) | 21 (67.7) | 31 | 0 | Yes | 10 (32.3) | 31 (100) |
| 41 | Lechien | Case series | Retrospective | Published^69,70^ | Belgium | 2 | EpiCURA Hospital, UMONS Hospital (2) | European | High | 31/01/2020 | 04/05/2020 | 3.1 | 94 | 2, 11, st | 75.5 (39-95) | 53 (56.4) | 94 | 0 | Yes - partly reported | 41 (43.6) | 78 (83) |
| 42 | Moein | Case series | Prospective | Published^71^ | Iran | 1 | Masih Daneshvari Hospital (1) | Eastern Mediterranean | Upper middle | 20/03/2020 | 06/04/2020 | 0.6 | 78 | 11, st | 46.7 (23-76) | 55 (70.5) | 78 | 0 | Yes | 0 (0) | 78 (100) |
| 44 | Abd-Allah | Case series | Prospective | Unpublished | Egypt | 1 | Kasr Alainy Hospital (1) | Eastern Mediterranean | Lower middle | 01/05/2020 | 10/07/2020 | 2.3 | 4 | 1, 5, 8, 10 | 53.8 (41-65) | 3 (75) | 4 | 0 | Yes - partly reported | 1 (25) | 2 (50) |
| 45 | Kimani | Case report | Retrospective | Unpublished | Kenya | 1 | Halcyon Hospital (1) | African | Lower middle | 11/05/2020 | 11/05/2020 | 0 | 1 | 3 | 35 (35-35) | 1 (100) | 1 | 0 | No | 0 (0) | 1 (100) |
| 46 | Santoro | Case series | Retrospective | Unpublished | USA | 1 | Children's Hospital Los Angeles (CHLA) (1) | Americas | High | 19/04/2020 | 06/06/2020 | 1.6 | 3 | 2, 6, 11 | 9 (2-17) | 3 (100) | 0 | 3 | Yes | 0 (0) | 3 (100) |
| 47 | Jain | Case series | Retrospective | Published^72-75^ | USA | 1 | NYU Langone Health (1) | Americas | High | Unknown |  | Unknown | 38 | 3, 10, 11 | 65.7 (40-86) | 28 (73.7) | 38 | 0 | Yes - partly reported | 16 (42.1) | 16 (42.1) |
| 48 | Khawaja | Single group cohort | Prospective | Unpublished | USA | 1 | Detroit Medical Center (1) | Americas | High | 01/03/2020 | 30/06/2020 | 4 | 61 | 2, 3, 4, 8, 10, 11 | 63.5 (29-94) | 32 (52.5) | 61 | 0 | Yes - partly reported | 27 (44.3) | 28 (45.9) |
| 49 | Singh | Case report | Retrospective | Unpublished | India | 1 | DMC Hospital Ludhiana (1) | South East Asia | Lower middle | Unknown |  | Unknown | 1 | 5 | 32 (32-32) | 1 (100) | 1 | 0 | No | 0 (0) | 0 (0) |
| 50 | Abdelnour | Case series | Retrospective | Some published,^76^ some unpublished | UK | 1 | Ulster Hospital (1) | European | High | 10/03/2020 | 30/06/2020 | 3.7 | 8 | 1, 2, 3, 11 | 64.3 (43-77) | 5 (62.5) | 8 | 0 | Yes | 1 (12.5) | 7 (87.5) |
| 53 | Staufenberg | Case series | Retrospective | Unpublished | UK | 1 | Norfolk and Norwich University Hospital (1) | European | High | 15/03/2020 | 04/05/2020 | 1.7 | 10 | 2, 11 | 86 (69-94) | 6 (60) | 10 | 0 | Yes | 6 (60) | 6 (60) |
| 54 | Jillella | Case series | Retrospective | Published^77^ | USA | 1 | Emory University School of Medicine (1) | Americas | High | 01/03/2020 | 31/05/2020 | 3 | 11 | 10 | 60.5 (43-75) | 6 (54.5) | 11 | 0 | Yes | 4 (36.4) | 11 (100) |
| 57 | Guekht | Case series | Retrospective | Unpublished | Russia | 1 | Moscow Research and Clinical Center for Neuropsychiatry (1) | European | Upper middle | 28/04/2020 | 25/06/2020 | 1.9 | 15 | 2, 4, 10, 11 | 64.3 (33-92) | 5 (33.3) | 15 | 0 | Yes | 0 (0) | 12 (80) |
| 59 | Werring | Case series | Prospective | Published^78^ | UK | 1 | University College London Hospital (1) | European | High | 28/03/2020 | 12/04/2020 | 0.5 | 6 | 10 | 68.2 (53-85) | 5 (83.3) | 6 | 0 | Yes - partly reported | 1 (16.7) | 6 (100) |
| 60 | Defres | Case series | Prospective | Some published,^79^ some unpublished | UK | 1 | Royal Liverpool Hospital (1) | European | High | 15/01/2020 | 31/05/2020 | 4.6 | 69 | 2, 3, 10, 11 | 75.9 (28-94) | 41 (59.4) | 68 | 0 | Yes - partly reported | 34 (49.3) | 34 (49.3) |
| 61 | Contini | Case series | Prospective | Unpublished | Australia | 1 | St Vincent's Hospital (1) | Western Pacific | High | 31/03/2020 | 29/06/2020 | 3 | 2 | 2, 3, 11 | 67 (61-73) | 2 (100) | 2 | 0 | Yes | 0 (0) | 2 (100) |
| 62 | Elicer | Case series | Retrospective | Unpublished | Chile | 1 | Hospital Dr.Sótero del Río (1) | Americas | High | 27/03/2020 | 15/07/2020 | 3.7 | 33 | 2, 3, 6, 8, 10 | 61.8 (31-84) | 14 (42.4) | 33 | 0 | Yes - partly reported | 7 (21.2) | 27 (81.8) |
| 63 | Valdoleiros | Case series | Retrospective | Unpublished | Portugal | 1 | Centro Hospitalar Universitário do Porto (1) | European | High | 01/03/2020 | 22/04/2020 | 1.7 | 40 | 2, 10, 11, st | 72 (24-92) | 20 (50) | 40 | 0 | Yes | 8 (20) | 39 (97.5) |
| 65 | Raith | Case series | Retrospective | Unpublished | UK | 1 | National Hospital for Neurology and Neurosurgery (1) | European | High | 01/03/2020 | 14/07/2020 | 4.5 | 2 | 3, 6 | 56 (52-60) | 2 (100) | 2 | 0 | No | 0 (0) | 0 (0) |
| 66 | Kaimovsky | Case series | Retrospective | Unpublished | Russia | 1 | Moscow City Clinical Hospital after V.M. Buyanov (1) | European | Upper middle | 28/04/2020 | 25/06/2020 | 1.9 | 11 | 2, 7, 10, 11 | 68.1 (52-80) | 8 (72.7) | 11 | 0 | Yes | 0 (0) | 8 (72.7) |
| 67 | Bigaut | Case series | Prospective | Some published,^80^ some unpublished | France | 2 | Strasbourg University Hospital, CHU Grenoble Alpes (2) | European | High | 28/03/2020 | 11/04/2020 | 0.5 | 3 | 4, 6 | 55.3 (43-70) | 2 (66.7) | 3 | 0 | Yes - partly reported | 0 (0) | 2 (66.7) |
| 68 | Hassell | Case series | Prospective | Some published,^81,82^ some unpublished* | UK | 1 | Great Ormond Street Hospital for Children (1) | European | High | 01/04/2020 | 01/06/2020 | 2 | 23 | 1, 2, 3, 4, 8, 9, 11 | 9.6 (1-17) | 14 (60.9) | 0 | 23 | Yes - partly reported | 0 (0) | 22 (95.7) |
| 69 | Liguori | Case series | Prospective | Published^83^ | Italy | 1 | University Hospital of Rome Tor Vergata (1) | European | High | 16/04/2020 | 05/05/2020 | 0.6 | 2 | 1, 2 | 73.5 (72-75) | 1 (50) | 2 | 0 | Yes | 2 (100) | 2 (100) |
| 70 | Atef | Case series | Retrospective | Unpublished | Egypt | 1 | Cairo University Affiliated Hospitals (1) | Eastern Mediterranean | Lower middle | 30/01/2020 | 30/06/2020 | 5.1 | 14 | 2, 3, 5, 6, 8, 10 | 58.1 (33-80) | 9 (64.3) | 14 | 0 | Yes | 5 (35.7) | 14 (100) |
| 73 | Benger | Case series | Retrospective | Published^84^ | UK | 1 | King's College Hospital (1) | European | High | 01/02/2020 | 14/05/2020 | 3.4 | 5 | 10 | 53.2 (41-64) | 3 (60) | 5 | 0 | Yes - partly reported | 0 (0) | 4 (80) |
| 74 | Heyse | Case report | Retrospective | Published^85^ | Belgium | 1 | AZ Glorieux Hospital (1) | European | High | 09/03/2020 | 13/04/2020 | 1.2 | 1 | 10 | 74 (74-74) | 0 (0) | 1 | 0 | Yes | 1 (100) | 1 (100) |
| 75 | Pinto | Case report | Prospective | Published^86^ | UK | 1 | Southampton General Hospital (1) | European | High | 14/04/2020 | 14/04/2020 | 0 | 1 | 4 | 44 (44-44) | 0 (0) | 1 | 0 | Yes | 0 (0) | 1 (100) |
| 78 | Guijarro-Castro | Case series | Retrospective | Some published,^87^ some unpublished | Spain | 3 | Hospital Universitario HM Sanchinarro, Hospital San Juan de Dios Pamplona, CINAC Puerta del Sur (3) | European | High | Unknown |  | Unknown | 19 | 2, 7, 8, 10, 11, st | 42 (23-70) | 10 (52.6) | 17 | 0 | Yes - partly reported | 0 (0) | 0 (0) |
| 80 | Pincherle | Case report | Prospective | Unpublished | Luxembourg | 1 | Robert Schuman Hospital (HRS) (1) | European | High | 13/04/2020 | 13/04/2020 | 0 | 1 | 8 | 82 (82-82) | 1 (100) | 1 | 0 | Yes | 0 (0) | 1 (100) |
| 81 | Garcia Garcia | Case series | Retrospective | Published^88^ | Spain | 1 | Hospital Universitario Rio Hortega (1) | European | High | 24/03/2020 | 24/04/2020 | 1 | 4 | 10 | 67 (59-81) | 2 (50) | 4 | 0 | Yes | 2 (50) | 4 (100) |
| 83 | Sotoca | Case series | Retrospective | Some published,^89^ some unpublished | Spain | 1 | Hospital Universitari Mútua Terrassa (1) | European | High | 12/03/2020 | 01/05/2020 | 1.7 | 6 | 2, 5, 8, 10 | 64.7 (37-80) | 3 (50) | 6 | 0 | Yes | 1 (16.7) | 6 (100) |
| 84 | Pinna | Case series | Retrospective | Published^90^ | USA | 1 | Rush University Medical Center (1) | Americas | High | 14/03/2020 | 05/05/2020 | 1.7 | 44 | 2, 3, 10, 11, st | 59.8 (33-89) | 25 (56.8) | 44 | 0 | Yes - partly reported | 7 (15.9) | 9 (20.5) |
| 85 | Seyed Alinaghi | Case series | Retrospective | Some published,^91^ some unpublished | Iran | 2 | Ziaeian Hospital, Imam Khomeini Hospital (2) | Eastern Mediterranean | Upper middle | 10/03/2020 | 10/03/2020 | 0 | 2 | 6 | 55.5 (43-68) | 0 (0) | 2 | 0 | Yes | 1 (50) | 1 (50) |
| 86 | Soares | Case series | Prospective | Some published,^92,93^ some unpublished | Brazil | 1 | Hospital Federal dos Servidores do Estado (1) | Americas | Upper middle | 01/04/2020 | 01/07/2020 | 3 | 10 | 3, 5, 6, 8, 11 | 49 (29-76) | 3 (30) | 10 | 0 | Yes | 1 (10) | 10 (100) |
| 87 | Diezma-Martin | Case report | Retrospective | Published^94^ | Spain | 1 | Complejo Hospitalario de Toledo (1) | European | High | 30/03/2020 | 30/03/2020 | 0 | 1 | 11 | 70 (70-70) | 1 (100) | 1 | 0 | Yes | 0 (0) | 1 (100) |
| 88 | D'Anna | Case series | Retrospective | Published^95^ | UK | 1 | Charing Cross Hospital (1) | European | High | 15/03/2020 | 26/04/2020 | 1.4 | 8 | 10 | 73.3 (55-88) | 7 (87.5) | 8 | 0 | Yes - partly reported | 0 (0) | 7 (87.5) |
| 89 | Winkler | Case series | Prospective | Unpublished | Germany | 1 | Klinikum rechts der Isar - Technical University of Munich (1) | European | High | 01/03/2020 | 01/06/2020 | 3.1 | 17 | 2, 8, 10, 11 | 61.4 (26-89) | 11 (64.7) | 17 | 0 | Yes | 4 (23.5) | 17 (100) |
| 90 | Boso | Case report | Retrospective | Published^96^ | Italy | 1 | S.Maria del Carmine Hospital (1) | European | High | 29/03/2020 | 15/05/2020 | 1.6 | 1 | 6 | 67 (67-67) | 0 (0) | 1 | 0 | Yes | 0 (0) | 1 (100) |
| 91 | Umapathi | Single group cohort | Prospective | Published^11^ | Singapore | 6 | National Neuroscience Institute, National University Health System, Changi General Hospital, Ng Teng Fong General Hospital, Sengkang General Hospital, Khoo Teck Puat Hospital (6) | Western Pacific | High | 19/03/2020 | 19/07/2020 | 4.1 | 39 | 3, 4, 8, 10, 11 | 45.2 (27-73) | 38 (97.4) | 39 | 0 | Yes - partly reported | 5 (12.8) | 37 (94.9) |
| 92 | Scullen | Case series | Retrospective | Published^97^ | USA | 1 | Ochsner Medical Center (1) | Americas | High | 01/03/2020 | 01/05/2020 | 2 | 27 | 1, 2, 11 | 59.8 (35-91) | 14 (51.9) | 27 | 0 | Yes - partly reported | 12 (44.4) | 22 (81.5) |
| 93 | Primiano | Case series | Retrospective | Some published,^98^ some unpublished | Italy | 1 | Fondazione Policlinico Universitario A. Gemelli IRCCS (1) | European | High | 14/03/2020 | 20/07/2020 | 4.3 | 77 | 2, 3, 8, 10, 11, st | 77.8 (47-97) | 43 (55.8) | 73 | 0 | Yes - partly reported | 32 (41.6) | 74 (96.1) |
| 94 | Yin | Case series | Retrospective | Some published,^99^ some unpublished | China | 1 | The 940th Hospital of Joint Logistic Support Force of the PLA (1) | Western Pacific | Upper middle | 04/02/2020 | 14/04/2020 | 2.3 | 16 | 2, 5, 10 | 71.1 (52-96) | 10 (62.5) | 16 | 0 | Yes - partly reported | 2 (12.5) | 16 (100) |
| 95 | de Moura Brasil Matos | Case series | Prospective | Unpublished | Brazil | 2 | Hospital Geral de Fortaleza, Instituto de Infectologia Emílio Ribas (2) | Americas | Upper middle | 11/05/2020 | 23/07/2020 | 2.4 | 17 | 2, 3, 6, 8, 10, 11 | 45.6 (20-79) | 7 (41.2) | 17 | 0 | Yes - partly reported | 2 (11.8) | 16 (94.1) |
| 96 | Garazzino | Case report | Retrospective | Published^100^ | Italy | 1 | Regina Margherita Children's Hospital (1) | European | High | 15/03/2020 | 01/07/2020 | 3.6 | 1 | 3 | 14 (14-14) | 0 (0) | 0 | 1 | Yes | 0 (0) | 1 (100) |
| 99 | Barrios-López | Case series | Retrospective | Published^101^ | Spain | 1 | Hospital Universitario Virgen de las Nieves (1) | European | High | 25/03/2020 | 17/04/2020 | 0.8 | 4 | 10 | 71.5 (50-87) | 2 (50) | 4 | 0 | Yes | 2 (50) | 4 (100) |
| 100 | Huda | Case series | Retrospective | Unpublished | UK | 1 | The Walton Centre Foundation Trust (1) | European | High | 04/04/2020 | 08/05/2020 | 1.1 | 2 | 8, 11 | 46.5 (39-54) | 0 (0) | 2 | 0 | Yes | 0 (0) | 2 (100) |
| 102 | Pilotto | Single group cohort | Prospective | Published^102-105^ | Italy | 1 | ASST Spedali Civili Brescia (1) | European | High | 20/02/2020 | 01/06/2020 | 3.4 | 106 | 2, 3, 6, 8, 10, 11 | 72.1 (40-91) | 52 (49.1) | 106 | 0 | Yes - partly reported | 35 (33) | 106 (100) |
| 104 | Elkady | Case series | Retrospective | Some published,^106^ some unpublished | Egypt | 1 | Mataria Teaching Hospital (1) | Eastern Mediterranean | Lower middle | 15/03/2020 | 08/06/2020 | 2.8 | 7 | 2, 6, 10 | 44.1 (27-67) | 4 (57.1) | 7 | 0 | Yes | 1 (14.3) | 7 (100) |
| 105 | Thakur | Case series | Retrospective | Some published,^107^ some unpublished | USA | 1 | Columbia University Irving Medical Center (1) | Americas | High | 27/02/2020 | 22/05/2020 | 2.8 | 31 | 1, 2, 3, 4, 10 | 42 (0.01-80) | 24 (77.4) | 23 | 8 | Yes | 6 (19.4) | 31 (100) |
| 106 | Coen | Case series | Retrospective | Some published,^108,109^ some unpublished | Switzerland | 1 | Geneva University Hospital (1) | European | High | 15/03/2020 | 10/05/2020 | 1.9 | 14 | 2, 6, 10, 11 | 66.1 (25-91) | 9 (64.3) | 14 | 0 | Yes - partly reported | 1 (7.1) | 12 (85.7) |
| 107 | Delorme | Single group cohort | Retrospective | Unpublished | France | 1 | Pitié-Salpêtrière Hospital (1) | European | High | 06/03/2020 | 25/05/2020 | 2.7 | 231 | 2, 3, 5, 6, 8, 10, 11 | 63.6 (18-98) | 140 (60.6) | 231 | 0 | Yes - partly reported | 9 (3.9) | 9 (3.9) |
| 108 | Nalleballe | Case series | Retrospective | Published^110^ | USA | 1 | University of Arkansas for Medical Sciences (UAMS) (1) | Americas | High | 01/02/2020 | 01/06/2020 | 4 | 3 | 2, 10 | 53.3 (46-59) | 2 (66.7) | 3 | 0 | Yes | 2 (66.7) | 3 (100) |
| 109 | Beach | Case series | Retrospective | Published^111^ | USA | 1 | Massachusetts General Hospital (1) | Americas | High | 04/03/2020 | 07/04/2020 | 1.1 | 4 | 2 | 73 (68-78) | 3 (75) | 4 | 0 | Yes | 1 (25) | 4 (100) |
| 110 | Rice | Case series | Retrospective | Unpublished | UK | 1 | Southmead Hospital (1) | European | High | 28/03/2020 | 09/04/2020 | 0.4 | 4 | 2, 4, 10, 11 | 58 (50-74) | 4 (100) | 4 | 0 | Yes | 0 (0) | 4 (100) |
| 112 | Escalard | Single group cohort | Prospective | Published^112^ | France | 1 | Fondation Rothschild Hospital (1) | European | High | 15/03/2020 | 30/04/2020 | 1.5 | 15 | 10 | 61.6 (44-80) | 12 (80) | 15 | 0 | Yes - partly reported | 8 (53.3) | 15 (100) |

~Studies were pooled into a single database of anonymised IPD. Studies from the same centre or location were checked for duplication, with the dataset providing the most complete data being fed into the IPD database.

* Not published at the time we received data, but since published.

^ Neurological disease diagnoses: 1 - meningitis, 2 - encephalopathy, 3 - encephalitis, 4 - ADEM, 5 - Myelitis, 6 - GBS, 8 - peripheral neuropathy, 10 - cerebrovascular event, 11- other neurological disease, st - smell or taste disturbance only

**Table S4: Included studies and aggregate or individual patient data (IPD) grouped according to World Health Organisation and World Bank Region**

|  | **IPD – published** | |  | **IPD - not published** | |  |
| --- | --- | --- | --- | --- | --- | --- |
|  | **Studies (n = 52)** | **Patients (n = 1337)** |  | **Studies (n = 31)** | **Patients (n = 642)** |  |
| ***WHO Regions*** | | | | | | |
| **African/Eastern Mediterranean** | 6 (12%) | 159 (12%) |  | 3 (10%) | 19 (3%) |  |
| **Americas** | 8 (15%) | 160 (12%) |  | 8 (26%) | 157 (24%) |  |
| **European** | 35 (67%) | 948 (71%) |  | 18 (58%) | 463 (72%) |  |
| **South-East Asia/Western Pacific** | 3 (6%) | 70 (5%) |  | 2 (6%) | 3 (0%) |  |
| ***World Bank Income Groups*** | | | | | | |
| **Low- and lower-middle income country** | 2 (4%) | 22 (2%) |  | 4 (13%) | 20 (3%) |  |
| **Upper-middle income country** | 6 (12%) | 130 (10%) |  | 7 (23%) | 92 (14%) |  |
| **High-income country** | 44 (85%) | 1185 (89%) |  | 20 (65%) | 530 (83%) |  |

**Table S5: Quality assessment results for studies providing individual patient data**

**Table S5A: Case reports***

| **Study ID** | **Primary Contact** | **1. Demographics** | **2. History** | **3. Presenting condition** | **4. Assessment** | **5.Treatment** | **6. Discharge condition** | **7. Adverse events** |
| --- | --- | --- | --- | --- | --- | --- | --- | --- |
| 6 | Lorenz | Yes | Yes | No | Yes | Yes | Yes | NA |
| 11 | Balloy | Yes | Yes | Yes | Yes | Yes | Yes | NA |
| 21 | Morrison | Yes | Yes | Yes | Yes | Yes | Yes | NA |
| 28 | Ñancupil Reyes | Yes | Yes | Yes | Yes | Yes | Yes | NA |
| 45 | Kimani | Yes | Yes | Yes | No | Yes | No | NA |
| 49 | Singh | No | No | No | No | No | No | NA |
| 74 | Duroi | Yes | Yes | Yes | Yes | Yes | Yes | NA |
| 75 | Pinto | Yes | Yes | Yes | Yes | Yes | Yes | NA |
| 80 | Pincherle | Yes | Yes | Yes | Yes | Yes | Yes | NA |
| 87 | Diezma-Martin | Yes | Yes | Yes | Yes | Yes | Yes | Yes |
| 90 | Boso | Yes | Yes | Yes | Yes | Yes | Yes | NA |
| 96 | Garazzino | Yes | Yes | Yes | Yes | Yes | Yes | NA |

* The majority of case reports described patient demographics, history, presenting symptoms, and details of discharge from hospital, diagnostic tests, and interventions (provided by between 10 and 11 of the 12 reports in each domain).

NA: not applicable

Full questions from the JBI Case Reports quality assessment tool:
1. Were patient’s demographic characteristics clearly described?
2. Was the patient’s history clearly described and presented as a timeline?
3. Was the current clinical condition of the patient on presentation clearly described?
4. Were diagnostic tests or assessment methods and the results clearly described?
5. Was the intervention(s) or treatment procedure(s) clearly described?
6. Was the post-intervention clinical condition clearly described?
7. Were adverse events (harms) or unanticipated events identified and described?
8. Does the case report provide takeaway lessons?

Question 8 was not applicable for any of these studies, as none were published as individual case reports.

**Table S5B: Case series**

| **Study ID** | **Primary contact** | **1. Inclusion criteria** | **2. Condition measurement** | **3. Identification methods** | **4. Consecutive inclusion** | **5. Complete inclusion** | **6. Demographics** | **7. Clinical information** | **8. Outcomes** | **9. Site information** |
| --- | --- | --- | --- | --- | --- | --- | --- | --- | --- | --- |
| 1 | Ravaglia | Yes | Unclear | Yes | Unclear | No | Yes | Yes | Yes | Yes |
| 2 | Fajardo Modol | Yes | Unclear | Yes | Unclear | No | Yes | Yes | Yes | Yes |
| 3 | Karimi | Yes | Unclear | Yes | Unclear | No | Yes | Yes | Yes | Yes |
| 4 | Alkeridy | Yes | Unclear | Yes | Unclear | No | Yes | Yes | Yes | Yes |
| 5 | Tattevin | Yes | Unclear | Yes | Unclear | No | Yes | Yes | Yes | Yes |
| 7 | Beretta | Yes | Unclear | Yes | Unclear | No | Yes | Yes | Yes | Yes |
| 10 | Foschi | Yes | Unclear | Yes | Yes | Yes | Yes | Yes | No | Yes |
| 13 | Brito Ferreira | Yes | Unclear | Yes | Unclear | No | Yes | Yes | Yes | Yes |
| 14 | Beltran-Corbellini | Yes | Unclear | Yes | Yes | Yes | Yes | Yes | Yes | Yes |
| 15 | Gunasekaran | Yes | Unclear | Yes | Yes | Yes | Yes | Yes | Yes | Yes |
| 18 | Bernard-Valnet | Yes | Unclear | Yes | Unclear | No | Yes | No | Yes | Yes |
| 20 | Cabreira | Yes | Unclear | Yes | Unclear | No | Yes | Yes | Yes | Yes |
| 23 | Yenkoyan | Yes | Unclear | Yes | Unclear | No | Yes | No | No | Yes |
| 25 | Harkness | Yes | Unclear | Yes | Unclear | Unclear | Yes | Yes | Yes | Yes |
| 26 | Kaya | Yes | Unclear | Yes | Yes | Yes | Yes | Yes | No | Yes |
| 27 | Meza | Yes | Unclear | Yes | Unclear | No | Yes | Yes | Yes | Yes |
| 29 | Maggi | Yes | Unclear | Yes | Unclear | No | Yes | Yes | Yes | Yes |
| 33 | Sharifi Razavi | Yes | Unclear | Yes | No | No | Yes | No | No | Yes |
| 36 | Paterson | Yes | Unclear | Yes | Unclear | No | Yes | Yes | No | Yes |
| 39 | Lahiri | Yes | Unclear | Yes | Unclear | No | Yes | Yes | No | Yes |
| 40 | Morassi | Yes | Unclear | Yes | Unclear | No | Yes | Yes | Yes | Yes |
| 41 | Lechien | Yes | Unclear | Yes | Yes | Yes | Yes | Yes | No | Yes |
| 42 | Moein | Yes | Unclear | Yes | Unclear | No | Yes | Yes | Yes | Yes |
| 44 | Abd-Allah | Yes | Unclear | Yes | Yes | Yes | Yes | Yes | No | Yes |
| 46 | Santoro | Yes | Unclear | Yes | Unclear | No | Yes | Yes | Yes | Yes |
| 47 | Jain | Yes | Unclear | Yes | Unclear | No | Yes | No | No | Yes |
| 50 | Abdelnour | Yes | Unclear | Yes | Unclear | No | Yes | Yes | Yes | Yes |
| 53 | Staufenberg | Yes | Unclear | Yes | Unclear | No | No | No | No | Yes |
| 54 | Jillela | Yes | Unclear | Yes | Unclear | No | Yes | No | No | Yes |
| 57 | Guekht | Yes | Unclear | Yes | Unclear | No | Yes | Yes | No | Yes |
| 59 | Werring | Yes | Unclear | Yes | Unclear | No | Yes | Yes | Yes | Yes |
| 60 | Defres | Yes | Unclear | Yes | Unclear | No | Yes | No | No | Yes |
| 61 | Contini | Yes | Unclear | Yes | Unclear | No | Yes | Yes | Yes | Yes |
| 62 | Elicer | Yes | Unclear | Yes | Unclear | No | Yes | Yes | Yes | Yes |
| 63 | Valdoleiros | Yes | Unclear | Yes | Unclear | No | Yes | Yes | Yes | Yes |
| 65 | Raith | Yes | Unclear | Yes | Unclear | No | Yes | Yes | Yes | Yes |
| 66 | Kaimovsky | Yes | Unclear | Yes | Unclear | No | Yes | Yes | Yes | Yes |
| 67 | Bigaut | Yes | Unclear | Yes | Unclear | No | Yes | Yes | Yes | Yes |
| 68 | Hassell | Yes | Unclear | Yes | Unclear | No | Yes | Yes | Yes | Yes |
| 69 | Liguori | Yes | Unclear | Yes | Unclear | No | Yes | No | Yes | Yes |
| 70 | Atef | Yes | Unclear | Yes | Unclear | No | Yes | Yes | Yes | Yes |
| 73 | Benger | Yes | Unclear | Yes | Yes | No | Yes | Yes | Yes | Yes |
| 78 | Guijarro-Castro | Yes | Unclear | Yes | No | No | No | No | No | No |
| 81 | Garcia Garcia | Yes | Unclear | Yes | Unclear | No | Yes | Yes | Yes | Yes |
| 83 | Sotoca | Yes | Unclear | Yes | Yes | Yes | Yes | Yes | Yes | Yes |
| 84 | Pinna | Yes | Unclear | Yes | Yes | No | Yes | No | No | Yes |
| 85 | Seyed Alinaghi | Yes | Unclear | Yes | Unclear | No | Yes | No | No | Yes |
| 86 | Soares | Yes | Unclear | Yes | Unclear | No | Yes | Yes | Yes | Yes |
| 88 | D'Anna | Yes | Unclear | Yes | Unclear | No | Yes | Yes | Yes | Yes |
| 89 | Winkler | Yes | Unclear | Yes | Unclear | Unclear | Yes | Yes | Yes | Yes |
| 92 | Scullen | Yes | Unclear | Yes | Yes | Yes | Yes | No | Yes | Yes |
| 93 | Primiano | Yes | Unclear | Yes | Yes | Yes | Yes | No | Yes | Yes |
| 94 | Yin | Yes | Unclear | Yes | Yes | Yes | Yes | No | Yes | Yes |
| 95 | de Moura Brasil Matos | Yes | Unclear | Yes | No | No | Yes | Yes | Yes | Yes |
| 99 | Barrios-López | Yes | Unclear | Yes | Unclear | No | Yes | Yes | Yes | Yes |
| 100 | Huda | Yes | Unclear | Yes | Unclear | No | Yes | Yes | Yes | Yes |
| 104 | Elkady | Yes | Unclear | Yes | Unclear | No | Yes | Yes | Yes | Yes |
| 105 | Thakur | Yes | Unclear | Yes | No | No | No | No | Yes | Yes |
| 106 | Coen | Yes | Unclear | Yes | Unclear | No | Yes | Yes | Yes | Yes |
| 108 | Nalleballe | Yes | Unclear | Yes | Unclear | No | Yes | Yes | Yes | Yes |
| 109 | Beach | Yes | Unclear | Yes | Unclear | No | Yes | Yes | Yes | Yes |
| 110 | Rice | Yes | Unclear | Yes | Unclear | No | Yes | Yes | Yes | Yes |

* All 61 case series used our pre-defined inclusion criteria; the majority provided detail on patient demographics (59 studies), clinical information (47 studies), outcomes (46 studies) and site of recruitment (60 studies). Eight studies reported they had recruited all patients with COVID-19 and new neurological disease seen at their centre; 10 studies recruited consecutive eligible patients. We assessed case-control studies using the case series tool, as only the cases from these studies were described in the datasets contributed.

NA: not applicable

Full questions from the JBI Case Series quality assessment tool:
1. Were there clear criteria for inclusion in the case series?
2. Was the condition measured in a standard, reliable way for all participants included in the case series?
3. Were valid methods used for identification of the condition for all participants included in the case series?
4. Did the case series have consecutive inclusion of participants?
5. Did the case series have complete inclusion of participants?
6. Was there clear reporting of the demographics of the participants in the study?
7. Was there clear reporting of clinical information of the participants?
8. Were the outcomes or follow up results of cases clearly reported?
9. Was there clear reporting of the presenting site(s)/clinic(s) demographic information?
10. Was statistical analysis appropriate?

Question 10 was not applicable for any of these studies, as we did not seek details of statistical analysis for individual case series.

**Table S5C: Cross-sectional and single-group cohort studies**

| **Study ID** | **Primary Contact** | **Study design** | **SELECTION** | | | | **COMPARABILITY** | | **OUTCOME** | |
| --- | --- | --- | --- | --- | --- | --- | --- | --- | --- | --- |
|  |  |  | **Representativeness** | **Sample size** | **Non-respondents** | **Exposure ascertainment** | **Most important factor** | **Additional factor** | **Outcome assessment** | **Statistical test** |
| 12 | Klok | Single-group cohort | Somewhat | Not justified | Comparable & satisfactory response | No description | No | No | Independent, blind | Yes |
| 24 | Wagner | Cross-sectional | Somewhat | Not justified | No description | No description | No | No | Independent, blind | No |
| 31 | Fernandez Diaz | Cross-sectional | Truly representative | Not justified | Comparable & satisfactory response | No description | No | No | Independent, blind | No |
| 37 | Mariotto | Cross-sectional | Truly representative | Not justified | No description | No description | Yes | No | Independent, blind | No |
| 48 | Khawaja | Single-group cohort | No description | Not justified | No description | No description | No | No | No description | No |
| 91 | Umapathi | Single-group cohort | Somewhat | Not justified | No description | No description | No | No | Independent, blind | No |
| 102 | Pilotto | Single-group cohort | Truly representative | Not justified | No description | No description | No | No | Independent, blind | No |
| 107 | Delorme | Single-group cohort | Truly representative | Not justified | No description | No description | No | No | Independent, blind | No |
| 112 | Escalard | Single-group cohort | Selected group | Not justified | Comparable & satisfactory response | No description | No | No | Independent, blind | No |

* Of the six cross-sectional studies and the three single-group cohorts, most (8 of 9) used independent blind assessment of outcomes, though answers for other domains were more likely to be answered negatively, highlighting lower quality.

Full questions and options from the Newcastle-Ottawa Scale cohort study quality assessment tool, adapted for cross-sectional studies by Herzog et al 2012, which we also used for single-group cohort studies:
*Selection*
1) Representativeness of the sample

a) truly representative of the average patients in the target population (all subjects or random sampling)
b) somewhat representative of the average in the target population. (non-random sampling)
c) selected group of users
d) no description of the sampling strategy

2) Sample size

a) Justified and satisfactory.
b) Not justified.

3) Non-respondents:

a) Comparability between respondents and non-respondents’ characteristics is established, and the response rate is satisfactory.
b) The response rate is unsatisfactory, or the comparability between respondents and non-respondents is unsatisfactory. 
c) No description of the response rate or the characteristics of the responders and the non-responders.

4) Ascertainment of the exposure (risk factor):

a) Validated measurement tool.
b) Non-validated measurement tool, but the tool is available or described.
c) No description of the measurement tool.

*Comparability*
1) Comparability of cohorts on the basis of the design or analysis

a) study controls for the most important factor
b) study controls for any additional factor (These criteria could be modified to indicate specific control for a second important factor.) *[Note that for comparability questions, a relevant example (age/delirium) has been given for the most important factor for a condition as the outcome.]*

*Outcome*
1) Assessment of outcome

a) independent blind assessment
b) record linkage
c) self-report
d) no description

2) Statistical test:

a) The statistical test used to analyse the data is clearly described and appropriate, and the measurement of the association is presented, including confidence intervals and the probability level (p value).
b) The statistical test is not appropriate, not described or incomplete.

**Table S6: Level of severity of COVID-19 for patients included in the IPD database**

| COVID-19 Severity | All patients | Cerebrovascular Events | Encephalopathy |
| --- | --- | --- | --- |
| Asymptomatic | 57 (2.9%) | 39 (7.7%) | 10 (1.0%) |
| Mild - no hypoxia or pneumonia | 243 (12.3%) | 53 (10.5%) | 67 (6.9%) |
| Moderate - pneumonia | 313 (15.8%) | 99 (19.6%) | 89 (9.1%) |
| Severe - severe pneumonia | 293 (14.8%) | 92 (18.2%) | 145 (14.8%) |
| Critical - ARDS | 440 (22.2%) | 139 (27.5%) | 217 (22.2%) |
| Critical - sepsis | 82 (4.1%) | 9 (1.8%) | 56 (5.7%) |
| Critical - septic shock | 72 (3.6%) | 20 (4.0%) | 39 (4.0%) |
| Unknown | 479 (24.2%) | 55 (10.9%) | 355 (36.3%) |
| Total | 1979 | 506 | 978 |

1. WHO criteria was used to determine COVID-19 severity for individual cases, and is displayed in Supplementary appendix S2, Section 3.1: COVID-19 case definitions.

**Table S7: Level of certainty in the neurological diagnosis for patients included in the IPD database**

| Diagnosis | Number of patients |
| --- | --- |
| Encephalopathy, including encephalitis |  |
| *Level 1 - Encephalitis* | 39 |
| *Level 2 - Possible encephalitis* | 72 |
| *Level 3 - Encephalopathy* | 844 |
| *Level 4 - Suspected encephalopathy* | 22 |
| *Total with certainty level assessed* | 977 |
| Meningitis |  |
| *Level 1 - Meningitis* | 7 |
| *Level 2 - Possible meningitis* | 4 |
| *Level 3 - Meningism* | 2 |
| *Level 4 - Suspected meningitis* | 2 |
| *Total with certainty level assessed* | 15 |
| ADEM^5^ |  |
| *Level 1 - ADEM* | 7 |
| *Level 2 - Probable ADEM* | 1 |
| *Level 3 - Suspected ADEM* | 2 |
| *Total with certainty level assessed* | 10 |
| Myelitis |  |
| *Level 1 - Myelitis* | 9 |
| *Level 2 - Possible myelitis* | 0 |
| *Level 3 - Myelopathy* | 4 |
| *Level 4 - Suspected myelopathy* | 0 |
| *Total with certainty level assessed* | 13 |
| Guillain-Barré syndrome |  |
| *Level 1* | 24 |
| *Level 2* | 20 |
| *Level 3* | 5 |
| *Level 4* | 2 |
| *Total with certainty level assessed* | 51 |
| Central nervous system vasculitis^5^ |  |
| *Definite* | 0 |
| *Possible* | 2 |
| *Total with certainty level assessed* | 2 |

1. Levels 1-4 indicate the degree of certainty of neurological syndrome diagnosis.

2. Definitions of these levels of certainty were based on proposed criteria published by our group earlier in the pandemic and are available in appendix S2, Section 3.2: Neurological case definitions.

3. Level of certainty was not assessed for the following diagnoses: cerebrovascular event; radiculitis; peripheral neuropathy; myositis; ‘other’ presentation; or in cases of more than one co-existing diagnosis.

4. Denominators may differ from table 1, as not all patients had a level of certainty available, and some patients with level 2 ‘possible encephalitis’ were included in the delirium, coma and encephalopathy-other subgroups in Table 1.

5. Some diagnoses did not have four pre-defined levels of certainty: ADEM had three and vasculitis had two.

**Table S8: Strength of association between SARS-CoV-2 and neurological disease for patients in the IPD database ^1,2,3^**

| **Disease** | **Definite or Confirmed** | **Probable** | **Possible** | **Total^3^** |
| --- | --- | --- | --- | --- |
| Cerebrovascular event | 0 | 92 | 362 | 454 |
| Central nervous system vasculitis |  |  | 1 | 1 |
| Encephalopathy, including encephalitis | 0 | 792 | 34 | 826 |
| Meningitis | 0 | 9 | 2 | 11 |
| Acute Disseminated Encephalomyelitis (ADEM) | 0 | 12 | 2 | 14 |
| Myelitis/myelopathy | 2 | 10 | 1 | 11 |
| Guillain-Barré syndrome | 0 | 26 | 19 | 45 |
|  |  |  |  |  |

1. Definite or confirmed, probable and possible indicate the differing degrees of association between COVID-19 and the diagnosed neurological syndrome.

2. The strength of the evidence for an association of SARS-CoV-2 with the reported neurological disease was assessed using proposed criteria published by our group earlier in the pandemic, and are available in appendix s2, Section 3.3: Neurological COVID-19 association.

3. Not all diagnoses had pre-defined criteria for strength of association. These were not available for: radiculitis; peripheral neuropathy; or myositis.

4. These denominators reflect the number of patients for whom an assessment of strength of association is available, and not the total number of patients with each diagnosis.

**Table S9: Clinical outcomes of encephalopathy subgroup and encephalopathy subgroup without encephalitis^1^**

|  | **Encephalopathy subgroup** | | **Encephalopathy without encephalitis** | |
| --- | --- | --- | --- | --- |
| **Modified Rankin scale (mRS) score at discharge^1,2^** | **Probability of being at each mRS score^3^** | **Cumulative probability of being at each mRS score or worse (95% CI)** | **Probability of being at each mRS score^3^** | **Cumulative probability of being at each mRS score or worse (95% CI)** |
| **6 - Dead** | 17% | 17% (11-25) | 17% | 17% (11-26) |
| **5 - Severe disability** | 7% | 24% (16-34) | 8% | 25% (17-36) |
| **4 - Moderately severe disability** | 13% | 37% (27-48) | 15% | 41% (29-53) |
| **3 - Moderate disability** | 17% | 54% (42-65) | 18% | 59% (46-70) |
| **2 - Slight disability** | 15% | 69% (58-78) | 14% | 73% (62-81) |
| **1 - No significant disability despite symptoms** | 22% | 91% (85-94) | 21% | 94% (89-96) |
| **0 - No symptoms at all** | 9% |  | 6% |  |
| **In-hospital mortality at 30 days^3^** | 38% (95% CI 34-42) | | 39% (95% CI 35-43) | |
| **Cumulative incidence of admission to critical care, or invasive mechanical ventilation at 30 days^3^** | 38% (95% CI 34-42) | | 36% (95% CI 32-40) | |
| **Cumulative incidence of discharge from critical care at 30 days** | 53% (95% CI 46-59) | | 53% (95% CI 46-59) | |
| **Cumulative incidence of discharge from hospital at 30 days** | 49% (95% CI 46-52) | | 49% (95% CI 46-53) | |

1. Clinical outcomes are described for the total encephalopathy subgroup and described again for the encephalopathy subgroup excluding patients with a diagnosis of encephalitis.

**Table S10: Characteristics of four studies reporting proportions of total neurological disease contributed by each diagnosis from the IPD database ^1,2^**

| **Study ID** | **Country** | **Patients w/ COVID-19 neurological disease** | **Age, mean (SD)** | **Sex at birth: female,**  **N (%)** | **HTN**  **N (%)** | **Diabetes**  **N (%)** | **Cardiac disease (incl. AF),**  **N (%)** | **Obesity N (%)** | **Dementia N (%)** | **Pre-existing neuro disease**  **N (%)** | **HIV or other immune-suppression N (%)** | **Antiplatelets pre-admission**  **N (%)** | **Cortico-steroids during admission N (%)** | **Anticoagulants during admission**  **N (%)** | **Critical COVID-19**  **N (%)^3^** | **CRP (mg/L) Mean (SD)** | **Cerebrovascular event**  **N (%)** | **Encephalopathy, including encephalitis**  **N (%)** | **ADEM**  **N (%)** | **Myelitis**  **N (%)** | **GBS**  **N (%)** | **Peripheral neuropathy**  **N (%)** | **Myositis N (%)** | **Other neurological presentation or >1 syndrome**  **N (%)** | **Time in days from onset of typical COVID-19 symptoms to neurological disease**  **median (IQR)** |  |
| --- | --- | --- | --- | --- | --- | --- | --- | --- | --- | --- | --- | --- | --- | --- | --- | --- | --- | --- | --- | --- | --- | --- | --- | --- | --- | --- |
| 31 | Spain | 339 | 71  (14.5) | 149  (44.0) | 205  (60.5) | 96  (28.3) | 73  (22.0) | 66 (51.6) | 31 (9.2) | 3 (15) | 16 (4.9) | 48 (25) | 187 (55.8) | 143 (46.6) | 6 (46) | 143.3 (191.5) | 30 (8.8) | 259 (76.4) | 1 (0.3) | 0 (0) | 1 (0.3) | 0 (0) | 1 (0.3) | 0 (0) | 20.5 (19-24) |  |
| 91 | Singapore | 39 | 45  (12.7) | 1  (2.6) | 14  (35.9) | 5  (12.8) | 5 (12.8) | 3 (7.7) | 0 (0) | 0 (0) | 0 (0) | 3 (7.7) | 6 (15.4) | 9 (23.1) | 9 (23) | 31.1 (47.8) | 25 (64.1) | 2 (5.1) | 2 (5.1) | 0 (0) | 0 (0) | 6 (15.4) | 0 (0) | 0 (0) | 15 (3-25) |  |
| 102 | Italy | 106 | 72  (12.3) | 54  (50.9) | 53  (50) | 27  (25.5) | 6 (5.7) | 2 (1.9) | 2 (1.9) | 15 (14.3) | 1 (0.9) | 4 (28.6) | 28 (26.4) | 66 (66.7) | 23 (22) | 50.8 (66.7) | 68 (64.2) | 30 (28.3) | 0 (0) | 0 (0) | 2 (1.9) | 1 (0.9) | 0 (0) | 1 (0.3) | Not available |  |
| 107 | France | 231 | 64  (16.7) | 91  (39.3) | 110  (47.6) | 70  (30.3) | 0 (0) | 49 (21.2) | 0 (0) | 0 (0) | 14 (6.1) | 0 (0) | 0 (0) | 0 (0) | 112 (49) | Not available | 37 (16) | 116 (50.2) | 0 (0) | 1 (0.4) | 5 (2.2) | 37 (16) | 0 (0) | 47 (13.9) | Not available |  |

IQR = interquartile range; HTN = hypertension; CRP = C-reactive protein; ADEM = Acute disseminated encephalomyelitis; GBS = Guillain Barré Syndrome

1. The quality assessments of these studies showed that the approaches for inclusion of patients with COVID-19 neurological disease were heterogeneous, there was wide variation in proportions between studies, and only a few studies were eligible, so we therefore decided calculating pooled proportions by meta-analysis would not be appropriate.

2. Meningitis and radiculitis are not included in this table, as there were no patients with those diagnoses in any of these studies.

3. Using WHO criteria for COVID-19 severity,^12^ where the information was available. Note that for study 31, this was unknown for the majority of patients.

**Table S11: Studies contributing data for proportion of total COVID-19 cases who have acute new-onset neurological disease from the IPD database**

| **Study** | **Country** | **Number and type(s) of centre** | **Study design** | **Start date** | **End date** | **Neurological disease groups included** | **Total patients with COVID-19 Neurological disease** | **Total patients with any COVID-19** | **Percentage of total COVID-19 patients with acute new-onset neurological disease (%)** |
| --- | --- | --- | --- | --- | --- | --- | --- | --- | --- |
| 10 | Italy | Single tertiary centre | Case series | 01/03/2020 | 31/05/2020 | GBS & ischaemic stroke | 2 | 123 | 1.6 |
| 14 | Spain | Single tertiary centre | Case-control | 23/03/2020 | 25/03/2020 | CNS; PNS; focus on smell & taste disturbance | 41 | 84 | 48.8 |
| 31 | Spain | Two centres: one tertiary; one general | Retrospective cohort | 01/03/2020 | 01/04/2020 | CNS; PNS | 327 | 841 | 38.9 |
| 41 | Belgium | Single tertiary centre | Retrospective cross-sectional | 31/01/2020 | 04/05/2020 | CNS, PNS, focus on smell & taste disturbance | 94 | 294 | 32.0 |
| 83 | Spain | Single tertiary centre | Case series | 12/03/2020 | 01/05/2020 | CNS; PNS | 6 | 580 | 1.0 |
| 91 | Singapore | National: all centres admitting patients with COVID-19 | Prospective cross-sectional | 19/03/2020 | 19/07/2020 | CNS; PNS; excluded smell & taste disturbance as sole reason | 39 | 47533 | 0.08 |
| 93 | Italy | Single tertiary centre | Case series | 14/03/2020 | 20/07/2020 | CNS; PNS | 77 | 220 | 35.0 |
| 107 | France | Single tertiary centre | Retrospective cohort | 06/03/2020 | 25/05/2020 | CNS; PNS; neuropsychiatric | 246 | 1979 | 12.4 |

CNS = central nervous system; GBS = Guillain-Barré syndrome; PNS = peripheral nervous system
