# Supplementary material for: Prognostic indicators and outcomes of hospitalised COVID-19 patients with neurological disease: An individual patient data meta-analysis
Source: PLoS One. 2022 Jun 2;17(6):e0263595. doi: 10.1371/journal.pone.0263595 (PMC9162376; doi:10.1371/journal.pone.0263595)
Supplement: S2 Appendix — (PDF) [file pone.0263595.s003.pdf]

Supplementary appendix S2 – Data extraction tool, dictionary, and case definitions

Prognostic indicators and outcomes of hospitalised COVID-19 patients with neurological disease: a systematic review and individual patient data meta-analysis

Contents

Section 1: Data extraction tool
Section 2: Dictionary for data extraction tool
Section 3: Case definitions
- **3.1:** COVID-19 case definitions
- **3.2:** Neurological case definitions
- **3.3:** Neurological COVID-19 association

Section 1: Data extraction tool

			Patient record
**Clinician details**			
	Name of responsible clinician		
	Role and specialty of responsible clinician		
	Hospital		
	Town/City		
	Country		
**Admission details**			
	Date of patient's hospital admission (dd/mm/yy)		
**Patient demographics**			
	Age		
	Sex		
	Ethnicity		
	Pregnant		
		If 'YES', gestational weeks:	
	Patient recruited to other research studies?		
**Comorbidities**			
		Hypertension	
		Chronic cardiac disease	
		Atrial fibrillation	
		Diabetes mellitus	
		Obesity	
		HIV or other immunocompromise	
		Malignancy	
		Dementia	
		History of previous stroke	
		Other chronic neurological disorder	
		Other comorbidity	
		If 'YES', please give details of other comorbidity:	
		Smoker/ smoking history	
		History of excessive alcohol consumption	
**Medication pre-admission**			
		Anti-hypertensives	
		Anti-platelets	
		Anti-coagulation	
		Immunomodulatory therapy – oral steroids or other immunosuppressant agents	
		Other pre-admission medication	
**COVID-19: Systemic Infection**			
	**Did the patient have suspected, probable or confirmed COVID-19? (See Tab 3 Table 1)**		
		If 'suspected' due to contact with case, indicate date of contact if known (dd/mm/yy)	
	**COVID-19 disease severity (See Tab 3 Table 2)**		
	**Date of COVID-19 symptom onset** (dd/mm/yy)		
	**Date of COVID-19 symptom resolution** (dd/mm/yy) **-** if symptoms ongoing, leave blank		
	Clinical features of COVID-19 infection		
		fever	
		lethargy	
		myalgia	
		coryza	
		loss of smell (anosmia)	
		loss of taste (ageusia)	

sore throat
cough
chest pain
shortness of breath
diarrhoea
abdominal pain

Blood results - please indicate units

Initial haemoglobin
Initial lymphocyte count
Lowest lymphocyte count
Initial neutrophil count
Initial platelet count
Initial CRP
Initial D-dimer
Highest D-dimer
Initial ferritin
Initial creatinine
Initial LDH
Initial Prothrombin time
Blood culture performed?
If YES, select result:
HIV
Malaria screen (RDT/film/smear)
Scrub typhus serology
Japanese encephalitis serology
Dengue serology
Other non-COVID-19 blood results:

Chest Imaging

Chest X-ray performed?
If 'YES', Abnormalities?
date perfomed [dd/mm/yy]:
Chest CT perfomed?
If 'YES', Evidence of pneumonia?
Evidence of ground glass abnormality?
Evidence of pulmonary embolism?
date perfomed [dd/mm/yy]:

COVID-19: Laboratory testing

Laboratory-confirmed SARS-2 infection
PCR positive - respiratory specimen
date of sample collection (dd/mm/yy)
What was the RT-PCR Cycle threshold (Ct) of the first respiratory specimen tested?
PCR positive - blood
date of sample collection (dd/mm/yy)
PCR positive - Cerebrospinal fluid
date of sample collection (dd/mm/yy)
IgM positive - serum
date of sample collection (dd/mm/yy)
IgM positive - cerebrospinal fluid
date of sample collection (dd/mm/yy)
IgG positive - serum

date of sample collection (dd/mm/yy)

IgG positive - cerebrospinal fluid

date of sample collection (dd/mm/yy)

If other method, please specify

date of sample collection (dd/mm/yy)

Additional respiratory pathogen testing

PCR Influenza

Positive test for other respiratory pathogen

If YES, describe pathogen, test and sample:

Neurological syndrome

Date of onset of neurological features (dd/mm/yy)

Neurological syndrome (select; see Tab 4 for Neuro Case Definitions)

Specific neurological syndrome:

If '**Encephalitis**' OR '**Encephalopathy** (includes **delirium** or **coma**)', select syndrome:

If 'Other' or additional details, describe:

If '**Guillain Barré Syndrome**', select syndrome:

If 'Other' or additional details, describe:

If '**Cerebrovascular event**', select:

If 'Other' or additional details, describe:

If '**Other neurological syndrome**', give details:

Level of evidence - neurological diagnosis:

See Tab 4 Neuro Case Definitions

If '**Meningitis**':

If '**Encephalitis**':

If '**Acute Disseminated Encephalomyelitis (ADEM)**':

If '**Myelitis**':

If '**Guillain Barré Syndrome**':

If '**Cerebovascular event - Vasculitis**':

Level of evidence - association between neurological disease and COVID-19:

See Tab 5 Neuro COVID19 association

Based on Tab 5, Table 1, indicate if the **association between SARS-CoV-2 infection and neurological disease** is confirmed / strong, probable, possible, or not described:

If 'Not described in Tab 5 Table 1', give details of evidence for association:

History of pre-existing neurological disorder?

if yes: give details (estimated data of diagnosis; neurological disorder)

Neurological symptoms

Headache

Reduced conscious level

Confusion

Delirium

If 'YES', Disturbance in attention and awareness?

Change from baseline that developed rapidly and fluctuates during the day?

Additional disturbance in cognition?

If 'YES' to additional disturbance in cognition, give details:

Behavioural change

Seizure(s)

if 'YES': focal, generalised, or other?

If 'Other', give details:

Visual distubance

if 'YES': visual field defect, diplopia, reduced acuity, or other?

If 'Other', give details:

Photophobia

If other neurological symptom(s), give details:

Neurological signs

AVPU
Glasgow Coma Score (/15) (lowest score recorded during admission)
Eye opening
Verbal response
Motor response
Neck stiffness
Disorientation
Witnessed seizures
New onset movement disorder
if 'YES': ataxia, chorea, tremor, myoclonus, bradykenesia or other?
If 'Other / combination of features', give details:
Cranial neuropathy
if yes: describe here
Peripheral neuropathy
if yes: describe here
Limb weakness
if yes: describe here
Sensory abnormality
if yes: describe here
Dysphagia
Dysphasia
if yes: describe here

Neurological Investigations

CSF results

Lumbar puncture performed?
If YES, date of lumbar puncture (dd/mm/yy)
CSF opening pressure [cm CSF/water]
CSF total white cell count [per µl or cumm or mm^3]
CSF neutrophil/polymorph count
Units for neutrophil/polymorph count
CSF lymphocyte/mononuclear cell count
Units for lymphocyte/mononuclear cell count
CSF red blood cells [per µl or cumm or mm^3]
CSF protein
Units for protein
CSF glucose
Paired blood glucose
Units for glucose
CSF albumin [g/L]
CSF oligoclonal bands
Other NON-MICROBIOLOGICAL CSF results

Microbiological CSF results

CSF Gram stain (record result if performed)
CSF bacterial culture (record result if performed)
CSF Herpes Simplex Virus PCR (HSV1 or HSV2 or combined)
CSF Varicella Zoster Virus PCR
CSF Enterovirus PCR
CSF TB PCR
CSF Other pathogen test
If YES, describe:

Neuroimaging

Computed Tomography (CT) of head
if yes, describe here and include date (dd/mm/yy):
MRI of brain
if yes, describe here and include date (dd/mm/yy):
MRI of spine
if yes, describe here and include date (dd/mm/yy):

Other neurological examinations

Electroencephalography (EEG)
if yes, describe here and include date (dd/mm/yy):
Nerve conduction study/ electromyography
if yes, describe here and include date (dd/mm/yy):

Were anti-neuronal antibody tests performed?

If 'YES', describe test and result:

Treatment in hospital

Did the patient have significant hypoxia (sp02 <90% or arterial hypoxaemia on blood gas) during admission?

Oxygen - maximum demand during admission [L/min or % - please indicate which] OR 'Room air only'
Antiviral treatment for COVID-19 (incl remdesivir, lopinavir/ritonavir, hydroxychloroquine etc.)
Convalescent plasma
Tocilizumab or other IL-6 inhibitor
Vasopressor/inotropic support
Anxiolytics or hypnotics
Corticosteroids
If 'YES' drug and dose
date started [dd/mm/yy]:
date stopped [dd/mm/yy] (leave blank if continued until now):
Indication:
Intravenous immunoglobulin
If 'YES' drug and dose
date started [dd/mm/yy]:
date stopped [dd/mm/yy] - leave blank if continued until now:
Indication:
Plasma exchange
If 'YES' date started [dd/mm/yy]:
date stopped [dd/mm/yy] - leave blank if continued until now:
Indication:
Anticoagulation
If 'YES' drug and dose
date started [dd/mm/yy]:
date stopped [dd/mm/yy] - leave blank if continued until now:
Indication:
Antiplatelets
If 'YES' drug and dose
date started [dd/mm/yy]:
date stopped [dd/mm/yy] - leave blank if continued until now:
Indication:
Thrombolysis
If 'YES' drug and dose
date given [dd/mm/yy]:
Thrombectomy/ mechanical clot retrieval
If 'YES' date performed [dd/mm/yy]:
Other treatment given/ other surgical management?
If 'YES', describe (drug/management; dose; date started and stopped; indication):

Outcome

	Admitted to high dependency or intensive care / intensive therapy unit?	
	Date of high dependency/ intensive care / intensive therapy unit admission [dd/mm/yy]	
	Date of high dependency/ intensive care / intensive therapy unit discharge [dd/mm/yy] - leave blank if still on ICU:	
	Non-invasive ventilation	
	Invasive ventilation	
	If YES Date of intubation [dd/mm/yy]	
	Date of extubation [dd/mm/yy]	
	Reintubated?	
	Tracheostomy?	
	Complications in hospital	
	If YES, give details (type of complication and date if known etc.):	
	**Died?**	
	If YES, Date of death [dd/mm/yy]	
	Describe cause of death:	
	If autopsy performed, give details:	
	**Has this patient been discharged?**	
	**If discharged, Modified Rankin Score at time of discharge**	
	**Date of discharge from hospital [dd/mm/yy]**	
	**Date of last follow up (whether patient is still in hospital, or following discharge) [dd/mm/yy]**	
**DENOMINATOR QUESTIONS**	1. How many neuro COVID-19 patients are included in your dataset?	
	2. Is this all the neuro COVID-19 patients that have been seen in your hospital/centre?	
	If, 'No', how many neuro COVID-19 patients have been seen in total in your hospital/ centre?	
	3. Over what time period were these neuro COVID-19 patients seen? (start date ddmmyy - end date ddmmyy)	
	4. How many non-neuro COVID-19 patients were seen in your centre during the same time period?	
**PUBLICATION QUESTIONS**	1. Were some/all of these data included as part of a study?	
	**If 'YES', please submit your protocol with these data**	
	What was your case definition?	
	Have the data been published?	
	If 'YES', provide 'DOI':	
	Was there a control/comparator group?	
	if 'YES', what was your definition of a control?	
	if 'YES', was it a case-control study?	
	2. Were data collected retrospectively or prospectively?	

Section 2: Dictionary for data extraction tool

Variable name	Values									
clinician_name										
clinician_role										
centre										
location_city										
location_country										
date_adm_hosp										
age										
sex	0 - Male	1 - Female	2 - Other	3 - Unknown						
ethnic	1 - Arab	2 - Black	3 - East Asian	4 - South Asian	5 - West Asian	6 - Latin American	7 - White	8 - Aboriginal	9 - First Nations	10 - Other
pregnancy	1 - Yes	0 - No	2 - Unknown							
weeks_pregnant										
other_studies	0 - No	1 - ISARIC CCP	2 - WHO-SOLIDARITY	3 - Other						
comorb_hypertension	1 - Yes	0 - No	2 - Unknown							
comorb_ccd	1 - Yes	0 - No	2 - Unknown							
comorb_af	1 - Yes	0 - No	2 - Unknown							
comorb_diabetes	1 - Yes	0 - No	2 - Unknown							
comorb_obesity	1 - Yes	0 - No	2 - Unknown							
comorb_hiv	1 - Yes	0 - No	2 - Unknown							
comorb_malignancy	1 - Yes	0 - No	2 - Unknown							
comorb_dementia	1 - Yes	0 - No	2 - Unknown							
comorb_stroke	1 - Yes	0 - No	2 - Unknown							
comorb_chronicneuro	1 - Yes	0 - No	2 - Unknown							
comorb_other	1 - Yes	0 - No	2 - Unknown							
comorb_detail										
smoke	1 - Yes	0 - No	2 - Unknown							
alcohol	1 - Yes	0 - No	2 - Unknown							
anti_htn	1 - Yes	0 - No	2 - Unknown							
anti_plat	1 - Yes	0 - No	2 - Unknown							
anti_coag	1 - Yes	0 - No	2 - Unknown							
immunotherapy	1 - Yes	0 - No	2 - Unknown							
comorb_other_detail										
covid_diag_label	1 - Confirmed	2 - Probable - test inconclusive	3 - Probable - test not done	4 - Probable - PCR negative, supportive blood tests/radiology	5 - Suspected - local transmission	6 - Suspected - contact with case	7 - Suspected - severe and no other aetiology identified	8 - Suspected - clinical suspicion		
date_cov_contact										
covid-severity	1 - Asymptomatic	2 - Mild - no hypoxia or pneumonia	3 - Moderate - pneumonia	4 - Severe - severe pneumonia	5 - Critical - ARDS	6 - Critical - sepsis	7 - Critical - septic shock	8 - Unknown		
date_cov_sym_onset										

date_cov_sym_resolution				
fever_hist	1 - Yes	0 - No	2 - Unknown	
lethargy	1 - Yes	0 - No	2 - Unknown	
myalgia	1 - Yes	0 - No	2 - Unknown	
coryza	1 - Yes	0 - No	2 - Unknown	
anosmia	1 - Yes	0 - No	2 - Unknown	
loss_of_taste	1 - Yes	0 - No	2 - Unknown	
sore_thr	1 - Yes	0 - No	2 - Unknown	
cough	1 - Yes	0 - No	2 - Unknown	
chest_pain	1 - Yes	0 - No	2 - Unknown	
sob	1 - Yes	0 - No	2 - Unknown	
diarrhoea	1 - Yes	0 - No	2 - Unknown	
abdo_pain	1 - Yes	0 - No	2 - Unknown	
Hb				
lymphocyte				
lowest_lymphocyte				
neut				
plat				
CRP				
d_dimer				
highest_d_dimer				
ferritin				
creat				
LDH				
ptt				
blood_culture	1 - Yes	0 - No	2 - Unknown	
blood_culture_result	0 - Negative	1 - Positive - bacteraemia	2 - Positive - other	3 - Unknown
HIV	0 - Negative	1 - Positive	2 - Not tested	
Malaria	0 - Negative	1 - Positive	2 - Not tested	
scrub_t	0 - Negative	1 - Positive	2 - Not tested	
JE	0 - Negative	1 - Positive	2 - Not tested	
denv	0 - Negative	1 - Positive	2 - Not tested	
other_path_results				
cxr	1 - Yes	0 - No		
cxr_abnormal	1 - Yes, unilateral	2 - Yes, bilateral	0 - No, normal	3 - Unknown
cxr_date				
chest_ct	1 - Yes	0 - No		
ct_pneumonia	1 - Yes, unilateral	2 - Yes, bilateral	0 - No	3 - Unknown
ct_gr_glass	1 - Yes, unilateral	2 - Yes, bilateral	0 - No	3 - Unknown
ct_pulm_emb	1 - Yes, unilateral	2 - Yes, bilateral	0 - No	3 - Unknown
SARS2_infection_confirmed	1 - Yes	0 - No		
PCR_resp	1 - Yes	0 - No	2 - Not tested	
PCR_resp_date				
PCR_Ct				

PCR_blood	1 - Yes	0 - No	2 - Not tested								
PCR_blood_date											
PCR_CSF	1 - Yes	0 - No	2 - Not tested								
PCR_CSF_date											
IgM_serum	1 - Yes	0 - No	2 - Not tested								
IgM_serum_date											
IgM_CSF	1 - Yes	0 - No	2 - Not tested								
IgM_CSF_date											
IgG_serum	1 - Yes	0 - No	2 - Not tested								
IgG_serum_date											
IgG_CSF	1 - Yes	0 - No	2 - Not tested								
IgG_CSF_date											
SARS2_method_other											
SARS2_method_other_date											
influ_resp	0 - Negative	1 - Positive	2 - Not tested								
other_resp_path	1 - Yes	0 - No	2 - Not tested								
other_resp_path_descr											
neuro_onset											
syndrome	1 - Meningitis	2 - Encephalopathy (no CNS inflammation) - including delirium or coma	3 - Encephalitis	4 - Acute Disseminated Encephalomyelitis (ADEM)	5 - Myelitis	6 - Guillain-Barré syndrome	7 - Radiculitis	8 - Peripheral neuropathy	9 - Myositis	10 - Cerebrovascular event	11 – Other neurological presentation
syndrome_encephalitis	1 - Encephalopathy - delirium	2 - Encephalopathy - coma	3 - Encephalopathy - other	4 - Posterior reversible encephalopathy syndrome (PRES)	5 - Malignant cerebral oedema	6 - Acute Necrotising Encephalopathy (ANE)	7 - Encephalitis - Other				
syndrome_encephalitis_desc											
syndrome_GBS	1 - Classic sensorimotor (all 4 limbs)	2 - Motor only (all 4 limbs)	3 - Paraparetic (lower limbs only)	4 - Pharyngeal–cervical–brachial weakness	5 - Bilateral facial palsy with paraesthesias	6 - Pure sensory	7 - Miller Fisher syndrome	8 - Bickerstaff brainstem encephalitis	9 - Other		
syndrome_GBS_desc											
syndrome_cerebrovascular	1 - TIA	2 - Ischaemic stroke - large artery	3 - Ischaemic stroke - cardioembolic	4 - Ischaemic stroke - small vessel occlusion	5 - Vasculitis	6 - Haemmorrhage	7 - Cerebral vein / sinus thrombosis	8 - Other			
syndrome_cerebrovascular_desc											
syndrome_other											
evidence_meningitis	1 - Meningitis	2 - Possible meningitis	3 - Meningism	4 - Suspected meningitis							
evidence_encephalitis	1 - Encephalitis	2 - Possible encephalitis	3 - Encephalopathy	4 - Suspected encephalopathy							
evidence_adem	1 - ADEM	2 - Possible ADEM	3 - Suspected ADEM								
evidence_myelitis	1 - Myelitis	2 - Possible myelitis	3 - Myelopathy	4 - Suspected myelopathy							

evidence_gbs	1 - Brighton level 1	2 - Brighton level 2	3 - Brighton level 3	4 - Brighton level 4		
evidence_vasculitis	1 - Definite vasculitis	2 - Possible vasculitis				
neuro_inf_assoc	1-Confirmed/ Strong association	2-Probable	3-Possible	4-Not described in Tab 5 Table 1		
neuro_inf_assoc_details						
neuro_disorder	1 - Yes	0 - No				
neuro_disorder_detail						
headache	1 - Yes	0 - No	2 - Unknown			
reduced_consc	1 - Yes	0 - No	2 - Unknown			
conf	1 - Yes	0 - No	2 - Unknown			
delirium	1 - Yes	0 - No	2 - Unknown			
del_att_aw	1 - Yes	0 - No	2 - Unknown			
del_change	1 - Yes	0 - No	2 - Unknown			
del_cog	1 - Yes	0 - No	2 - Unknown			
del_cog_desc						
behav_change	1 - Yes	0 - No	2 - Unknown			
seizure	1 - Yes	0 - No	2 - Unknown			
seizures_detail	1 - Focal	2 - Generalised	3 - Other			
seizures_detail_other						
visual_symp	1 - Yes	0 - No	2 - Unknown			
visual_symp_detail	1 - Visual field defect	2 - Diplopia	3 - Reduced acuity	4 - Other		
photophobia	1 - Yes	0 - No	2 - Unknown			
neuro_symp_other	1 - Yes	0 - No				
AVPU	1 - Alert	2 - To voice	3 - To pain	4 - Unresponsive		
GCS_total						
GCS_eyes	1 - no response	2 - to pain	3 - to speech	4 - spontaneously		
GCS_verbal	1 - no response	2 - incomprehensible sounds	3 - inappropriate words	4 - confused, but able to answer questions	5 - oriented	
GCS_motor	1 - no response	2 - abnormal extension (decerebrate)	3 - abnormal flexion (decorticate)	4 - flexion withdrawal from pain	5 - localises pain	6 - obeys commands
neck_stiff	1 - Yes	0 - No				
disorient	1 - Yes	0 - No				
witness_seiz	1 - Yes - focal	2 - Yes - generalised	3 - Yes - other	0 - No		
new_movement_dis	1 - Yes	0 - No	2 - Unknown			
new_movement_dis_detail	1 - Ataxia	2 - Chorea	3 - Tremor	4 - Myoclonus	5 - Bradykinesia	6 - Other / combination of features
cran_neuro	1 - Yes	0 - No				
cran_neuro_detail						
perip_neuro	1 - Yes	0 - No				
perip_neuro_detail						
limb_weakness	1 - Yes	0 - No				
limb_weakness_detail						

Sensory_abnorm	1 - Yes	0 - No		
sensory_abnorm_detail				
dysphagia	1 - Yes	0 - No		
dysphasia	1 - Yes	0 - No		
dysphasia_detail				
LP_done	1 - Yes	0 - No		
LP_date				
CSF_OP				
CSF_total_WCC				
CSF_neut				
csf_neut_units	1 - % of total	2 - per µl or cumm or mm^3		
CSF_lymp				
csf_lymp_units	1 - % of total	2 - per µl or cumm or mm^3		
CSF_rbc				
CSF_protein				
csf_protein_units	1 - g/L	2 - mg/dL		
CSF_glucose				
paired_blood_glucose				
csf_glucose_units	1 - mmol/L	2 - mg/dL		
CSF_albumin				
CSF_bands	1 - present (same as serum)	2 - present (different from serum)	3 - present (no serum result)	0 - absent
Other_non_micro_CSF				
gram_stain				
csf_culture				
CSF_HSV	1 - Positive	0 - Negative	2 - Not tested	
CSF_VZV	1 - Positive	0 - Negative	2 - Not tested	
CSF_enterovirus	1 - Positive	0 - Negative	2 - Not tested	
CSF_tb	1 - Positive	0 - Negative	2 - Not tested	
CSF_other_pathogen	1 - Yes	0 - No		
CSF_other_p_desc				
ct_head	1 - Yes	0 - No		
ct_head_result				
mri_brain	1 - Yes	0 - No		
mri_brain_result				
mri_spine	1 - Yes	0 - No		
mri_spine_result				
other_neuro_ex	1 - Yes	0 - No		
eeg	1 - Yes	0 - No		
eeg_result				
NCS_EMG	1 - Yes	0 - No		
ncs_EMG_result				
antin_ab	1 - Yes	0 - No	2 - Unknown	
ab_result				
hypoxia	1 - Yes	0 - No	2 - Unknown	
tx_oxygen				
tx_antivir	1 - Yes	0 - No		
tx_conv_plasma	1 - Yes	0 - No		

tx_toc	1 - Yes	0 - No			
tx_vasopress	1 - Yes	0 - No			
tx_anx	1 - Yes	0 - No			
tx_steroids	1 - Yes	0 - No			
tx_steroids_dose					
tx_steroids_start					
tx_steroids_stop					
tx_steroids_ind					
tx_ivig	1 - Yes	0 - No			
tx_ivig_dose					
tx_ivig_start					
tx_ivig_stop					
tx_ivig_ind					
tx_plex	1 - Yes	0 - No			
tx_plex_start					
tx_plex_stop					
tx_plex_ind					
tx_antic	1 - Yes	0 - No			
tx_antic_dose					
tx_antic_start					
tx_antic_stop					
tx_antic_ind					
tx_antip	1 - Yes	0 - No			
tx_antip_dose					
tx_antip_start					
tx_antip_stop					
tx_antip_ind					
tx_throm	1 - Yes	0 - No			
tx_throm_dose					
tx_throm_start					
tx_thrombec	1 - Yes	0 - No			
tx_thrombec_start					
treatment_other	1 - Yes	0 - No			
treatment_other_detail					
ITU	1 - Yes	0 - No			
date_ad_ITU					
date_dis_ITU					
niv	1 - CPAP	2 - BiPAP	3 - Both CPAP and BiPAP	4 - Other	0 - No
inv_vent	1 - Yes	0 - No			
intub_date					
extub_date					
reintub	1 - Yes	0 - No			
trache	1 - Yes	0 - No			
complications	1 - Yes	0 - No			
complications_desc					
death	1 - Yes	0 - No			
death_date					
death_cause					
autopsy_report					
disch	1 - Yes	0 - No			

discharge_MRS	0 - No symptoms at all	1 - No significant disability despite symptoms; able to carry out all usual duties and activities	2 - Slight disability; unable to carry out all previous activities, but able to look after own affairs without assistance	3 - Moderate disability; requiring some help, but able to walk without assistance	4 - Moderately severe disability; unable to walk without assistance and unable to attend to own bodily needs without assistance	5 - Severe disability; bedridden, incontinent and requiring constant nursing care and attention	6 - Dead
disch_date							
follow_up_date							
covid_neuro_data							
covid_neuro_hospital_total	1 - Yes	0 - No					
covid_neuro_hospital_num							
covid_neuro_time							
covid_hospital_num							
study	1 - Yes	0 - No					
case_def							
pub	1 - Yes	0 - No	2 - submitted/submission planned				
doi							
control	1 - Yes	0 - No					
control_def							
case_control	1 - Yes	0 - No					
data_col	1 - Retrospective collection	2 - Prospective collection					

Section 3: Case definitions

3.1 COVID-19 case definitions

**COVID-19 Case definitions, modified from WHO definitions[1]**			
	**Confirmed**	**Probable**	**Suspected**
**WHO COVID-19 case definitions, adapted from World Health Organization. COVID-19: situation report, 95.[1]**	A person with laboratory confirmation[2] of SARS-CoV-2 infection, irrespective of clinical signs and symptoms. Confirmatory tests include a nucleic acid amplication test (e.g. RT-PCR) or validated antibody test	A suspected case, for whom testing for the COVID-19 virus is inconclusive	A patient with acute respiratory illness (fever and at least one sign/symptom of respiratory distress) AND history of travel to or residence in a location reporting community transmission of COVID-19 disease during the 14 days prior to onset
		**OR**	**OR**
		A suspected case, for whom testing could not be performed for any reason	A patient with acute respiratory illness (fever and at least one sign/symptom of respiratory distress) AND having been in contact with a confirmed or probable case in the last 14 days prior to symptom onset
		**OR**	**OR**
		A suspected case, with a negative RT-PCR test for COVID-19 but ongoing clinical suspicion, with supportive features on blood tests and/or radiological investigations, and no alternative aetiology identified	A patient with severe acute respiratory illness (fever and at least one sign/symptom of respiratory distress AND requiring hospitalisation) AND in the absence of an alternative explanation that fully explains the clinical presentation
			**OR**
			A patient with systemic and/or respiratory features suspected to be due to COVID-19 by the assessing clinician

COVID-19 Disease Severity Scoring[3]		
**Mild disease**		Symptomatic patients (Table 1) meeting the case definition for COVID-19 without evidence of viral pneumonia or hypoxia.<br>See the WHO website for most up-to-date case definitions.[1]
**Moderate disease**	**Pneumonia**	**Adolescent or adult** with clinical signs of pneumonia (fever, cough, dyspnoea, fast breathing) but no signs of severe pneumonia, including SpO2 ≥ 90% on room air[4]<br>**Child** with clinical signs of non-severe pneumonia (cough or difficulty breathing + fast breathing and/or chest indrawing) and no signs of severe pneumonia. Fast breathing (in breaths/min): < 2 months: ≥ 60; 2–11 months: ≥ 50; 1–5 years: ≥ 40 [5]<br>While the diagnosis can be made on clinical grounds; chest imaging (radiograph, CT scan, ultrasound) may assist in diagnosis and identify or exclude pulmonary complications.
**Severe disease**	**Severe pneumonia**	**Adolescent or adult** with clinical signs of pneumonia (fever, cough, dyspnoea, fast breathing) plus one of the following: respiratory rate > 30 breaths/min; severe respiratory distress; or SpO2 < 90% on room air.[4]<br>**Child** with clinical signs of pneumonia (cough or difficulty in breathing) + at least one of the following:<br>• Central cyanosis or SpO2 < 90%; severe respiratory distress (e.g. fast breathing, grunting, very severe chest indrawing); general danger sign: inability to breastfeed or drink, lethargy or unconsciousness, or convulsions [5,6]<br>• Fast breathing (in breaths/min): < 2 months: ≥ 60; 2–11 months: ≥ 50; 1–5 years: ≥ 40 [5]<br>While the diagnosis can be made on clinical grounds; chest imaging (radiograph, CT scan, ultrasound) may assist in diagnosis and identify or exclude pulmonary complications.
**Critical disease**	**Acute respiratory distress syndrome (ARDS)** [7-9]	**Onset:** within 1 week of a known clinical insult (i.e. pneumonia) or new or worsening respiratory symptoms.<br>**Chest imaging:** (radiograph, CT scan, or lung ultrasound): bilateral opacities, not fully explained by volume overload, lobar or lung collapse, or nodules.<br>**Origin of pulmonary infiltrates:** respiratory failure not fully explained by cardiac failure or fluid overload. Need objective assessment (e.g. echocardiography) to exclude hydrostatic cause of infiltrates/oedema if no risk factor present.<br>**Oxygenation impairment in adults** [7,9] **:**<br>• Mild ARDS: 200 mmHg < PaO2/FiO2a ≤ 300 mmHg (with PEEP or CPAP ≥ 5 cmH2O).b<br>• Moderate ARDS: 100 mmHg < PaO2/FiO2 ≤ 200 mmHg (with PEEP ≥ 5 cmH2O).**b**<br>• Severe ARDS: PaO2/FiO2 ≤ 100 mmHg (with PEEP ≥ 5 cmH2O).**b**<br>**Oxygenation impairment in children:** note OI and OSI.**c** Use OI when available. If PaO2 not available, wean FiO2 to maintain SpO2 ≤ 97% to calculate OSI or SpO2/FiO2 ratio:<br>• Bilevel (NIV or CPAP) ≥ 5 cmH2O via full face mask: PaO2/FiO2 ≤ 300 mmHg or SpO2/FiO2 ≤ 264.<br>• Mild ARDS (invasively ventilated): 4 ≤ OI < 8 or 5 ≤ OSI < 7.5.<br>• Moderate ARDS (invasively ventilated): 8 ≤ OI < 16 or 7.5 ≤ OSI < 12.3.<br>• Severe ARDS (invasively ventilated): OI ≥ 16 or OSI ≥ 12.3.
**Critical disease**	**Sepsis** [10,11]	**Adults:** acute life-threatening organ dysfunction caused by a dysregulated host response to suspected or proven infection. Signs of organ dysfunction include: altered mental status, difficult or fast breathing, low oxygen saturation, reduced urine output [10], fast heart rate, weak pulse, cold extremities or low blood pressure, skin mottling, laboratory evidence of coagulopathy, thrombocytopenia, acidosis, high lactate, or hyperbilirubinemia.<br>**Children:** suspected or proven infection and ≥ 2 age-based systemic inflammatory response syndrome (SIRS) criteria,**e** of which one must be abnormal temperature or white blood cell count.
	**Septic shock** [10,11]	**Adults**: persistent hypotension despite volume resuscitation, requiring vasopressors to maintain MAP ≥ 65 mmHg and serum lactate level > 2 mmol/L.<br>**Children:** any hypotension (SBP < 5th centile or > 2 SD below normal for age) or two or three of the following: altered mental status; bradycardia or tachycardia (HR < 90 bpm or > 160 bpm in infants and heart rate < 70 bpm or > 150 bpm in children); prolonged capillary refill (> 2 sec) or weak pulse; fast breathing; mottled or cool skin or petechial or purpuric rash; high lactate; reduced urine output; hyperthermia or hypothermia.[12,13].

Other complications that have been described in COVID-19 patients include acute, life-threatening conditions such as: acute pulmonary embolism, acute coronary syndrome, acute stroke and delirium. Clinical suspicion for these complications should be heightened when caring for COVID-19 patients, and appropriate diagnostic and treatment protocols available.

a If altitude is higher than 1000 m, then the correction factor should be calculated as follows: PaO2/FiO2 x barometric pressure/760.

b When PaO2 is not available, SpO2/FiO2 ≤ 315 suggests ARDS (including in non-ventilated patients).

c Oxygenation Index (OI) is an invasive measurement of the severity of hypoxaemic respiratory failure and may be used to predict outcomes in paediatric patients. It is calculated as follows: percentage of fraction of inhaled oxygen multiplied by the mean airway pressure (in mmHg), divided by the partial pressure of arterial oxygen (in mmHg). Oxygen saturation index (OSI) is a non-invasive measurement and has been shown to be a reliable surrogate marker of OI in children and adults with respiratory failure. OSI replaces PaO2 with oxygen saturation as measured by pulse oximetry (SpO2) in the OI equation.

d The SOFA score ranges from 0 to 24 and includes points related to six organ systems: respiratory (hypoxaemia defined by low PaO2/FiO2); coagulation (low platelets); liver (high bilirubin); cardiovascular (hypotension); central nervous system (low level of consciousness defined by Glasgow Coma Scale); and renal (low urine output or high creatinine). Sepsis is defined by an increase in the sepsis-related SOFA score of ≥ 2 points. Assume the baseline score is 0 if data are not available. [14]

e SIRS criteria: abnormal temperature (> 38.5 °C or < 36 °C); tachycardia for age or bradycardia for age if < 1 year; tachypnoea for age or need for mechanical ventilation; abnormal white blood cell count for age or > 10% bands.

Abbreviations: BP blood pressure; bpm beats per minute; CPAP continuous positive airway pressure; CT computed tomography; FiO2 fraction of inspired oxygen; MAP mean arterial pressure; NIV non-invasive ventilation; OI Oxygenation Index; OSI Oxygenation Index using SpO2; PaO2 partial pressure arterial oxygen; PEEP positive end-expiratory pressure; SBP systolic blood pressure; SD standard deviation; SIRS systemic inflammatory response syndrome; SOFA sequential organ failure assessment; SpO2 oxygen saturation.

References

1. World Health Organization. COVID-19: situation report, 95. Link: https://www.who.int/docs/default-source/coronaviruse/situation-reports/20200424-sitrep-95-covid-19.pdf?sfvrsn=e8065831_4
2. WHO. World Health Organization. Laboratory testing for coronavirus disease 2019 (COVID-19) in suspected human cases. Interim Guide 2020., Link: https://www.who.int/emergencies/diseases/novel-coronavirus-2019/technical-guidance/laboratory-guidance
3. World Health Organization, 2020. Clinical management of COVID-19: interim guidance, 27 May 2020 (No. WHO/2019-nCoV/clinical/2020.5). World Health Organization.
4. IMAI District Clinician Manual. Hospital care for adolescents and adults. Geneva: World Health Organization; 2020 (Link:https://apps.who.int/iris/bitstream/handle/10665/77751/9789241548290_Vol2_eng.pdf?sequence=3, accessed 13 May 2020).
5. Pocket book of hospital care for children: Guidelines for the management of common childhood illnesses: Second edition. Geneva: World Health Organization; 2013 (Link: https://www.who.int/maternal_child_adolescent/documents/child_hospital_care/en/, accessed 13 May 2020).
6. Russell FM, Reyburn R, Chan J, Tuivaga E, Lim R, Lai J, et al. Impact of the change in WHO's severe pneumonia case definition on hospitalized pneumonia epidemiology: case studies from six countries. Bull World Health Organ. 2019;97(6):386-93. Epub 2019/06/19.
7. Force ADT, Ranieri VM, Rubenfeld GD, Thompson BT, Ferguson ND, Caldwell E, et al. Acute respiratory distress syndrome: the Berlin Definition. JAMA. 2012;307(23):2526-33. Epub 2012/07/17.
8. Khemani RG, Smith LS, Zimmerman JJ, Erickson S, Pediatric Acute Lung Injury Consensus Conference Group. Pediatric acute respiratory distress syndrome: definition, incidence, and epidemiology: proceedings from the Pediatric Acute Lung Injury Consensus Conference. Pediatr Crit Care Med. 2015;16(5 Suppl 1):S23-40. Epub 2015/06/04.
9. Riviello ED, Kiviri W, Twagirumugabe T, Mueller A, Banner-Goodspeed VM, Officer L, et al. Hospital Incidence and Outcomes of the Acute Respiratory Distress Syndrome Using the Kigali Modification of the Berlin Definition. Am J Respir Crit Care Med. 2016;193(1):52-9. Epub 2015/09/10.
10. Sepsis and Septic Shock: 2016. Intensive Care Med. 2017;43(3):304-77. Epub 2017/01/20.
11. Weiss SL, Peters MJ, Alhazzani W, Agus MSD, Flori HR, Inwald DP, et al. Surviving Sepsis Campaign International Guidelines for the Management of Septic Shock and Sepsis-associated Organ Dysfunction in Children. Intensive Care Med. 2020;46(Suppl 1):10-67. Epub 2020/02/08.
12. Goldstein B, Giroir B, Randolph A, International Consensus Conference on Pediatric Sepsis. International pediatric sepsis consensus conference: definitions for sepsis and organ dysfunction in pediatrics. Pediatr Crit Care Med. 2005;6(1):2-8. Epub 2005/01/08.
13. Davis AL, Carcillo JA, Aneja RK, Deymann AJ, Lin JC, Nguyen TC, et al. American College of Critical Care Medicine Clinical Practice Parameters for Hemodynamic Support of Pediatric and Neonatal Septic Shock. Crit Care Med. 2017;45(6):1061-93. Epub 2017/05/17.
14. Vincent JL, Moreno R, Takala J, Willatts S, De Mendonca A, Bruining H, et al. The SOFA (Sepsis-related Organ Failure Assessment) score to describe organ dysfunction/failure. On behalf of the Working Group on Sepsis-Related Problems of the European Society of Intensive Care Medicine. Intensive Care Med. 1996;22(7):707-10. Epub 1996/07/01.

3.2 Neurological case definitions

<table>
<tr><th colspan="4">1. Meningitis and meningism[1]</th></tr>
<tr><th>Level 1
Meningitis</th><th>Level 2
Possible meningitis</th><th>Level 3
Meningism</th><th>Level 4
Suspected meningitis</th></tr>
<tr><td colspan="4">[ ] Suspected meningitis with no other diagnoses apparent, but does not fulfil level 3 criteria</td></tr>
<tr><td colspan="3">[ ] Absence of an alternative diagnosis for symptoms
AND
[ ] Neck stiffness
OR
[ ] Kernig’s sign positive
OR
[ ] Brudzinsky’s sign positive</td><td rowspan="3"></td></tr>
<tr><td colspan="2">[ ] Fever (≥ 38ºC)</td><td rowspan="2"></td></tr>
<tr><td>[ ] CSF total white cell count > 5 cells/mm^3
OR
[ ] Meningeal enhancement seen on contrast enhanced CT or MRI</td><td></td></tr>
<tr><td>[ ] Level 1
Meningitis</td><td>[ ] Level 2
Possible meningitis</td><td>[ ] Level 3
Meningism</td><td>[ ] Level 4
Suspected meningitis</td></tr>
</table>

<table>
<tr><td colspan="4">2. Encephalitis and encephalopathy (including delirium)[2,3,4]</td></tr>
<tr><td>Level 1
Encephalitis*</td><td>Level 2
Possible encephalitis</td><td>Level 3
Encephalopathy^</td><td>Level 4
Suspected encephalopathy</td></tr>
<tr><td colspan="4">[ ] Suspected encephalopathy with no other diagnosis apparent, but does not fulfill level 3 criteria</td></tr>
<tr><td colspan="3">[ ] Acute or sub acute (<4 weeks) alteration in consciousness, cognition (including delirium^{+}), personality or behaviour persisting for more than 24 hours

AND
[ ] Absence of an alternative diagnosis for symptoms</td><td rowspan="3"></td></tr>
<tr><td colspan="2">[ ] New onset seizure
OR
[ ] New focal neurological signs
OR
[ ] Fever (≥ 38ºC)
OR
[ ] Movement disorder (includes: Parkinsonism, oromotor dysfunction etc.)
OR
[ ] EEG consistent with focal abnormality</td><td rowspan="2"></td></tr>
<tr><td>[ ] CSF total white cell count > 5 cells/mm^3
OR
[ ] Neuroimaging compatible with encephalitis
OR
[ ] Confirmation of brain inflammation on brain biopsy</td><td></td></tr>
<tr><td>[ ] Level 1
Encephalitis*</td><td>[ ] Level 2
Possible encephalitis</td><td>[ ] Level 3
Encephalopathy^</td><td>[ ] Level 4
Suspected encephalopathy</td></tr>
<tr><td colspan="4">*Encephalitis is inflammation of the brain parenchyma;</td></tr>
</table>

^**Encephalopathy** is a rapidly developing pathobiological process in the brain that can lead to a change in consciousness, cognition (with a clinical presentation of delirium or coma), personality or behaviour persisting for more than 24 hours.

+**Delirium**[4] is defined as a clinical manifestation of encephalopathy, when the following features are present:

A) Disturbance in attention (reduced ability to direct, focus, sustain, and shift attention) and awareness (reduced orientation to the environment);

B) Develops over a short period of time, represents a change from baseline attention and awareness, and tends to fluctuate in severity during the course of the day

C) An additional disturbance in cognition (e.g., memory deficit, disorientation, language, visuospatial ability, or perception).

D) Criteria A and C are not explained by another pre-existing, established, or evolving neurocognitive disorder, and do not occur in the context of a severely reduced level of arousal, such as coma.

E) There is evidence from the history, physical examination, or laboratory findings that the disturbance is a direct physiologic consequence of another medical condition, substance intoxication or withdrawal, or exposure to a toxin, or is because of multiple etiologies.

12

<table>
<tr><td colspan="4">3. Myelitis and myelopathy[5]</td></tr>
<tr><td>Level 1

Myelitis</td><td>Level 2

Possible myelitis</td><td>Level 3

Myelopathy</td><td>Level 3

Suspected myelopathy</td></tr>
<tr><td colspan="4">[ ] Weakness or sensory disturbance of upper and/or lower limbs, developing to its worst severity between 4h and 21d following onset of symptoms *</td></tr>
<tr><td colspan="3">[ ] Absence of an alternative diagnosis for symptoms

WITH

[ ] Brisk reflexes or extensor plantar response
OR
[ ] Bladder or bowel dysfunction
OR
[ ] Clearly defined sensory level</td><td rowspan="3"></td></tr>
<tr><td colspan="2">[ ] Absence of extra-axial compressive aetiology by neuroimaging (MRI or CT myelography)

AND

[ ] Absence of flow voids on the surface of the spinal cord suggestive of arteriovenous malformation (MRI)</td><td rowspan="2"></td></tr>
<tr><td>[ ] CSF total white cell count >5 cells/mm^3
OR

[ ] MRI changes consistent with myelitis (gadolinium enhancement or T2 hyperintensity)

OR

[ ] Elevated CSF IgG index</td><td></td></tr>
<tr><td>[ ] Level 1
Myelitis</td><td>[ ] Level 2
Possible myelitis</td><td>[ ] Level 3
Myelopathy</td><td>[ ] Level 4
Suspected myelopathy</td></tr>
</table>

13

<table>
<tr><th colspan="3">4. Acute Disseminated Encephalomyelitis (ADEM)[6,7]</th></tr>
<tr><td>Level 1
ADEM</td><td>Level 2
Probable ADEM</td><td>Level 3
Suspected ADEM</td></tr>
<tr><td colspan="3">[ ] Suspected ADEM with no other diagnosis apparent, but does not fit level 2 criteria</td></tr>
<tr><td colspan="2">[ ] first multifocal clinical CNS event
AND
[ ] alteration in consciousness or behavioural change (encephalopathy) unexplained by fever/systemic illness/postictal symptoms
AND
[ ] abnormal brain MRI with typical diffuse, poorly demarcated lesions >1cm</td><td rowspan="2"></td></tr>
<tr><td>[ ] no new clinical or MRI findings 3 months or more after symptom onset
OR
[ ] signs/symptoms/MRI findings consistent with multiphasic ADEM*</td><td></td></tr>
<tr><td>[ ] Level 1
ADEM</td><td>[ ] Level 2
Probable ADEM</td><td>[ ] Level 3
Suspected ADEM</td></tr>
<tr><td colspan="3">*Multiphasic ADEM – two episodes of Level 2 ADEM separated by three months but not followed by any further events. The second ADEM event can involve either new or re-emergence of prior neurological symptoms/signs/MRI findings. Beyond a second encephalopathic event, the diagnosis is no longer consistent with multiphasic ADEM.</td></tr>
</table>

14

<table>
<tr><th colspan="4">5. Guillain-Barré Syndrome[8]</th></tr>
<tr><td>Level 1</td><td>Level 2</td><td>Level 3</td><td>Level 4</td></tr>
<tr><td colspan="4">Suspected Guillain-Barré syndrome with no other diagnosis apparent, but does not fulfill level 3 criteria</td></tr>
<tr><td colspan="3">[ ] Bilateral and flaccid weakness of the limbs
AND
[ ] Absence of an alternative diagnosis for weakness
AND
[ ] Decreased or absent deep tendon reflexes in affected limbs
AND
[ ] Monophasic illness pattern with weakness nadir between 12 hours and 28 days, followed by clinical plateau</td><td rowspan="3"></td></tr>
<tr><td colspan="2">[ ]CSF total white cell count < 50 cells/mm^3
OR
[ ]If CSF results unavailable, electrophysiological findings consistent with GBS</td><td rowspan="2"></td></tr>
<tr><td>[ ]CSF protein level above laboratory normal value AND CSF total white cell count < 50 cells/mm^3
AND
[ ] Electrophysiological findings consistent with GBS</td><td></td></tr>
<tr><td>[ ] Level 1</td><td>[ ] Level 2</td><td>[ ] Level 3</td><td>[ ] Level 4</td></tr>
</table>

6. Cerebrovascular disease a) Stroke and Transient Ischaemic Attack[9, 10]	
**Definitions**	
Central Nervous System (CNS) infarction	Brain, spinal cord, or retinal cell death attributable to ischemia, based on:<br>[ ] clinical evidence of cerebral, spinal cord, or retinal focal ischemic injury based on symptoms persisting ≥24 hours or until death, and other etiologies excluded (CNS infarction includes hemorrhagic infarctions, types I and II, see 'Intracerebral hemorrhage')<br>**OR**<br>[ ] pathological, imaging, or other objective evidence of cerebral, spinal cord, or retinal focal ischemic injury in a defined vascular distribution
Silent CNS infarction	[ ] Imaging or neuropathological evidence of CNS infarction, without a history of acute neurological dysfunction attributable to the lesion.
Ischemic stroke	[ ] An episode of neurological dysfunction caused by focal cerebral, spinal, or retinal infarction.
Intracerebral hemorrhage	[ ] A focal collection of blood within the brain parenchyma or ventricular system that is not caused by trauma. (Intracerebral hemorrhage includes parenchymal hemorrhages after CNS infarction, type I - petechiae of blood along the margins of the infarction, and type II - confluent petechiae within the infarction but without a space-occupying effect.)
Silent cerebral hemorrhage	[ ] A focal collection of chronic blood products within the brain parenchyma, subarachnoid space, or ventricular system on neuroimaging or neuropathological examination that is not caused by trauma and without a history of acute neurological dysfunction attributable to the lesion.
Stroke caused by intracerebral hemorrhage	[ ] Rapidly developing clinical signs of neurological dysfunction attributable to a focal collection of blood within the brain parenchyma or ventricular system that is not caused by trauma.
Subarachnoid hemorrhage	[ ] Bleeding into the subarachnoid space (the space between the arachnoid membrane and the pia mater of the brain or spinal cord)
Stroke caused by subarachnoid hemorrhage	[ ] Rapidly developing signs of neurological dysfunction and/or headache because of bleeding into the subarachnoid space (the space between the arachnoid membrane and the pia mater of the brain or spinal cord), which is not caused by trauma.
Stroke caused by cerebral venous thrombosis	[ ] Infarction or hemorrhage in the brain, spinal cord, or retina because of thrombosis of a cerebral venous structure. Symptoms or signs caused by reversible edema without infarction or hemorrhage do not qualify as stroke.
Stroke, not otherwise specified	[ ] An episode of acute neurological dysfunction presumed to be caused by ischemia or hemorrhage, persisting ≥24 hours or until death, but without sufficient evidence to be classified as one of the above.
Transient ischemic attack (TIA)	[ ] A transient episode of neurological dysfunction caused by focal brain, spinal cord, or retinal ischemia, without acute infarction

6. Cerebrovascular disease b) Central nervous system (CNS) vasculitis[11]		
	**Definite**	**Possible**
Central Nervous System (CNS) vasculitis	Clinical presentation compatible with CNS vasculitis with exclusion of alternative possible diagnoses and of primary systemic vasculitic syndrome<br><br>**AND**<br><br>Positive CNS histology (biopsy or autopsy) showing CNS angiitis (granulomatous, lymphocytic or necrotising), including evidence of vessel wall damage.	Clinical presentation compatible with CNS vasculitis with exclusion of alternative possible diagnoses and of primary systemic vasculitic syndrome<br><br>**AND**<br>Laboratory and imaging support for CNS inflammation (elevated levels of CSF protein and/or cells, and/or the presence of oligoclonal bands and/or MR scan evidence compatible with CNS vasculitis), with angiographic* exclusion of other specific entities, but without histological proof of vasculitis.
*Certain disorders, perhaps most particularly moyamoya disease, may require formal contrast angiography for definitive diagnosis.		

7. Other neurological syndrome
Any new onset neurological disease suspected by the clinician to be associated with recent COVID-19 infection, including neuropsychiatric disease (psychosis, affective disorders), complications of critical illness (myopathy, neuropathy), and olfactory dysfunction (anosmia, ageusia etc.) with no other neurological features.

Tables 1-5 from Mehta et al.[12]

References

1. McGill F, Heyderman RS, Michael BD, et al. The UK joint specialist societies guideline on the diagnosis and management of acute meningitis and meningococcal sepsis in immunocompetent adults. J Infect 2016;72:405-38.
2. Venkatesan A, Tunkel AR, Bloch KC, *et al.* Case Definitions, Diagnostic Algorithms, and Priorities in Encephalitis: Consensus Statement of the International Encephalitis Consortium. *Clin Infect Dis* 2013; 57: 1114–28.
3. Granerod J, Ambrose HE, Davies NWS, *et al.* Causes of encephalitis and differences in their clinical presentations in England: A multicentre, population-based prospective study. *Lancet Infect Dis* 2010. 10(12): 835-44.
4. Slooter, Arjen JC, et al. "Updated nomenclature of delirium and acute encephalopathy: statement of ten Societies." Intensive care medicine (2020): 1-3.
5. Transverse Myelitis Consortium Working Group. Proposed diagnostic criteria and nosology of acute transverse myelitis. *Neurology* 2002; **59**: 499–505.
6. Krupp LB, Tardieu M, Amato MP, *et al.* International Pediatric Multiple Sclerosis Study Group criteria for pediatric multiple sclerosis and immune-mediated central nervous system demyelinating disorders: Revisions to the 2007 definitions. Mult. Scler. J. 2013. 19(10):1261-7
7. Sejvar JJ, Kohl KS, Bilynsky R, *et al.* Encephalitis, myelitis, and acute disseminated encephalomyelitis (ADEM): Case definitions and guidelines for collection, analysis, and presentation of immunization safety data. *Vaccine* 2007. 25(31):5771-92
8. Sejvar JJ, Kohl KS, Gidudu J, *et al.* Guillain-Barré syndrome and Fisher syndrome: Case definitions and guidelines for collection, analysis, and presentation of immunization safety data. *Vaccine* 2011. 29(3):599-612.
9. Sacco RL, Kasner SE, Broderick JP, *et al.* An Updated Definition of Stroke for the 21st Century. *Stroke* 2013; **44**: 2064–89.
10. Easton JD, Saver JL, Albers GW, *et al.* Definition and Evaluation of Transient Ischemic Attack. *Stroke* 2009; **40**: 2276–93.
11. Rice CM, Scolding NJ. The diagnosis of primary central nervous system vasculitis, *Practical Neurology* 2020;20:109-114.
12. Mehta R, Soares CN, Medialdea-Carrera R, et al. The spectrum of neurological disease associated with Zika and chikungunya viruses in adults in Rio de Janeiro, Brazil: A case series. PLoS Negl Trop Dis. 2018;12(2):e0006212.

3.3 COVID-19: association between viral infection and neurological disease

	Confirmed	Probable	Possible
**SARS-CoV-2 meningitis, encephalitis, CNS vasculitis, myelitis/myelopathy****	1. SARS-CoV-2 detected in CSF/ brain tissue †,<br>**OR**<br>Evidence of SARS-CoV-2-specific intrathecal antibody;<br>**AND**<br>2. No other explanatory pathogen or cause found	1. SARS-CoV-2 detected in respiratory or other non-CNS sample ‡,<br>**OR**<br>Evidence of SARS-CoV-2-specific antibody in serum indicating acute infection+§;<br>**AND**<br>2. No other explanatory pathogen or cause found	Patient meets suspected case definition of COVID-19 according to national or WHO guidance (as below), based on clinical symptoms and epidemiological risk factors.<br>In the context of known community SARS-CoV-2 transmission, supportive features* include:<br>*Clinical:* new onset of least one of: cough, fever, shortness of breath, muscle aches, loss of smell, loss of taste;<br>*Laboratory*: lymphopenia, raised d-dimer;<br>*Radiological*: evidence of abnormalities consistent with infection or inflammation (e.g. ground glass changes)
	**Strong association**	**Probable association**	**Possible association**
**Acute disseminated encephalomyelitis** (ADEM) associated with SARS-CoV-2 infection**		1. Neurological disease onset <= 6 weeks after acute infection,<br>**AND**<br>2. SARS-CoV-2 RNA detected in any sample,<br>**OR**<br>Antibody evidence of acute SARS-CoV-2 infection;<br>**AND**<br>3. No evidence of other commonly associated causes	1. Neurological disease onset <= 6 weeks after acute infection,<br>**AND**<br>2. SARS-CoV-2 RNA detected in any sample;<br>**OR**<br>Antibody evidence of acute SARS-CoV-2 infection;<br>**AND**<br>3. Evidence of other commonly associated causes

Strong association		Probable association	Possible association
**Guillain-Barré syndrome** and other acute neuropathies associated with SARS-CoV-2 infection**		1. Neurological disease onset <= 6 weeks after acute infection,<br><br>**AND**<br><br>2. SARS-CoV-2 RNA detected in any sample;<br>**OR**<br>Antibody evidence of acute SARS-CoV-2 infection;<br><br>**AND**<br><br>**3.** No evidence of other commonly associated causes ¶	1. Neurological disease onset <= 6 weeks after acute infection,<br><br>**AND**<br><br>2. SARS-CoV-2 RNA detected in any sample;<br>**OR**<br>Antibody evidence of acute SARS-CoV-2 infection;<br><br>**AND**<br><br>3. Evidence of other commonly associated causes ¶
**Stroke** associated with SARS-CoV-2 infection**		1. SARS-CoV-2 detected in CSF or other sample ‡;<br><br>**OR**<br><br>Evidence of SARS-CoV-2-specific antibody in serum indicating acute infection;<br><br>**AND**<br><br>2. No other known traditional cardiovascular risk factors ¥	1. SARS-CoV-2 detected in CSF or other sample;<br><br>**OR**<br><br>Evidence of SARS-CoV-2-specific antibody indicating acute infection;<br><br>**AND**<br><br>2. Other traditional cardiovascular risk factors ¥

*These case definitions are suggestions based on published information to date; they are likely to need refining as more data emerge.
† detection in CSF or brain tissue by PCR, culture, or immunohistochemistry, as appropriate; ‡ detection in non-CNS sample by PCR or culture. § Serological evidence of acute infection can be defined as i) detection of IgM, or ii) IgG seroconversion or iii) >=4-fold rise in antibody titres in paired acute and convalescent serum samples. ¶ These include: infection with one of Campylobacter jejuni, Mycoplasma pneumoniae, Cytomegalovirus (CMV), Epstein–Barr virus (EBV), hepatitis E virus, Zika virus, and HIV; or vaccination in the last 6 weeks. Associated causes may differ depending on geographical location. ¥ traditional cardiovascular risk factors include; hypertension, current smoker, diabetes, hypercholesterolemia, and atrial fibrillation.

The terms 'confirmed', 'probable' and 'suspected' are used in the WHO COVID-19 case definition. The terms 'confirmed', 'probable' and 'possible' for COVID-19 meningitis, encephalitis or myelitis and 'strong association', 'probable association', 'possible association' reflect the terminology used for the different syndromes in the original publications from which this table derives (see table references).

References

1. Granerod J, Cunningham R, Zuckerman M, et al. Causality in acute encephalitis: defining aetiologies. Epidemiol Infect 2010. DOI:10.1017/s0950268810000725.
2. Solomon T, Dung NM, Vaughn DW, et al. Neurological manifestations of dengue infection. Lancet 2000; 355: 1053–9.
3. Mehta R et al. The spectrum of neurological disease associated with Zika and chikungunya viruses in adults in Rio de Janeiro, Brazil: A case series. PLoS Negl Trop Dis 2018; 12: e0006212.
